# Risk Adjustment for Alzheimer Disease and Related Dementias in Medicare Advantage and Health Care Experiences

**DOI:** 10.1001/jamanetworkopen.2026.1796

**Published:** 2026-03-13

**Authors:** Wei Fu, Yuting Qian, Seyed M. Karimi, Hamid Zarei, Xi Chen

**Affiliations:** 1 Department of Health Management and Systems Sciences, University of Louisville, Louisville, Kentucky; 2 Department of Health Policy and Management, Yale School of Public Health, New Haven, Connecticut; 3 National Bureau of Economic Research, Cambridge, Massachusetts

## Abstract

**Importance:**

Failure to account for the full complexity and costs of high-need populations in the risk-adjusted capitated payment model for Medicare Advantage (MA) plans may create financial disincentives for plans to invest in comprehensive care for affected beneficiaries, potentially exacerbating health disparities.

**Objective:**

To evaluate the association of reinstatement of Alzheimer disease and related dementias (ADRD) hierarchical condition categories (HCCs) into the MA risk-adjusted payment model in 2020 with access, affordability, and quality of care for beneficiaries with ADRD.

**Design, Setting, and Participants:**

This cross-sectional study examined a nationally representative sample of MA beneficiaries from the Medicare Current Beneficiary Survey (2015-2022). Beneficiaries with ADRD and those without ADRD but with comparable neurological diseases (stroke, paralysis, or Parkinson disease) before and after 2020 were included. Data analyses were performed between January and December 2025.

**Exposures:**

Reinstatement of the ADRD HCC into the MA risk adjustment formula in 2020.

**Main Outcomes and Measures:**

Primary outcomes were accessibility of needed care, medical financial burden, satisfaction with specialist access, and satisfaction with quality of care. These outcomes were assessed using a difference-in-differences model to compare changes between the treatment and control group before and after the inclusion of ADRD HCCs in the MA risk adjustment model in 2020.

**Results:**

Among 5353 MA beneficiary observations (1239 [23.1%] aged 65-74 years; 3127 [58.4%] aged ≥75 years; 1785 male [33.3%]), 1629 (30.4%) reported a diagnosis of ADRD, and 3724 (69.6%) did not report an ADRD diagnosis. Compared with MA beneficiaries without ADRD, those with ADRD reported lower rates of difficulty accessing care (142 beneficiaries [8.7%] vs 394 beneficiaries [10.6%]) and medical financial burden (235 beneficiaries [19.3%] vs 740 beneficiaries [25.1%]), but slightly lower rates of satisfaction with specialist access (1384 beneficiaries [90.8%] vs 3267 [92.7%]) and care quality (1495 beneficiaries [92.8%] vs 3414 beneficiaries [93.0%]). Reintroducing ADRD HCCs into the MA risk-adjusted payment model was associated with a 6.62 percentage-point decrease in reporting any troubles accessing needed care (β = 0.06; 95% CI, −0.11 to −0.02; *P* = .005) and a 9.20 percentage-point decrease in reporting any medical financial burden (β = −0.09; 95% CI, −0.16 to −0.02; *P* = .009) among MA beneficiaries with ADRD. No significant association was observed for satisfaction with specialist access or with quality of care among MA beneficiaries with ADRD.

**Conclusions and Relevance:**

In this cross-sectional study of MA beneficiaries, reintroducing ADRD HCCs into the MA risk adjustment model was associated with improved care access and reduced financial burden among MA beneficiaries with ADRD. These findings suggest that risk adjustment that better reflects the costs of chronic, complex conditions may better align MA plan incentives with the needs of high-need populations and promote care equity.

## Key Points

**Question:**

Was reinstating Alzheimer disease and related dementias (ADRD) hierarchical condition categories (HCCs) into the Medicare Advantage (MA) risk-adjusted payment model in 2020 associated with changes in care experiences for beneficiaries with ADRD?

**Findings:**

In this cross-sectional study of 5353 MA observations (2015-2022), reinstating the ADRD HCC was associated with a 6.6 percentage-point decline in reported care access difficulties and a 9.2 percentage-point decline in reported medical financial burden, with no significant changes in satisfaction with specialist access and satisfaction with care quality.

**Meaning:**

These findings suggest that risk adjustment models that more accurately reflect the costs of complex chronic conditions may promote health equity.

## Teaser

This cross-sectional study analyzes differences in health care experiences after the reinstatement of Alzheimer disease and related dementias (ADRD) hierarchical condition categories into the Medicare Advantage risk-adjusted payment model in 2020.

## Introduction

Alzheimer disease and related dementias (ADRD) are among the leading causes of disability and dependence among older adults, affecting approximately 6.9 million individuals in the US, placing a substantial burden on patients, caregivers, and the health care system, with annual costs exceeding $300 billion in 2024.^1^ Medicare Advantage (MA) plans, which now enroll more than one-half of all Medicare beneficiaries,^2^ offer a unique approach to addressing the needs of this vulnerable population through enhanced care management programs. Approximately one-third of Medicare-eligible beneficiaries with ADRD in 2018 enrolled in an MA plan, increasing from 22% in 2013.^2^ However, MA plans have historically faced challenges in providing equitable and high-quality care for beneficiaries with ADRD.^2,3,4,5,6,7^

Different from Medicare fee-for-service (FFS), the risk adjustment model used by the Centers for Medicare & Medicaid Services (CMS) plays a critical role in incentivizing MA plans to deliver care tailored to beneficiaries’ health needs. MA plans are privately operated and receive prospective, capitated payments from CMS to finance and deliver health care services. The capitated payment is predicted through a risk adjustment model that accounts for a beneficiary’s demographics and a selected set of hierarchical condition categories (HCCs). From 2014 to 2020, ADRD diagnoses were excluded from the risk adjustment model due to concerns about coding and auditing claims accuracy.^8^ Because insurers bear increasing financial risks, for beneficiaries with HCCs not incorporated in the risk-adjusted payment model, the capitated payments may incentivize insurers to manage these beneficiaries’ medical costs by providing insufficient coverage, restricting physician networks, or requiring prior authorization for care.^9^

In 2020, CMS reinstated ADRD in the risk-adjusted payment model, marking a substantial policy shift. This decision offers a unique opportunity to examine the association of the new risk-adjusted payment model with care experiences among beneficiaries with ADRD. Recent studies have found a significant increase in the ADRD diagnosis rate among MA beneficiaries after this payment model change.^2,8,10^ However, there remains limited empirical evidence on the broader implications of this payment model change for care access, affordability, and quality among beneficiaries with ADRD.

## Methods

### Data and Sample Inclusion

This cross-sectional study used data from the nationally representative Medicare Current Beneficiary Survey Public Use Files (MCBS PUF; 2015-2022). The MCBS includes both FFS and MA beneficiaries aged 65 years and older, as well as beneficiaries younger than 65 years with disabling conditions residing in the continental US. It provides rich information on beneficiaries’ care experiences about services access and obstacles encountered. This study was waived from institutional board review and the requirement for informed consent by the Human Research Protection Program at Yale University because it was considered not to involve human participation due to its use of secondary data. The study adhered to the Strengthening the Reporting of Observational Studies in Epidemiology (STROBE) reporting guideline.

**Figure 1.**
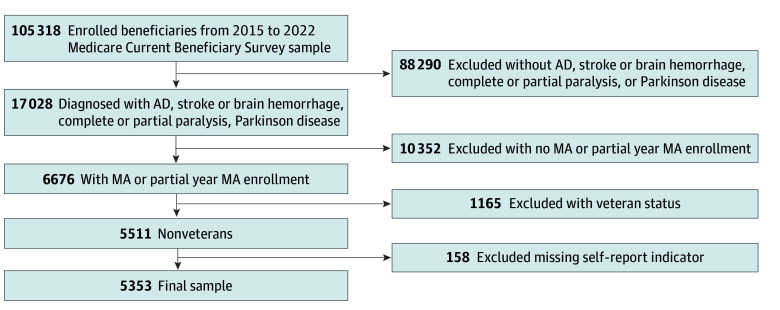
Flowchart of Sample Selection Processes The main sample included beneficiaries enrolled in any Medicare Advantage (MA) plan. AD indicates Alzheimer disease.

Our main sample included beneficiaries enrolled in any MA plan. MA enrollees with ADRD were assigned as the treatment group, and MA enrollees without ADRD but with stroke or brain hemorrhage, complete or partial paralysis, or Parkinson disease were assigned as the control group. These control group conditions were selected because, similar to ADRD, they often require specialized neurological care and are therefore more comparable to the treatment group with respect to patterns of health care utilization. Unlike ADRD, these conditions were already included in the risk-adjustment model before 2020. To address confounding due to plan-switch following the inclusion of ADRD HCCs in 2020, we excluded beneficiaries with only partial MA enrollment in the year preceding the survey. We also excluded veterans. Our sample thus is a repeated cross-sectional dataset of beneficiary-survey-year observations from 2015 to 2022 with complete data on all covariates (Figure 1).

### Variables

We used information regarding MA plan enrollment to define MA status. ADRD and conditions for the control group were identified via self-reported physician diagnosis. Due to data limitations, we could not ascertain the timing of diagnosis or the initial MA enrollment.

We used information on whether a respondent reported any trouble obtaining needed care to measure accessibility of needed care. We defined a respondent as experiencing a medical financial burden if they reported problems paying medical bills (available since 2017) or expressed dissatisfaction with their out-of-pocket costs. Additionally, we examined 2 binary measures of satisfaction with care: satisfaction with specialist access and satisfaction with the quality of care. Covariates included demographic variables (age, gender, marital status, race and ethnicity, education, and household size) and health-related measures (body mass index, number of chronic conditions, and indicators for functional limitations). Self-reported race and ethnicity included non-Hispanic Black, Hispanic, non-Hispanic White, and other (any race or ethnicity not otherwise specified); race and ethnicity were included as a covariate to account for existing differences in care utilization across racial or ethnic groups.

### Statistical Analysis

We employed a difference-in-differences (DID) model to compare changes in care experiences between the treatment and control group before and after the inclusion of ADRD HCCs in the MA risk adjustment model in 2020 (eAppendix 1 in Supplement 1). We adjusted the model by interacting the covariates with year indicators to mitigate biases arising from contemporaneous shocks (eg, COVID-19). To address measurement errors in self-reported care experiences among beneficiaries with ADRD, we interacted covariates and year indicators with an indicator for surveys completed by proxy (eg, family members), respectively. All analyses used heteroskedasticity-robust standard errors.

We conducted an event study analysis within the same framework, using 2019 as the reference year—the last year before the inclusion of ADRD HCC (eAppendix 2 in Supplement 1). This analysis provides suggestive evidence for the parallel-trends assumption of the DID model and examines temporal association dynamics following the new payment model.

We conducted various robust and placebo tests (eAppendix 3 and eAppendix 4 in Supplement 1). In one illustrative placebo test, we designated MA enrollees with diabetes (but without ADRD) as a negative control group and MA enrollees without either condition as the comparison group, hypothesizing no association of the 2020 risk adjustment change with their care experiences. We extended this strategy to additional placebo conditions, including hypertension, myocardial infarction, and stroke, among others. We replicated the baseline DID design in an FFS sample as another placebo test, using FFS beneficiaries with ADRD as the negative control group and FFS beneficiaries with other neurological conditions (stroke, paralysis, Parkinson) but not ADRD as the comparison group.

An important concern in DID analyses using repeated cross-sectional data is differential compositional changes in the treatment and/or control groups around the reform. We performed corresponding tests, described in eAppendix 5 in Supplement 1, to mitigate this concern.

All statistical analyses used linear probability models for the DID estimator following existing health policy studies in major clinical journals,^11,12,13,14,15,16,17^ and the level of statistical significance (*P* < .05) is based on 2-sided tests. The analyses were performed using Stata version 19.5 (StataCorp) between January 2025 and December 2025.

## Results

### Sample Characteristics

Among the 5353 MA beneficiaries in our sample (1785 male [33.3%]; 1239 [23.1%] aged 65-74 years; 3127 [58.4%] aged ≥75 years; 840 [15.7%] non-Hispanic Black; 887 [16.6%] Hispanic; 3304 [61.7%] non-Hispanic White), 1629 (30.4%) had been diagnosed with ADRD, while the remaining 3724 (69.6%) had a stroke, paralysis, or Parkinson disease (Table). Of all beneficiaries, 1776 (33.2%) were married with a spouse present. More than one-half attained no more than a high school education (2898 beneficiaries [54.1%]). Approximately 73% (3891 beneficiaries [72.7%]) lived with functional limitations (at least 1 instrumental activity of daily living or activity of daily living) and approximately 88% (4690 beneficiaries [87.6%]) had more than 2 chronic conditions.

**Table.** Summary Statistics for Analytical Sample^a^

Characteristics	Beneficiaries, No. (%)		
	All (N = 5353)	ADRD (n = 1629)	Stroke, paralysis, or Parkinson disease (n = 3724)
Dependent variables^b^			
Any troubles getting needed care			
Yes	536 (10.0)	142 (8.7)	394 (10.6)
No	4803 (90.0)	1482 (91.3)	3321 (89.4)
Any medical financial burden^c^			
Yes	975 (23.4)	235 (19.3)	740 (25.1)
No	3197 (76.6)	985 (80.7)	2212 (74.9)
Satisfaction with specialist access			
No	398 (7.9)	140 (9.2)	258 (7.3)
Yes	4651 (92.1)	1384 (90.8)	3267 (92.7)
Satisfaction with quality of care			
No	371 (7.0)	116 (7.2)	255 (7.0)
Yes	4909 (93.0)	1495 (92.8)	3414 (93.0)
Demographics			
Age group, y			
<65	987 (18.4)	113 (6.9)	874 (23.5)
65-74	1239 (23.1)	224 (13.8)	1015 (27.3)
>75	3127 (58.4)	1292 (79.3)	1835 (49.3)
Race and ethnicity^d^			
Non-Hispanic Black	840 (15.7)	217 (13.3)	623 (16.7)
Hispanic	887 (16.6)	394 (24.2)	493 (13.2)
Non-Hispanic White	3304 (61.7)	920 (56.5)	2384 (64.0)
Other	322 (6.0)	98 (6.0)	224 (6.0)
Sex			
Male	1783 (33.3)	443 (27.2)	1341 (36.0)
Female	3570 (66.7)	1186 (72.8)	2383 (64.0)
Marital status			
Not married	2793 (52.2)	892 (54.8)	1901 (51.0)
Married	1776 (33.2)	521 (32.0)	1255 (33.7)
Missing	784 (14.6)	216 (13.3)	568 (15.3)
Education level			
≤High school or less	2898 (54.1)	991 (60.8)	1907 (51.2)
>High school	1629 (30.4)	401 (24.6)	1228 (33.0)
Missing	826 (15.4)	237 (14.5)	589 (15.8)
Respondent status			
Self-respondent	3923 (73.3)	635 (39.0)	3288 (88.3)
Proxy respondent	1430 (26.7)	994 (61.0)	436 (11.7)
Household size			
1	1669 (31.2)	373 (22.9)	1296 (34.8)
2	2318 (43.3)	749 (46.0)	1569 (42.1)
≥3	1366 (25.5)	507 (31.1)	859 (23.1)
Health conditions			
Body mass index^e^			
Underweight or healthy (<25)	1806 (33.7)	702 (43.1)	1104 (29.6)
Overweight (25-30)	1768 (33.0)	475 (29.2)	1293 (34.7)
Obesity or high-risk obesity (≥30)	1615 (30.2)	379 (23.3)	1236 (33.2)
Missing	164 (3.1)	73 (4.5)	91 (2.4)
IADLs or ADLS			
IADLs or ADLs: 0	1462 (27.3)	212 (13.0)	1250 (33.6)
IADLs: 1	926 (17.3)	348 (21.4)	578 (15.5)
ADLs: 1-2	1516 (28.3)	410 (25.2)	1106 (29.7)
ADLs: 3-4	774 (14.5)	296 (18.2)	478 (12.8)
ADLs: 5-6	675 (12.6)	363 (22.3)	312 (8.4)
Chronic conditions (except ADRD)			
0	39 (0.7)	39 (2.4)	0
1	208 (3.9)	124 (7.6)	84 (2.3)
2	416 (7.8)	182 (11.2)	234 (6.3)
3-4	1621 (30.3)	520 (31.9)	1101 (29.6)
5-6	1722 (32.2)	452 (27.7)	1270 (34.1)
7-10	1226 (22.9)	281 (17.2)	945 (25.4)
≥11	121 (2.3)	31 (1.9)	90 (2.4)

Abbreviations: ADL, activity of daily living; ADRD, Alzheimer disease and related dementias; IADL, instrumental activity of daily living.

^a^ The working sample consists of Medicare Advantage beneficiaries from 2015 to 2022 Medicare Current Beneficiary Survey. Beneficiaries with only partial enrollment in a Medicare Advantage plan during the past year at the time of the survey and veterans were excluded.

^b^ For dependent variables, the number of observations varies due to missing values. For each dependent variable, the percentage was calculated with respect to the number of observations with nonmissing values.

^c^ For any medical financial burden, it was not available in 2015 and 2016 Medicare Current Beneficiary Survey data.

^d^ Race and ethnicity were self-reported by beneficiaries. The other category includes all racial groups other than non-Hispanic White, non-Hispanic Black, or Hispanic.

^e^ Calculated as weight in kilograms divided by height in meters squared.

The sample size varied across care experience outcomes due to missing values in the corresponding measures. Of all beneficiaries, 536 (10.0%) reported having any trouble accessing needed care, while 975 (23.4%) reported any medical financial burdens. A significant majority (more than 90%) expressed satisfaction with specialist access (4651 beneficiaries [92.1%]) or the quality of care (4909 beneficiaries [93.0%]). Compared with MA beneficiaries without ADRD, those with ADRD reported lower rates of difficulty accessing care (142 beneficiaries [8.7%] vs 394 beneficiaries [10.6%]) and medical financial burden (235 beneficiaries [19.3%] vs 740 beneficiaries [25.1%]), but slightly lower rates of satisfaction with specialist access (1384 beneficiaries [90.8%] vs 3267 [92.7%]) and care quality (1495 beneficiaries [92.8%] vs 3414 beneficiaries [93.0%]).

**Figure 2.**
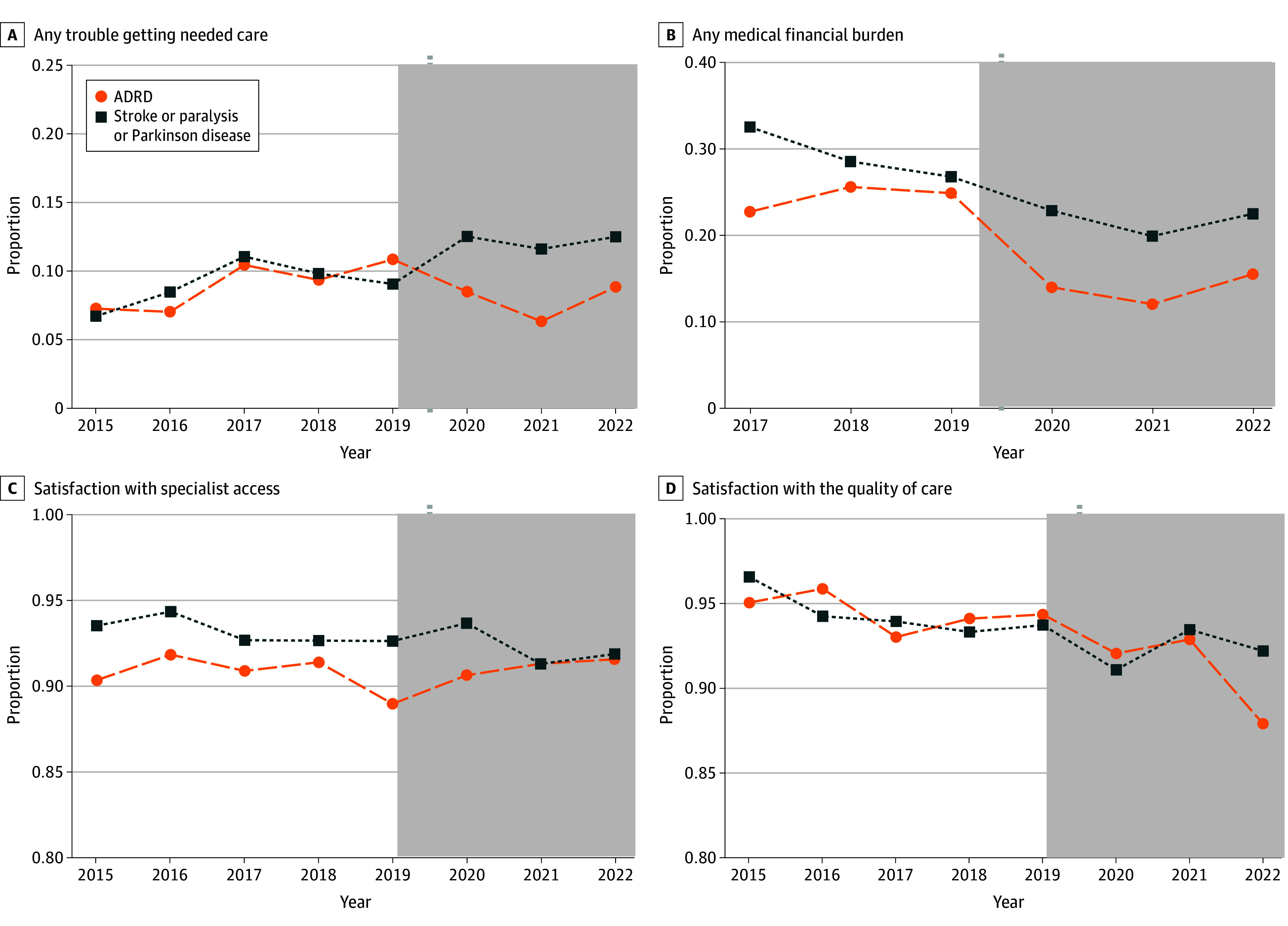
Line Graphs of Pattern of Care Experiences Over Time The working sample consists of Medicare Advantage beneficiaries from the 2015 to 2022 Medicare Current Beneficiary Survey. Beneficiaries with only partial enrollment in a Medicare Advantage plan during the past year at the time of the survey and veterans were excluded. ADRD indicates Alzheimer disease and related dementias.

Figure 2 exhibits trends in each care experience outcome in our treatment and control groups, from 2015 to 2022. Since 2020, a notable decline was observed in the proportion of MA beneficiaries with ADRD reporting any barriers to accessing needed health care (Figure 2A) and any medical financial burdens (Figure 2B) compared with the control group. However, the differences in satisfaction with specialist access (Figure 2C) or the quality of care (Figure 2D) between the 2 groups remained relatively small until 2022. eTable 1 and eTable 2 in Supplement 1 summarize the average outcomes by treatment status and over time. We reported summary statistics for each sample used in robustness and placebo tests in eTables 3 to 19 in Supplement 1.

### MA Risk Adjustment Model Change and Care Experiences

**Figure 3.**
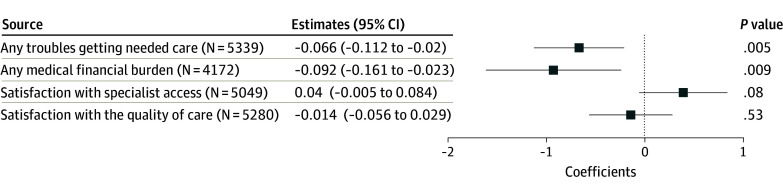
Dot Plot of the Association of the Inclusion of Alzheimer Disease and Related Dementias Hierarchical Condition Categories in Payment Model and Care Experiences The working sample consists of Medicare Advantage beneficiaries from the 2015 to 2022 Medicare Current Beneficiary Survey. Beneficiaries with only partial enrollment in a Medicare Advantage plan during the past year at the time of the survey and veterans were excluded. The plotted estimates represent the coefficients for the interaction term between the treatment indicator and the post indicator from a difference-in-differences estimation, with the dependent variable indicated on the y-axis. In each difference-in-differences estimation, the treatment group consists of Medicare Advantage beneficiaries with Alzheimer disease and related dementias, and the control group consists of Medicare Advantage beneficiaries without Alzheimer disease and related dementias but with stroke or brain hemorrhage, complete or partial paralysis, or Parkinson disease. The post indicator takes the value of 1 if 2020 or afterwards, and 0 otherwise. The associated 95% CIs are plotted. Robust standard errors are applied.

Figure 3 shows improved care experiences following the inclusion of ADRD in the risk-adjusted payment model. The revised risk-adjusted payment model was associated with a 6.62 percentage-point decrease in reporting any troubles accessing needed care (β = 0.06; 95% CI, −0.11 to −0.02; *P* = .005), and a 9.22 percentage-point decrease in reporting any medical financial burdens (β = −0.09; 95% CI, −0.16 to −0.02; *P* = .009) among MA beneficiaries with ADRD. The revised payment model was not associated with satisfaction with improved specialist access (β = 0.40; 95% CI, −0.01 to 0.08; *P* = .08). No significant association was observed for satisfaction with the quality of care (β =  −0.01; 95% CI, −0.06 to 0.03; *P* = .53).

**Figure 4.**
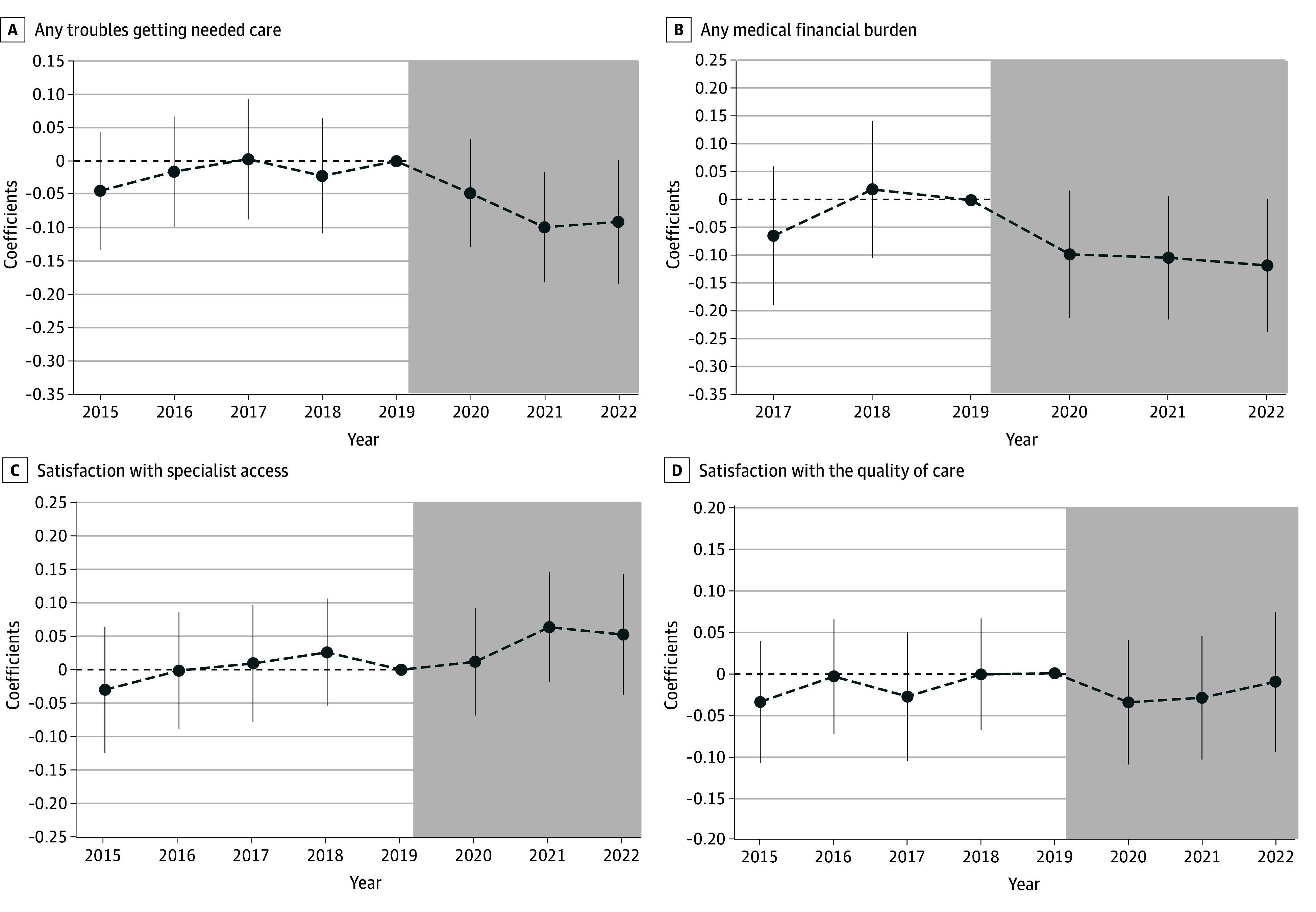
Line Graph of the Dynamic Association of the Inclusion of Alzheimer Disease and Related Dementias Hierarchical Condition Categories in Payment Model and Care Experiences The working sample consists of Medicare Advantage beneficiaries from 2015-2022 Medicare Current Beneficiary Surveys. Beneficiaries with only partial enrollment in a Medicare Advantage plan during the past year at the time of the survey and veterans were excluded. Each figure presents results from an event study model, with the dependent variable specified. The plotted estimates represent the coefficients for the interaction term between treated indicator and year indicators. In each estimation, the treatment group consists of Medicare Advantage beneficiaries with Alzheimer Disease and Related Dementias, and the control group consists of Medicare Advantage beneficiaries without Alzheimer Disease and Related Dementias but with stroke or brain hemorrhage, complete or partial paralysis, or Parkinson disease. The associated 95% CIs are also plotted. Robust standard errors are applied.

Figure 4 displays the dynamic association from 2015 to 2022 using the event study analysis. The negative coefficients and the steady downturn pattern in the period after 2019 reassured that the new risk adjustment model in 2020 was associated with a discernible reduction in troubles in getting needed care (2020: β = −0.05; 95% CI, −0.13 to 0.03; 2021: β = −0.10; 95% CI, −0.18 to −0.02; 2022: β = −0.09; 95% CI, −0.18 to 0.00) (Figure 4A and eTable 20 in Supplement 1) and alleviated financial stress in paying medical bills (2020: β = −0.10; 95% CI, −0.21 to 0.02; 2021: β = −0.10; 95% CI, −0.21 to 0.01; 2022: β = −0.12; 95% CI, −0.24 to 0.00) among MA beneficiaries with ADRD (Figure 4B and eTable 20 in Supplement 1). Moreover, the stability and insignificance of the coefficients preceding the revised payment model in Figure4A-B, being negative or hovering near 0 lend credibility to the underlying parallel-trends assumption for our DID model. The positive association of the 2020 revised payment model and beneficiaries’ satisfaction with specialist access was relatively small in magnitude in 2020 and the subsequent years, although the magnitude of the association showed an increasing trend over time (Figure 4C). Conversely, the revised payment model demonstrated no significant correlation with beneficiaries’ satisfaction regarding the quality of care in any single year from 2015 to 2022, further reassuring the null association depicted in Figure 3.

We performed multiple robustness tests. To address self-reporting bias among MA beneficiaries with ADRD, we excluded self-respondents with ADRD; the estimates were unchanged (eFigure 1 in Supplement 1). Our results were robust to the exclusion of MA beneficiaries younger than 65 years (eFigure 2 in Supplement 1), the adjustment of dual-eligible status in covariates (eFigure 3 in Supplement 1), and the alternative standard error calculations using balanced repeated replications (eFigure 4 in Supplement 1). To address bias from plan-switch due to risk adjustment,^2,8,10^ we restricted the sample to beneficiaries continuously enrolled in MA plans for at least 3 years, yielding consistent results (eFigure 5 in Supplement 1). Including beneficiaries in MA for less than 1 year did not alter our results (eFigure 6 in Supplement 1). Results were robust to alternative control group definitions, including excluding beneficiaries with PD and/or stroke (eFigure 7 in Supplement 1) and using a broader set of non-ADRD conditions as controls (eFigure 8 in Supplement 1).

In eFigure 9 in Supplement 1, we reestimated associations using alternative negative control groups—beneficiaries without ADRD but with one other condition such as stroke or brain hemorrhage, hypertension, myocardial infarction, cancer, or osteoarthritis. Most coefficients were near 0 and statistically insignificant, indicating no spurious associations of the revised payment model with care experience measures. In eFigure 10 in Supplement 1, we compared FFS beneficiaries with ADRD to FFS beneficiaries without ADRD but with stroke, paralysis, or Parkinson disease, finding no significant associations. These placebo results suggest that our main findings were unlikely due to unobserved confounding differences across diseases.

Stratification analyses by beneficiaries’ characteristics are presented in eFigure 11 in Supplement 1. Among beneficiaries with incomes above 200% of the federal poverty level, the revised payment model was significantly associated with reduced trouble getting needed care (β = −0.10; 95% CI, −0.19 to 0.00), while those below 200% of the federal poverty level experienced reductions in both trouble getting needed care (β = −0.08; 95% CI, −0.15 to −0.02) and financial burdens (β = −0.13; 95% CI, −0.22 to −0.04). Beneficiaries who self-reported a race or ethnicity other than non-Hispanic White had a greater reduction in financial burden (β = −0.14; 95% CI, −0.26 to −0.02), whereas non-Hispanic White beneficiaries showed a modest reduction in trouble getting needed care (β = −0.06; 95% CI, −0.11 to 0.00).

By educational attainment, beneficiaries with above a high school degree experienced significant reductions in trouble accessing care (β = −0.10; 95% CI, −0.20 to −0.01) and financial burdens (β = −0.14; 95% CI, −0.28 to 0.00), whereas no significant associations were detected among those with lower education. Lastly, beneficiaries residing in metropolitan areas experienced a notable reduction in trouble getting needed care (β = −0.07; 95% CI, −0.11 to −0.02) and greater satisfaction with specialist access (β = 0.06; 95% CI, 0.01 to 0.11), while no significant associations were observed for nonmetropolitan residents, although the direction of estimates suggest potential benefits. Results in eFigure 12 and eFigure 13 in Supplement 1 show no broad, systematic compositional change around 2019 to 2020, and no consistent patterns of compositional change after 2020 for most beneficiary characteristics, albeit a relative increase in Hispanic share and married share in the treatment group after 2020.

## Discussion

Failing to account for complexity of high-need populations, the risk-adjusted payment model may underreimburse the high costs associated with managing these conditions,^3,5,9,18^ rendering financial disincentives for plans to invest in comprehensive care for affected beneficiaries, for instance, dropping high-cost beneficiaries,^3,18^ providing a narrow provider network,^19,20^ and requiring prior authorization.^21,22^ Consequently, existing studies using data prior to 2020 have found large health gaps between beneficiaries with ADRD in MA and FFS.^6,7,23^ They find that compared to FFS, the diagnosed dementia rates in MA are notably lower,^24^ suggesting a potential underdiagnosis of ADRD in MA. This underdiagnosis causes further delays in access to appropriate interventions, exacerbating gaps in care quality and outcomes. Moreover, beneficiaries with ADRD enrolled in MA have a substantially high rate of leaving MA and joining FFS, especially for those experiencing a great demand for postacute hospitalization, home care, and long-term nursing home care in preceding years.^2,3,4,5,7^ High-cost MA beneficiaries, including patients with ADRD, also have a larger chance of entering into low-quality nursing homes compared to their FFS counterparts.^23^

Since 2004, CMS has periodically revised the HCC risk-adjustment model to improve payment fairness and sustainability. Although previous studies show that risk-adjusted payments can manage overall medical costs,^18,25,26^ less is known about how a condition-specific change in an HCC-based risk adjustment model, especially for care-intensive conditions, incentivizes appropriate care delivery.

This cross-sectional study found that the 2020 reintroduction of ADRD HCC into the payment model was associated with improved care access and reduced financial strain among MA beneficiaries with ADRD. These improvements suggest that updating the risk adjustment model to better reflect the costs of chronic and complex conditions can help realign MA plan incentives with the needs of vulnerable populations. These findings provide a timely assessment of this condition-specific risk adjustment change and inform ongoing refinement of risk-adjusted payment models for CMS.

Our study also identifies several areas of concern. For instance, the improvements in specialist access and care quality were limited after the reintroduction of ADRD into risk adjustment, and benefits were more pronounced among urban and highly educated populations than among rural or less-educated groups. These gaps indicate that while the policy change represents progress, it does not fully overcome systemic barriers to high-quality and comprehensive care for all beneficiaries with ADRD. Policymakers should consider complementary strategies—such as enhanced care coordination, expanded payer networks, and ADRD-specific quality metrics (eg, caregiver support and dementia care training)—to further incentivize high-quality care, specifically for beneficiaries in underserved contexts.

We note several caveats when interpreting our results. First, existing studies have shown an increase in MA enrollment^27^ or an increasing rate of dementia diagnoses in MA beneficiaries who are Hispanic or non-Hispanic Black, dual-eligible, or received low-income subsidies following the 2020 payment model change.^8,10^ Because these groups are more likely to face access and payment challenges, their increased representation in the post-2020 treatment group may attenuate our estimates. Our compositional tests suggest that the relative increase in Hispanic share likely attenuates our estimated improvements in care experiences, while the increase in married share may lead to overestimation.

Moreover, caution should be warranted when interpreting care experience measures. First, the self-reported measures may be subject to response bias. Second, perceived access and affordability do not directly measure the amount, type, or appropriateness of care received; thus, findings should be interpreted as changes in patient experiences rather than definitive changes in utilization or clinical outcomes. Patient experience measures nevertheless have important policy relevance and are widely used in Medicare research and CMS quality monitoring.^6,28,29,30^

We acknowledge the need to monitor the unintended consequences of risk adjustment reforms. It is essential to ensure that this adjustment does not inadvertently lead to cost-shifting or restricted benefits in other areas. Policymakers should consider periodic reviews of risk adjustment models to avoid up-coding and overdiagnosis,^8,10,26^ ensuring a balance of financial sustainability and equity in care delivery.

### Limitations

The MCBS uses a rotating panel design, where each beneficiary can participate for up to 4 years. Our analysis relies on MCBS PUF, a cross-sectional dataset, which does not permit longitudinal linkage of the same individuals across years. Given this data constraint, this study has several limitations. First, we could not directly observe enrollment and diagnosis timing in MCBS PUF, which limited our ability to address the policy-induced compositional changes in the treatment group. Second, because MCBS PUF lacks comprehensive claims and encounter linkages, we could not assess whether reported improvements correspond to changes in objective utilization; future studies using linked claims could evaluate changes in realized care and spending. Third, our ADRD and other conditions measures were based on self-reported physician diagnosis rather than claims data, which may have introduced misclassification and likely attenuated associations toward the null. Fourth, our estimates represent average associations across beneficiaries with heterogeneous disease severity. Fifth, we did not account for the potential cost of overdiagnosis, a possible consequence of the risk-adjusted payment model change.^8^ As such, our study may not fully capture the broader impacts of risk adjustment on care access, quality, and overall welfare.

## Conclusions

This cross-sectional study underscores the value of refining risk adjustment models to better capture clinical complexities of the growing aging population. Expanding the scope of risk adjustment to consider social determinants of health, such as caregiver burden, socioeconomic status, or access to community resources, may help reduce disparities and promote more equitable care.

## Notes

Supplement 1. eAppendix 1. Difference-in-Differences Model eAppendix 2. Event Study Model eAppendix 3. Robustness Checks eAppendix 4. Placebo Test eAppendix 5. Compositional Change Test eTable 1. Summary of Care Experiences by Treatment and Over Time eTable 2. Summary of Care Experiences by Treatment eTable 3. Summary Statistics: Using All Non-ADRD Conditions as Control Group eTable 4. Summary Statistics: Negative Control Group: Stroke/Brain Hemorrhage (non-ADRD), Comparison Group: Non Stroke/Brain Hemorrhage (Non ADRD) eTable 5. Summary Statistics: Negative Control Group: Complete/Partial Paralysis (non-ADRD), Comparison Group: Non Complete/Partial Paralysis (Non ADRD) eTable 6. Summary Statistics: Negative Control Group: Hardening of Arteries (Non-ADRD), Comparison Group: Non Hardening of Arteries (Non ADRD) eTable 7. Summary Statistics: Negative Control Group: Hypertension/High BP (Non-ADRD), Comparison Group: Non Hypertension/High BP (Non ADRD) eTable 8. Summary Statistics: Negative Control Group: MI/Heart Attack (Non-ADRD), Comparison Group: Non MI/heart attack (Non ADRD) eTable 9. Summary Statistics: Negative Control Group: Angina Pectoris/CHD (Non-ADRD), Comparison Group: Non Angina Pectoris/CHD (Non ADRD) eTable 10. Summary Statistics: Negative Control Group: Congestive Heart Failure (Non-ADRD), Comparison Group: Non Congestive Heart Failure (Non ADRD) eTable 11. Summary Statistics: Negative Control Group: Other Heart Cond, eg Valve/Rhythm (non-ADRD), Comparison Group: Non Other Heart Cond, eg Valve/Rhythm (non ADRD) eTable 12. Summary Statistics: Negative Control Group: High Cholesterol (Non-ADRD), Comparison Group: Non High Cholesterol (Non ADRD) eTable 13. Summary Statistics: Negative Control Group: Cancer (Non-ADRD), Comparison Group: Non Cancer (Non ADRD) eTable 14. Summary Statistics: Negative Control Group: Osteoarthritis/Arthritis (Non-ADRD), Comparison Group: Non Osteoarthritis/Arthritis (Non ADRD) eTable 15. Summary Statistics: Negative Control Group: Osteoporosis/Soft Bones (Non-ADRD), Comparison Group: Non Osteoporosis/Soft Bones (Non ADRD) eTable 16. Summary Statistics: Negative Control Group: Broken Hip (Non-ADRD), Comparison Group: Non Broken Hip (Non ADRD) eTable 17. Summary Statistics: Negative Control Group: Emphysema/Asthma/COPD (Non-ADRD), Comparison Group: Non Emphysema/Asthma/COPD (Non ADRD) eTable 18. Summary Statistics: Negative Control Group: Diabetes/High Blood Sugar (Non-ADRD), Comparison Group: Non Diabetes/High Blood Sugar (Non ADRD) eTable 19. Summary Statistics: Using FFS Beneficiaries eTable 20. Dynamic Association Between the Inclusion of ADRD HCCs in Payment Model and Care Experiences (Event Study) eFigure 1. Exclude MA ADRD Beneficiaries With Self-Response eFigure 2. Exclude MA Beneficiaries Under 65 eFigure 3: Adjust for Dual-Eligible Status eFigure 4. Using Balanced Repeated Replications Method eFigure 5. Exclude Beneficiaries in Current MA Plan for Less Than 3 Years eFigure 6. Include Partially Covered MA Beneficiaries eFigure 7. Sensitivity Test to Alternative Control Group eFigure 8. Using MA Beneficiaries With All Non-ADRD Conditions as Control Group eFigure 9. Placebo Tests Using Other Conditions as Negative Control Group eFigure 10. Placebo Tests Using FFS Beneficiaries eFigure 11. Stratification Analysis eFigure 12. Test for Compositional Change eFigure 13. Test for Compositional Change-Event Study

Supplement 2. Data Sharing Statement
