# Supplementary material for: Risk Adjustment for Alzheimer Disease and Related Dementias in Medicare Advantage and Health Care Experiences
Source: JAMA Netw Open. 2026 Mar 13;9(3):e261796. doi: 10.1001/jamanetworkopen.2026.1796 (PMC12988443; doi:10.1001/jamanetworkopen.2026.1796)
Supplement: Supplement 1. — eAppendix 1. Difference-in-Differences Model eAppendix 2. Event Study Model eAppendix 3. Robustness Checks eAppendix 4. Placebo Test eAppendix 5. Compositional Change Test eTable 1. Summary of Care Experiences by Treatment and Over Time eTable 2. Summary of Care Experiences by Treatment eTable 3. Summary Statistics: Using All Non-ADRD Conditions as Control Group eTable 4. Summary Statistics: Negative Control Group: Stroke/Brain Hemorrhage (non-ADRD), Comparison Group: Non Stroke/Brain Hemorrhage (Non ADRD) eTable 5. Summary Statistics: Negative Control Group: Complete/Partial Paralysis (non-ADRD), Comparison Group: Non Complete/Partial Paralysis (Non ADRD) eTable 6. Summary Statistics: Negative Control Group: Hardening of Arteries (Non-ADRD), Comparison Group: Non Hardening of Arteries (Non ADRD) eTable 7. Summary Statistics: Negative Control Group: Hypertension/High BP (Non-ADRD), Comparison Group: Non Hypertension/High BP (Non ADRD) eTable 8. Summary Statistics: Negative Control Group: MI/Heart Attack (Non-ADRD), Comparison Group: Non MI/heart attack (Non ADRD) eTable 9. Summary Statistics: Negative Control Group: Angina Pectoris/CHD (Non-ADRD), Comparison Group: Non Angina Pectoris/CHD (Non ADRD) eTable 10. Summary Statistics: Negative Control Group: Congestive Heart Failure (Non-ADRD), Comparison Group: Non Congestive Heart Failure (Non ADRD) eTable 11. Summary Statistics: Negative Control Group: Other Heart Cond, eg Valve/Rhythm (non-ADRD), Comparison Group: Non Other Heart Cond, eg Valve/Rhythm (non ADRD) eTable 12. Summary Statistics: Negative Control Group: High Cholesterol (Non-ADRD), Comparison Group: Non High Cholesterol (Non ADRD) eTable 13. Summary Statistics: Negative Control Group: Cancer (Non-ADRD), Comparison Group: Non Cancer (Non ADRD) eTable 14. Summary Statistics: Negative Control Group: Osteoarthritis/Arthritis (Non-ADRD), Comparison Group: Non Osteoarthritis/Arthritis (Non ADRD) eTable 15. Summary Statistics: Negative Control Group: Osteoporos [file jamanetwopen-e261796-s001.pdf]

# Supplemental Online Content

Fu W, Qian Y, Karimi S, Zarei H, Chen X. Risk adjustment for Alzheimer disease and related dementias in Medicare Advantage and health care experiences. *JAMA Netw. Open*. 2026;9(3):e261796. doi:10.1001/jamanetworkopen.2026.1796

**eAppendix 1.** Difference-in-Differences Model

**eAppendix 2.** Event Study Model

**eAppendix 3.** Robustness Checks

**eAppendix 4.** Placebo Test

**eAppendix 5.** Compositional Change Test

**eTable 1.** Summary of Care Experiences by Treatment and Over Time

**eTable 2.** Summary of Care Experiences by Treatment

**eTable 3.** Summary Statistics: Using All Non-ADRD Conditions as Control Group

**eTable 4.** Summary Statistics: Negative Control Group: Stroke/Brain Hemorrhage (non-ADRD), Comparison Group: Non Stroke/Brain Hemorrhage (Non ADRD)

**eTable 5.** Summary Statistics: Negative Control Group: Complete/Partial Paralysis (non-ADRD), Comparison Group: Non Complete/Partial Paralysis (Non ADRD)

**eTable 6.** Summary Statistics: Negative Control Group: Hardening of Arteries (Non-ADRD), Comparison Group: Non Hardening of Arteries (Non ADRD)

**eTable 7.** Summary Statistics: Negative Control Group: Hypertension/High BP (Non-ADRD), Comparison Group: Non Hypertension/High BP (Non ADRD)

**eTable 8.** Summary Statistics: Negative Control Group: MI/Heart Attack (Non-ADRD), Comparison Group: Non MI/heart attack (Non ADRD)

**eTable 9.** Summary Statistics: Negative Control Group: Angina Pectoris/CHD (Non-ADRD), Comparison Group: Non Angina Pectoris/CHD (Non ADRD)

**eTable 10.** Summary Statistics: Negative Control Group: Congestive Heart Failure (Non-ADRD), Comparison Group: Non Congestive Heart Failure (Non ADRD)

**eTable 11.** Summary Statistics: Negative Control Group: Other Heart Cond, eg Valve/Rhythm (non-ADRD), Comparison Group: Non Other Heart Cond, eg Valve/Rhythm (non ADRD)

**eTable 12.** Summary Statistics: Negative Control Group: High Cholesterol (Non-ADRD), Comparison Group: Non High Cholesterol (Non ADRD)

**eTable 13.** Summary Statistics: Negative Control Group: Cancer (Non-ADRD), Comparison Group: Non Cancer (Non ADRD)

© 2026 Fu W et al. *JAMA Netw. Open*.

**eTable 14.** Summary Statistics: Negative Control Group: Osteoarthritis/Arthritis (Non-ADRD), Comparison Group: Non Osteoarthritis/Arthritis (Non ADRD)

**eTable 15.** Summary Statistics: Negative Control Group: Osteoporosis/Soft Bones (Non-ADRD), Comparison Group: Non Osteoporosis/Soft Bones (Non ADRD)

**eTable 16.** Summary Statistics: Negative Control Group: Broken Hip (Non-ADRD), Comparison Group: Non Broken Hip (Non ADRD)

**eTable 17.** Summary Statistics: Negative Control Group: Emphysema/Asthma/COPD (Non-ADRD), Comparison Group: Non Emphysema/Asthma/COPD (Non ADRD)

**eTable 18.** Summary Statistics: Negative Control Group: Diabetes/High Blood Sugar (Non-ADRD), Comparison Group: Non Diabetes/High Blood Sugar (Non ADRD)

**eTable 19.** Summary Statistics: Using FFS Beneficiaries

**eTable 20.** Dynamic Association Between the Inclusion of ADRD HCCs in Payment Model and Care Experiences (Event Study)

**eFigure 1.** Exclude MA ADRD Beneficiaries With Self-Response

**eFigure 2.** Exclude MA Beneficiaries Under 65

**eFigure 3:** Adjust for Dual-Eligible Status

**eFigure 4.** Using Balanced Repeated Replications Method

**eFigure 5.** Exclude Beneficiaries in Current MA Plan for Less Than 3 Years

**eFigure 6.** Include Partially Covered MA Beneficiaries

**eFigure 7.** Sensitivity Test to Alternative Control Group

**eFigure 8.** Using MA Beneficiaries With All Non-ADRD Conditions as Control Group

**eFigure 9.** Placebo Tests Using Other Conditions as Negative Control Group

**eFigure 10.** Placebo Tests Using FFS Beneficiaries

**eFigure 11.** Stratification Analysis

**eFigure 12.** Test for Compositional Change

**eFigure 13.** Test for Compositional Change-Event Study

This supplemental material has been provided by the authors to give readers additional information about their work.

© 2026 Fu W et al. *JAMA Netw. Open*.

## eAppendix 1: Difference-in-Differences Model

We employ a DID model to exploit the inclusion of ADRD HCCs in the risk adjustment model for MA plans in 2020 as a quasi-natural experiment. This model compares care experiences between a treatment and a control group before and after the 2020 policy change.

The treated group is defined as MA enrollees who reported having ever been told they have symptoms of ADRD. To construct a comparable control group, we select MA enrollees who: (1) had never been diagnosed with ADRD, and (2) had been diagnosed with conditions such as stroke or brain hemorrhage, complete or partial paralysis, or Parkinson's disease. The selection criteria are grounded in the rationale that patients with these conditions are also likely to see neurologists like those with ADRD. This parallel in healthcare pathways allows for a more equitable comparison, ensuring that differences observed can be more confidently attributed to the effects of the ADRD HCCs integration into the risk adjustment model.

Formally, we build a regression model as outlined in Equation (S.1) below.

$$y_{it} = \beta_0 + \beta_1 treated_{it} \times post_t + \beta_2 treated_{it} + \gamma X'_{it} + \alpha_t + \epsilon_{it}, \qquad \text{(S.1)}$$

where $y_{it}$ represents the care experience measure for beneficiary $i$ in year $t$. The binary variable $treated_{it}$ indicates whether individual $i$ is classified as treated, as defined above. The binary variable $post_t$ takes the value of one for the years following the policy change (2020 and later), and zero otherwise. In a DID framework, the coefficient of the interaction term $treated_{it} \times post_t$, $\beta_1$, captures the association between the inclusion of ADRD HCCs in the risk adjusted payment model and the care experience of MA beneficiaries with ADRD. A positive $\beta_1$ implies a position association. We apply robust standard errors in the analysis.

To reassure the estimated $\hat{\beta}_1$ unbiasedly measures the association of interest, rather than confounded by other unobserved factors, a critical assumption is that, in the absence of the payment model change, the evolution of the outcomes of interest for the control and treatment groups would have followed a similar trajectory over time. This assumption, commonly known as the parallel-trends assumption, is fundamental to the validity of the DID design. It enables the use of the control group's outcomes as a reliable counterfactual for the treated group.

A notable threat to the parallel-trends assumption is the possibility that the estimated $\hat{\beta}_1$ may pick up confounding effects from some concurrent events, such as the onset of the COVID-19 pandemic in early 2020. To address this concern, we undertake several methodological adjustments. First, we include year fixed effects ($\alpha_t$) to absorb national-level shocks and contemporary events. Additionally, to account for

potential heterogeneous pandemic impacts across subpopulations, we incorporate several interaction terms in $X'_{it}$. These include:

(1) interactions between demographic covariates and year indicators, where the demographic covariates encompass age categories, marital status, race/ethnicity, sex, educational attainment, and household size.

(2) the interactions between each health measure and each year indicator, where the health measures include number of functional limitations, number of chronic conditions, and BMI categories.

Our model with these interactions provides greater non-parametric flexibility in accounting for potential heterogeneous exposure to the pandemic, thereby enhancing the robustness of our estimates.

Another potential concern is measurement error arising from proxy reporting, as approximately 27% of the study sample comprises proxy respondents (Table 1). To mitigate the effects of such measurement errors, we include interactions between the self-respondent indicator and all previously described covariates, as well as each time indicator. These interactions allow for a more flexible adjustment, accounting for potential heterogeneity in the patterns and accuracy of proxy reporting.

Moreover, we conduct an event study model, detailed in **Supplementary eMthods 2** to provide suggestive evidence for the parallel-trends assumption.

## eAppendix 2: Event Study Model

We conduct an event study analysis using Equation (S.2) to provide suggestive evidence for the parallel-trends assumption underlying Equation (S.1). All variables in Equation (S.2) are defined consistently as those in Equation (S.1).

$$y_{it} = \beta''_0 + \sum_{j=2015, j\neq 2019}^{2022} \zeta_j treated_{it} \times \mathbb{I}\{t=j\} + \beta''_2 treated_{it} + \gamma'' X'_{it} + \alpha_t + \epsilon''_{it} \tag{S.2}$$

In the equation above, $\mathbb{I}\{t=j\}$ $(2015 \leq j \leq 2022, j \neq 2019)$ is an indicator function. For instance, $\mathbb{I}\{t=2020\}$ takes the value of one if year is 2020 and otherwise zero. We interact the treatment group indicator, $treated_{it}$, with each year indicator, $\mathbb{I}\{t=j\}$. The year 2019, the last year before the implementation of the new risk-adjusted payment model in 2020, serves as the reference year. The coefficient $\zeta_j$ measures the association between the 2020 revised risk adjusted payment model with MA beneficiary's care experience in year $j$.

This analysis serves two primary purposes. First, if $\hat{\zeta}_j$ ($j \leq 2018$) is statistically insignificant, it provides suggestive evidence supporting the parallel-trends assumption, meaning that in the absence of the 2020 payment model revision, the evolution of care experience among MA beneficiaries with ADRD would

follow a parallel trajectory to that of the control group after 2019. Second, if $\hat{\zeta}_j$ ( $j \geq 2020$ ) is statistically significant, it indicates the dynamics of the association between the revised payment model and care experience in the post-revision period. In Figure 4, the $\hat{\zeta}_j$s are plotted with their associated 95% CIs.

## eAppendix 3: Robustness Tests

We performed multiple robustness tests. In **eFigure 1**, we excluded self-respondents with ADRD; the estimates were unchanged, showing that our results are less confounded by self-reporting bias among MA beneficiaries with ADRD. Our results are robust to the exclusion of MA beneficiaries under 65 in **eFigure 2**, and the adjustment of dual-eligible status in covariates in **eFigure 3**. Our results are also robust to alternative standard error calculations using balanced repeated replications in **eFigure 4**.

*Robustness Test: Addressing Plan-Switching Behavior*

We are also concerned about the plan-switching between MA and FFS potentially driven by risk adjustment. On one hand, the care-experience questions in MCBS reference experiences over the prior year; limiting to full-year MA enrollees ensures that reported access and affordability correspond to a stable and well-defined MA exposure window, rather than a mix of MA and traditional Medicare plans within the reporting period. Though our baseline sample requires beneficiaries enrolling in current MA plan for at least one full year, as a robustness check in **eFigure 5**, we restricted the sample to beneficiaries continuously enrolled in MA plans for at least 3 years, again yielding consistent results.

One the other hand, enrollment in MA is not random, and prior evidence has suggested an increasing rate of dementia diagnoses in MA beneficiaries who are Hispanic or Black, dual-eligible, or received low-income subsidies following the 2020 payment model change. Thus, restricting the analytic sample to full-year MA enrollees could therefore introduce selection if enrollment is correlated with care experiences, differs by ADRD status, or changes around the 2020 policy implementation. To address this concern, in **eFigure 6**, we expanded our sample by including MA beneficiaries who are enrolling in their current MA plan for less than one year. In this expanded sample, we continue to observe a statistically significant reduction in any trouble getting needed care. Estimates for the remaining outcomes are attenuated and less precisely estimated. This attenuation pattern could be due to heterogeneity in treatment classification brought by the partial-year MA covered beneficiaries. To be more specific, partial-year MA enrollees experiences both MA and FFS plans within the same survey period. Mid-year switching can mechanically change beneficiary's exposure to benefit design, provider networks, cost-sharing, and care management intensity during the survey reference period. Thus, beneficiaries' reported care experience reflects both a mixture of both plan environment. For instance, with partial-year MA, a beneficiary may spend part of the year in MA and part in FFS (or vice versa). These groups are more heterogeneous because of different timing of plan switching, different exposure to MA plans, different reasons for switching, etc. This

heterogeneity likely weakens statistical power and the mapping between policy-relevant MA exposure and patient-reported experiences, which tends to pull our estimates toward the null.

*Sensitivity Test: Alternative Control Group Constructions*

We perform various tests to assess the sensitivity of our results to alternative control group constructions. Among the 3,715 MA beneficiaries in the baseline control group, 47 have Parkinson disease (PD), 1,039 have complete/partial paralysis, and 3,071 have stroke/brain hemorrhage (these categories are not mutually exclusive, so counts do not sum to the total). Conceptually, PD is neurodegenerative and shares some common clinical features with ADRD, However, in our sample PD constitutes a very small share of the control group (47 individuals), making it unlikely to drive baseline differences or the DID estimates.

In practice, we note that MA beneficiaries with stroke are our preferred neurological control group for MA beneficiaries with ADRD for the following reasons. (1) Beneficiaries with stroke/brain hemorrhage represent the largest component of the control group in our analytical sample. (2) Biologically, existing literature has linked cerebrovascular disease and clinical stroke to cognitive impairment and dementia and highlights the shared vascular pathways contributing to later-life cognitive decline between stroke and AD (Snyder et al., 2015; Anderle et al., 2025). Clinically, stroke survivals also have intensive and longitudinal healthcare needs (Hong et al., 2019) and frequently engage in ongoing specialist follow-up and chronic disease management (and often have cognitive sequelae), which makes their access-to-care and care-experience patterns more likely to move with MA network management and referral processes in ways that are comparable to ADRD-related care needs.

To test if our results are sensitive to the composition of our control group, we re-estimated our DID and event study models using alternative control group definitions and report results in **eFigure 7**.

- *Dropping PD from the control group:* Results are substantively unchanged. The DID estimates remain negative and statistically significant for (i) any trouble getting needed care ($\beta = -0.068$, 95% CI −0.113 to −0.022, $p = 0.004$) and (ii) any medical financial burden ($\beta = -0.092$, 95% CI −0.161 to −0.023, $p = 0.0086$). Estimates for satisfaction outcomes are similar in direction and remain statistically indistinguishable from zero (specialist access: $\beta = 0.040$, 95% CI −0.0053 to 0.085, $p = 0.084$; quality of care: $\beta = -0.013$, 95% CI −0.056 to 0.029, $p = 0.544$). This indicates the primary findings are not driven by PD beneficiaries.
- *Stroke/brain hemorrhage control group.* Results remain qualitatively consistent with the main findings. We continue to observe a negative association between the 2020 payment model change and (i) any trouble getting needed care ($\beta = -0.055$, 95% CI −0.102 to −0.009, $p = 0.020$) and (ii) any medical financial burden ($\beta = -0.093$, 95% CI −0.163 to −0.022, $p = 0.0101$), respectively. The associations for satisfaction outcomes remain imprecisely estimated (specialist access: $\beta =$

0.028, 95% CI −0.0183 to 0.074, $p$ = 0.239; quality of care: $\beta$ = −0.025, 95% CI −0.069 to 0.018, $p$ = 0.259). These estimates are close to the baseline results, supporting robustness to using stroke as a cleaner comparison group.

- *Complete/Partial paralysis control group.* Estimates are directionally similar for trouble getting needed care (–0.114; 95% CI, –0.185 to –0.043; p=0.002), but are generally less precise for the other outcomes (e.g., medical financial burden becomes statistically insignificant), likely reflecting the smaller sample size and greater heterogeneity in the paralysis group. We therefore view paralysis-only as a less preferred standalone control group and present it as an additional robustness check rather than the primary control group.

Moreover, we also replicated our analysis using a broader control group. This expanded control group includes MA beneficiaries without ADRD but with a broader set of conditions, not just limited to PD, Stroke/brain hemorrhage, and Complete/Partial paralysis. Our results remain robust in **eFigure 8**.

Overall, these checks indicate that our conclusions—particularly for measures of perceived access barriers and financial burden—are not driven by PD inclusion (which represents a very small fraction of controls) nor by the broader mix of neurological control diagnoses.

## eAppendix 4: Placebo Tests

We also conduct a series of placebo tests as alternative approaches to support the parallel trends assumption and address the measurement errors described above. The design of these placebo tests involves comparing a group of Medicare Advantage beneficiaries who are not expected to be affected by the inclusion of the ADRD HCC in the risk-adjusted payment model with another group of MA beneficiaries who likewise should not be impacted by the new payment model. Formally, we estimate the following DID model specified in Equation (S.3):

$$y_{it} = \beta_0' + \beta_1' negativeControl_{it} \times post_t + \beta_2' negativeControl_{it} + \gamma' X_{it}' + \alpha_t + \epsilon_{it}' \qquad \text{(S.3)}$$

Equation (S.3) mirrors Equation (S.1), with the primary difference being the definition of the negative control group, denoted as $negativeControl_{it}$. For instance, one illustrative placebo test designates a negative control group, consisting of MA enrollees diagnosed with diabetes/high blood sugar but not ADRD, and a comparison group comprising MA enrollees without either diabetes/high blood sugar or ADRD. Since individuals with diabetes and without ADRD are not the primary target of the policy integrating ADRD HCC into the risk adjustment model, we expect to observe a statistically insignificant $\hat{\beta}_1'$ in this placebo test. This would imply that the 2020 revisions to the risk-adjusted payment model are not statistically associated with improved care experiences for non-ADRD MA beneficiaries with diabetes/high blood sugar. Conversely, if we observe a significant $\hat{\beta}_1'$, it suggests that our benchmark analysis using Equation (S.1)

likely picks up confounding bias from concurrent events such as the onset of the COVID-19 pandemic in 2020 or any potential proxy-response bias.

This approach is then extended to a series of placebo tests, with each negative control group focusing on a distinct health condition, including hypertension or high blood pressure, myocardial infarction (MI)/heart attack, stroke or brain hemorrhage, osteoarthritis or soft bones, congestive heart failure, emphysema/asthma/COPD, and complete or partial paralysis. The full list of chronic conditions serving as placebo-treatment groups is displayed along the horizontal axis in **eFigure 9**. In this figure, each coefficient of association, along with its corresponding 95% confidence interval, is derived from the results of a specific placebo test. Most coefficients were near zero and statistically insignificant, indicating no spurious associations between the revised payment model and care experience measures.

We replicated the baseline DID design in an FFS sample as another placebo test, using FFS beneficiaries with ADRD as the negative control group and FFS beneficiaries with other neurological conditions (stroke, paralysis, Parkinson's) but not ADRD as the comparison group. In **eFigure 10**, we compared FFS beneficiaries with ADRD to FFS beneficiaries without ADRD but with stroke, paralysis, or Parkinson's disease, finding no significant associations. These placebo results suggest that our main findings are unlikely to be driven by unobserved confounding differences across diseases.

## eAppendix 5: Compositional Change Test

A central challenge in our study is distinguishing changes attributable to the 2020 payment policy from compositional change in MA beneficiaries with ADRD around the time of policy implementation. For instance, if the 2020 risk-adjusted payment model policy induced people with severe disease burden to switch to MA plans, this compositional change is likely to undermine the interpretation of our estimation.

In direct response to this concern, we have also performed a series of robustness tests on compositional change using the same DID and event study designs as in our primary analysis. The treatment group consisted of MA enrollees with ADRD, while the control group comprised MA enrollees without ADRD but with stroke/brain hemorrhage, complete/partial paralysis, or Parkinson's diseases.

Specifically, we re-estimated our models treating beneficiary demographic and health-status characteristics as outcomes and tested whether they changed differentially over time for MA beneficiaries with ADRD relative to our control group. The list of characteristics we tested includes age group, sex, race/ethnicity, education, marital status, BMI category, IADL/ADL limitations, and number of chronic conditions. We summarized all DID estimates in **eFigure 12**, in which the plotted estimates represent the coefficients for the interaction term between the treatment indicator and the post indicator from a DID estimation, with the dependent variable indicated on the y-axis. We also present dynamic event study

estimates (relative to year 2019) in **eFigure 13**, where the plotted estimates represent the coefficients for the interaction term between treated indicator and year indicators.

***Demographics.*** **eFigure 12** indicates that for most demographic characteristics, including age categories, sex, education, and racial groups (non-Hispanic white, black), the DID estimates are close to zero and statistically indistinguishable from zero with the 95% CI overlapping zero, suggesting limited evidence of broad demographic compositional change in MA beneficiaries with ADRD relative to the control group following the inclusion of ADRD HCC in the payment model. Importantly, the event studies in **eFigure 13** show no anticipatory jump or fall from 2019 to 2020 for these characteristics, directly addressing the reviewer's concern that diagnostic changes may have occurred in the year before the payment model took effect. Moreover, for these characteristics, the post-2020 coefficients are not statistically significant and do not show a consistent pattern indicating a systematic compositional change in our treatment group after the payment model change.

Nevertheless, we do observe statistically significant and positive DID estimates in **eFigure 12** and post-2020 coefficients in event studies in **eFigure 13** for married share and Hispanic share, suggesting that following the inclusion of ADRD HCC in 2020, there is an increase in married and Hispanic population in the treatment group relative to the control group. Except for these two characteristics, overall demographic profile of our treatment group remains broadly stable across dimensions over years. At the end of this response, we will provide a discussion on these increases in married and Hispanic share bias our interpretation.

***Health status and severity proxies.*** For BMI and IADL/ADL limitation categories, the DID estimates in **eFigure 12** are generally small and not statistically significant; **eFigure 13** similarly shows no sharp shift from 2019 to 2020 and no systematic post-2020 discontinuity in these measures. These findings are reassuring because functional limitations (ADL/IADL) are particularly salient proxies for severity in this setting.

For comorbidity burden, proxied by number of chronic conditions, **eFigure 12** shows modest changes in selected bins (i.e, increase in # chronic conditions: 3-4 and decrease in # chronic conditions: 1), but the pattern is not monotonic across the burden distribution and does not indicate a coherent shift toward uniformly "healthier" or "sicker" beneficiaries after 2020. In the event study, the plots in **eFigure 13** do not show a sharp anticipatory change from 2019 to 2020 nor a sudden discontinuity in 2020 for most of the chronic-condition groups. We do notice that for chronic-condition groups such as chronic condition group 2, 5-6, and 7-10, the post-2020 coefficients fluctuate modestly over time and do not follow a particular pattern. We also need to be cautious that there is a gradually increasing pattern for chronic condition groups 3-4 and 11+, and a decreasing pattern for chronic group 1. Taken patterns across all chronic-condition

groups together, the comorbidity results do not jointly support a systematic compositional change of observed health status in our treatment group after 2020.

These empirical diagnostics show no broad, systematic compositional break around 2019–2020, and significant patterns of compositional change post-2020 for majority of individual's characteristics. We report these tests in the revised Supplement and summarize them in the Results. We kindly invite the reviewer to refer to these sections for more details.

***Implications for bias.*** We further discussed how significance changes, particularly in married and Hispanic population affect the bias of our estimation.

First, the relative increase in Hispanic share in our treatment group following the new payment model in 2020 likely attenuate our estimated improvements in care experiences. Hispanic beneficiaries often face greater structural barriers to care (e.g., language, navigation, access constraints). Mechanically, a treated group that becomes more disadvantaged would tend to report worse access/affordability in care delivery post 2020; therefore, this compositional change would tend to attenuate our DID and post-2020 coefficients in event study to zero. Therefore, we are likely to under-estimate the association between improved care experience and the inclusion of ADRD HCC in risk-adjusted payment model in MA.

Second, increase in the married share in our treatment group after 2020 could lead to over-estimation of improvement in care experience. Marriage can proxy for greater informal caregiving and support in navigating care. If the treatment group became more likely to have a spouse post-2020, this would tend to move reported experiences toward better access/satisfaction and lower burden independent of plan behavior, which would bias estimates away from the null.

Lastly, if the treatment group shifted towards higher comorbidity burden after 2020, the expected direction would be attenuated because higher need generally increases the probability of access problems and cost concerns. However, in our analysis, changes across comorbidity bins are not monotonic and do not indicate a uniform movement toward lower or higher burden; accordingly, any compositional bias from comorbidity shifts is likely limited and not consistently directional.

Overall, while some subgroup-specific shifts merit acknowledgment, the absence of a broad, coherent compositional change post 2020 and particularly the lack of a discrete shift from 2019 to 2020 across most measured characteristics reduce our concern that our primary findings are driven by large observed compositional change. We nevertheless treat residual compositional change, particularly in unobserved severity and diagnosis timing, as an important limitation and interpret results as changes in reported care experiences rather than definitive changes in utilization.

© 2026 Fu W et al. *JAMA Netw. Open*.

**eTable 1: Summary of Care Experiences by Treatment and Over Time**

| Year | Any Troubles Getting Needed Care | | Any Medical Financial Burden | | Satisfaction with Specialist Access | | Satisfaction with Quality of Care | |
|---|---|---|---|---|---|---|---|---|
| | Mean | S.D | Mean | S.D | Mean | S.D | Mean | S.D |
| **Panel A: Control Group: MA beneficiaries with Stroke/Paralysis/PD and no ADRD** | | | | | | | | |
| 2015 | 0.067 | 0.251 | NA | NA | 0.935 | 0.247 | 0.963 | 0.188 |
| 2016 | 0.085 | 0.279 | NA | NA | 0.943 | 0.232 | 0.940 | 0.238 |
| 2017 | 0.111 | 0.314 | 0.326 | 0.469 | 0.927 | 0.261 | 0.937 | 0.243 |
| 2018 | 0.099 | 0.298 | 0.286 | 0.452 | 0.926 | 0.262 | 0.931 | 0.254 |
| 2019 | 0.091 | 0.288 | 0.268 | 0.443 | 0.926 | 0.262 | 0.935 | 0.247 |
| 2020 | 0.125 | 0.332 | 0.229 | 0.420 | 0.937 | 0.244 | 0.909 | 0.288 |
| 2021 | 0.116 | 0.321 | 0.199 | 0.400 | 0.913 | 0.282 | 0.932 | 0.252 |
| 2022 | 0.125 | 0.331 | 0.225 | 0.418 | 0.919 | 0.274 | 0.920 | 0.272 |
| **Panel B: Treatment Group: MA beneficiaries with ADRD** | | | | | | | | |
| 2015 | 0.073 | 0.261 | NA | NA | 0.903 | 0.297 | 0.948 | 0.223 |
| 2016 | 0.071 | 0.257 | NA | NA | 0.918 | 0.275 | 0.956 | 0.205 |
| 2017 | 0.105 | 0.307 | 0.227 | 0.420 | 0.909 | 0.289 | 0.928 | 0.259 |
| 2018 | 0.094 | 0.292 | 0.256 | 0.438 | 0.914 | 0.282 | 0.939 | 0.240 |
| 2019 | 0.109 | 0.312 | 0.249 | 0.433 | 0.889 | 0.314 | 0.941 | 0.236 |
| 2020 | 0.085 | 0.280 | 0.139 | 0.347 | 0.906 | 0.292 | 0.918 | 0.275 |
| 2021 | 0.064 | 0.245 | 0.120 | 0.326 | 0.913 | 0.283 | 0.926 | 0.262 |
| 2022 | 0.089 | 0.285 | 0.155 | 0.362 | 0.915 | 0.279 | 0.877 | 0.329 |

Notes: The working sample consists of MA beneficiaries from 2015-2022 MCBS. Beneficiaries with only partial enrollment in an MA plan during the past year at the time of the survey and veteran are excluded.

© 2026 Fu W et al. *JAMA Netw. Open*.

**eTable 2. Summary of Care Experiences by Treatment and Year**

| | Treatment | Control |
|---|---|---|
| **Panel A: Any Troubles Getting Needed Care** | | |
| Before 2020 | 0.093 (0.290) | 0.093 (0.290) |
| 2020 and post | 0.08 (0.271) | 0.122 (0.328) |
| | | |
| **Panel B: Any Medical Finance Burden** | | |
| Before 2020 | 0.244 (0.430) | 0.291 (0.455) |
| 2020 and post | 0.138 (0.345) | 0.217 (0.413) |
| | | |
| **Panel C: Satisfaction with Specialist Access** | | |
| Before 2020 | 0.906 (0.292) | 0.931 (0.254) |
| 2020 and post | 0.911 (0.285) | 0.922 (0.268) |
| | | |
| **Panel D: Satisfaction with Quality of Care** | | |
| Before 2020 | 0.942 (0.235) | 0.939 (0.240) |
| 2020 and post | 0.907 (0.291) | 0.92 (0.271) |

Notes: The working sample consists of MA beneficiaries from 2015-2022 MCBS. Beneficiaries with only partial enrollment in an MA plan during the past year at the time of the survey and veterans are excluded. The standard deviations are reported in parentheses

© 2026 Fu W et al. *JAMA Netw. Open*.

**eTable 3. Summary Statistics: using All non-ADRD Conditions as Control Group**

| | (1) | | | (2) | | | (3) | | |
|---|---|---|---|---|---|---|---|---|---|
| | All | | | ADRD | | | All non-ADRD | | |
| | Mean | SD | N | Mean | SD | N | Mean | SD | N |
| Any Troubles Getting Needed Care | 0.079 | 0.269 | 31608 | 0.087 | 0.283 | 1624 | 0.078 | 0.268 | 29984 |
| Any Medical Financial Burden | 0.187 | 0.390 | 25289 | 0.193 | 0.395 | 1220 | 0.187 | 0.390 | 24069 |
| Satisfaction with Access to Specialist | 0.933 | 0.249 | 29617 | 0.908 | 0.289 | 1524 | 0.935 | 0.247 | 28093 |
| Satisfaction with Quality of Care | 0.951 | 0.217 | 31175 | 0.928 | 0.259 | 1611 | 0.952 | 0.214 | 29564 |
| No ADRD | 0.949 | 0.221 | 31663 | 0.000 | 0.000 | 1629 | 1.000 | 0.000 | 30034 |
| ADRD | 0.051 | 0.221 | 31663 | 1.000 | 0.000 | 1629 | 0.000 | 0.000 | 30034 |
| Age Group <65 | 0.153 | 0.360 | 31663 | 0.069 | 0.254 | 1629 | 0.158 | 0.364 | 30034 |
| Age Group 65 to 75 | 0.342 | 0.474 | 31663 | 0.138 | 0.344 | 1629 | 0.353 | 0.478 | 30034 |
| Age Group 75+ | 0.505 | 0.500 | 31663 | 0.793 | 0.405 | 1629 | 0.490 | 0.500 | 30034 |
| Non-Hispanic white | 0.650 | 0.477 | 31663 | 0.565 | 0.496 | 1629 | 0.655 | 0.475 | 30034 |
| Non-Hispanic black | 0.128 | 0.335 | 31663 | 0.133 | 0.340 | 1629 | 0.128 | 0.334 | 30034 |
| Hispanic | 0.163 | 0.369 | 31663 | 0.242 | 0.428 | 1629 | 0.159 | 0.365 | 30034 |
| Other | 0.058 | 0.234 | 31663 | 0.060 | 0.238 | 1629 | 0.058 | 0.234 | 30034 |
| Male | 0.326 | 0.469 | 31663 | 0.272 | 0.445 | 1629 | 0.329 | 0.470 | 30034 |
| Female | 0.674 | 0.469 | 31663 | 0.728 | 0.445 | 1629 | 0.671 | 0.470 | 30034 |
| Not Married | 0.473 | 0.499 | 31663 | 0.548 | 0.498 | 1629 | 0.469 | 0.499 | 30034 |
| Married | 0.368 | 0.482 | 31663 | 0.320 | 0.467 | 1629 | 0.370 | 0.483 | 30034 |
| Married: missing | 0.159 | 0.366 | 31663 | 0.133 | 0.339 | 1629 | 0.161 | 0.367 | 30034 |
| High school or less | 0.483 | 0.500 | 31663 | 0.608 | 0.488 | 1629 | 0.477 | 0.499 | 30034 |
| High School above | 0.354 | 0.478 | 31663 | 0.246 | 0.431 | 1629 | 0.360 | 0.480 | 30034 |
| Education missing | 0.163 | 0.369 | 31663 | 0.145 | 0.353 | 1629 | 0.164 | 0.370 | 30034 |
| Self-respondent | 0.897 | 0.304 | 31663 | 0.390 | 0.488 | 1629 | 0.925 | 0.264 | 30034 |
| Proxy respondent | 0.103 | 0.304 | 31663 | 0.610 | 0.488 | 1629 | 0.075 | 0.264 | 30034 |
| Household Size: 1 | 0.331 | 0.471 | 31663 | 0.229 | 0.420 | 1629 | 0.337 | 0.473 | 30034 |
| Household Size: 2 | 0.452 | 0.498 | 31663 | 0.460 | 0.499 | 1629 | 0.452 | 0.498 | 30034 |
| Household Size: 3+ | 0.216 | 0.412 | 31663 | 0.311 | 0.463 | 1629 | 0.211 | 0.408 | 30034 |
| Underweight/healthy (<25) | 0.305 | 0.460 | 31663 | 0.431 | 0.495 | 1629 | 0.298 | 0.457 | 30034 |
| Overweight (25-30) | 0.338 | 0.473 | 31663 | 0.292 | 0.455 | 1629 | 0.340 | 0.474 | 30034 |
| Obese/high-risk obese (>=30) | 0.330 | 0.470 | 31663 | 0.233 | 0.423 | 1629 | 0.336 | 0.472 | 30034 |
| BMI missing | 0.027 | 0.163 | 31663 | 0.045 | 0.207 | 1629 | 0.026 | 0.160 | 30034 |
| IADLs or ADLs: 0 | 0.519 | 0.500 | 31663 | 0.130 | 0.337 | 1629 | 0.540 | 0.498 | 30034 |
| IADLs: 1 | 0.157 | 0.364 | 31663 | 0.214 | 0.410 | 1629 | 0.154 | 0.361 | 30034 |
| ADLs: 1-2 | 0.209 | 0.406 | 31663 | 0.252 | 0.434 | 1629 | 0.206 | 0.405 | 30034 |
| ADLs: 3-4 | 0.074 | 0.261 | 31663 | 0.182 | 0.386 | 1629 | 0.068 | 0.252 | 30034 |
| ADLs: 5-6 | 0.041 | 0.199 | 31663 | 0.223 | 0.416 | 1629 | 0.032 | 0.175 | 30034 |
| Chronic Conditions (except ADRD): 0 | 0.001 | 0.035 | 31663 | 0.024 | 0.153 | 1629 | 0.000 | 0.000 | 30034 |
| Chronic Conditions (except ADRD): 1 | 0.112 | 0.315 | 31663 | 0.076 | 0.265 | 1629 | 0.114 | 0.317 | 30034 |
| Chronic Conditions (except ADRD): 2 | 0.173 | 0.378 | 31663 | 0.112 | 0.315 | 1629 | 0.176 | 0.381 | 30034 |

© 2026 Fu W et al. *JAMA Netw. Open*.

| | | | | | | | | | |
|---|---|---|---|---|---|---|---|---|---|
| Chronic Conditions (except ADRD): 3-4 | 0.386 | 0.487 | 31663 | 0.319 | 0.466 | 1629 | 0.390 | 0.488 | 30034 |
| Chronic Conditions (except ADRD): 5-6 | 0.221 | 0.415 | 31663 | 0.277 | 0.448 | 1629 | 0.218 | 0.413 | 30034 |
| Chronic Conditions (except ADRD): 7-10 | 0.101 | 0.302 | 31663 | 0.172 | 0.378 | 1629 | 0.098 | 0.297 | 30034 |
| Chronic Conditions (except ADRD): 11+ | 0.006 | 0.074 | 31663 | 0.019 | 0.137 | 1629 | 0.005 | 0.069 | 30034 |

Notes: The working sample consists of MA beneficiaries from 2015-2022 MCBS. Beneficiaries with only partial enrollment in an MA plan during the past year at the time of the survey and veterans are excluded.

© 2026 Fu W et al. *JAMA Netw. Open*.

**eTable 4. Summary Statistics: Negative Control Group: Stroke/Brain Hemorrhage (non-ADRD), Comparison Group: non Stroke/Brain Hemorrhage (non ADRD)**

| | (1) | | | (2) | | | (3) | | |
|---|---|---|---|---|---|---|---|---|---|
| | Negative Control + Comparison | | | Negative Control | | | Comparison | | |
| | Mean | SD | N | Mean | SD | N | Mean | SD | N |
| Any Troubles Getting Needed Care | 0.077 | 0.267 | 31608 | 0.092 | 0.290 | 3071 | 0.076 | 0.264 | 28537 |
| Any Medical Financial Burden | 0.184 | 0.387 | 25321 | 0.242 | 0.428 | 2472 | 0.177 | 0.382 | 22849 |
| Satisfaction with Access to Specialist | 0.935 | 0.247 | 29416 | 0.931 | 0.253 | 2915 | 0.935 | 0.246 | 26501 |
| Satisfaction with Quality of Care | 0.952 | 0.214 | 31022 | 0.933 | 0.250 | 3040 | 0.954 | 0.209 | 27982 |
| Comparison | 0.903 | 0.296 | 31657 | 0.000 | 0.000 | 3080 | 1.000 | 0.000 | 28577 |
| Negative Control | 0.097 | 0.296 | 31657 | 1.000 | 0.000 | 3080 | 0.000 | 0.000 | 28577 |
| Age Group <65 | 0.169 | 0.374 | 31657 | 0.193 | 0.394 | 3080 | 0.166 | 0.372 | 28577 |
| Age Group 65 to 75 | 0.352 | 0.478 | 31657 | 0.276 | 0.447 | 3080 | 0.360 | 0.480 | 28577 |
| Age Group 75+ | 0.480 | 0.500 | 31657 | 0.532 | 0.499 | 3080 | 0.474 | 0.499 | 28577 |
| Non-Hispanic white | 0.655 | 0.475 | 31657 | 0.636 | 0.481 | 3080 | 0.657 | 0.475 | 28577 |
| Non-Hispanic black | 0.129 | 0.335 | 31657 | 0.182 | 0.386 | 3080 | 0.123 | 0.329 | 28577 |
| Hispanic | 0.158 | 0.365 | 31657 | 0.121 | 0.327 | 3080 | 0.162 | 0.368 | 28577 |
| Other | 0.058 | 0.234 | 31657 | 0.060 | 0.238 | 3080 | 0.058 | 0.234 | 28577 |
| Male | 0.334 | 0.472 | 31657 | 0.344 | 0.475 | 3080 | 0.333 | 0.471 | 28577 |
| Female | 0.666 | 0.472 | 31657 | 0.656 | 0.475 | 3080 | 0.667 | 0.471 | 28577 |
| Not Married | 0.473 | 0.499 | 31657 | 0.507 | 0.500 | 3080 | 0.469 | 0.499 | 28577 |
| Married | 0.367 | 0.482 | 31657 | 0.342 | 0.474 | 3080 | 0.370 | 0.483 | 28577 |
| Married: missing | 0.160 | 0.367 | 31657 | 0.151 | 0.358 | 3080 | 0.161 | 0.368 | 28577 |
| High school or less | 0.477 | 0.499 | 31657 | 0.538 | 0.499 | 3080 | 0.470 | 0.499 | 28577 |
| High School above | 0.360 | 0.480 | 31657 | 0.304 | 0.460 | 3080 | 0.366 | 0.482 | 28577 |
| Education missing | 0.163 | 0.370 | 31657 | 0.158 | 0.365 | 3080 | 0.164 | 0.370 | 28577 |
| Self-respondent | 0.920 | 0.271 | 31657 | 0.878 | 0.327 | 3080 | 0.925 | 0.264 | 28577 |
| Proxy respondent | 0.080 | 0.271 | 31657 | 0.122 | 0.327 | 3080 | 0.075 | 0.264 | 28577 |
| Household Size: 1 | 0.335 | 0.472 | 31657 | 0.351 | 0.477 | 3080 | 0.333 | 0.471 | 28577 |
| Household Size: 2 | 0.450 | 0.498 | 31657 | 0.426 | 0.495 | 3080 | 0.453 | 0.498 | 28577 |
| Household Size: 3+ | 0.215 | 0.411 | 31657 | 0.223 | 0.417 | 3080 | 0.214 | 0.410 | 28577 |
| Underweight/healthy (<25) | 0.304 | 0.460 | 31657 | 0.300 | 0.458 | 3080 | 0.304 | 0.460 | 28577 |
| Overweight (25-30) | 0.340 | 0.474 | 31657 | 0.361 | 0.480 | 3080 | 0.337 | 0.473 | 28577 |
| Obese/high-risk obese (>=30) | 0.329 | 0.470 | 31657 | 0.314 | 0.464 | 3080 | 0.331 | 0.471 | 28577 |
| BMI missing | 0.027 | 0.162 | 31657 | 0.025 | 0.156 | 3080 | 0.027 | 0.163 | 28577 |
| IADLs or ADLs: 0 | 0.547 | 0.498 | 31657 | 0.356 | 0.479 | 3080 | 0.568 | 0.495 | 28577 |
| IADLs: 1 | 0.155 | 0.362 | 31657 | 0.160 | 0.367 | 3080 | 0.155 | 0.362 | 28577 |
| ADLs: 1-2 | 0.201 | 0.401 | 31657 | 0.297 | 0.457 | 3080 | 0.191 | 0.393 | 28577 |
| ADLs: 3-4 | 0.066 | 0.248 | 31657 | 0.118 | 0.322 | 3080 | 0.060 | 0.238 | 28577 |
| ADLs: 5-6 | 0.031 | 0.173 | 31657 | 0.070 | 0.255 | 3080 | 0.027 | 0.162 | 28577 |
| Chronic Conditions (except ADRD): 0 | 0.052 | 0.222 | 31657 | 0.000 | 0.000 | 3080 | 0.058 | 0.233 | 28577 |

© 2026 Fu W et al. *JAMA Netw. Open*.

| | | | | | | | | | |
|---|---|---|---|---|---|---|---|---|---|
| Chronic Conditions (except ADRD): 1 | 0.108 | 0.310 | 31657 | 0.014 | 0.119 | 3080 | 0.118 | 0.322 | 28577 |
| Chronic Conditions (except ADRD): 2 | 0.167 | 0.373 | 31657 | 0.050 | 0.219 | 3080 | 0.179 | 0.384 | 28577 |
| Chronic Conditions (except ADRD): 3-4 | 0.370 | 0.483 | 31657 | 0.287 | 0.453 | 3080 | 0.379 | 0.485 | 28577 |
| Chronic Conditions (except ADRD): 5-6 | 0.207 | 0.405 | 31657 | 0.351 | 0.477 | 3080 | 0.191 | 0.393 | 28577 |
| Chronic Conditions (except ADRD): 7-10 | 0.093 | 0.290 | 31657 | 0.271 | 0.445 | 3080 | 0.073 | 0.261 | 28577 |
| Chronic Conditions (except ADRD): 11+ | 0.005 | 0.068 | 31657 | 0.026 | 0.160 | 3080 | 0.002 | 0.047 | 28577 |

Notes: The working sample consists of MA beneficiaries from 2015-2022 MCBS. Beneficiaries with only partial enrollment in an MA plan during the past year at the time of the survey and veterans are excluded. We also exclude beneficiaries with ADRD.

© 2026 Fu W et al. *JAMA Netw. Open*.

eTable 5. Summary Statistics: Negative Control Group: Complete/Partial Paralysis (non-ADRD), Comparison Group: non Complete/Partial Paralysis (non ADRD)

| | (1) Negative Control + Comparison | | | (2) Negative Control | | | (3) Comparison | | |
|---|---|---|---|---|---|---|---|---|---|
| | Mean | SD | N | Mean | SD | N | Mean | SD | N |
| Any Troubles Getting Needed Care | 0.077 | 0.267 | 31589 | 0.158 | 0.365 | 1037 | 0.074 | 0.263 | 30552 |
| Any Medical Financial Burden | 0.184 | 0.387 | 25309 | 0.298 | 0.458 | 826 | 0.180 | 0.384 | 24483 |
| Satisfaction with Access to Specialist | 0.935 | 0.247 | 29399 | 0.897 | 0.304 | 980 | 0.936 | 0.245 | 28419 |
| Satisfaction with Quality of Care | 0.952 | 0.214 | 31004 | 0.917 | 0.277 | 1018 | 0.953 | 0.211 | 29986 |
| Comparison | 0.967 | 0.178 | 31638 | 0.000 | 0.000 | 1041 | 1.000 | 0.000 | 30597 |
| Negative Control | 0.033 | 0.178 | 31638 | 1.000 | 0.000 | 1041 | 0.000 | 0.000 | 30597 |
| Age Group <65 | 0.168 | 0.374 | 31638 | 0.419 | 0.494 | 1041 | 0.160 | 0.367 | 30597 |
| Age Group 65 to 75 | 0.352 | 0.478 | 31638 | 0.269 | 0.444 | 1041 | 0.355 | 0.478 | 30597 |
| Age Group 75+ | 0.480 | 0.500 | 31638 | 0.312 | 0.464 | 1041 | 0.485 | 0.500 | 30597 |
| Non-Hispanic white | 0.655 | 0.475 | 31638 | 0.652 | 0.476 | 1041 | 0.655 | 0.475 | 30597 |
| Non-Hispanic black | 0.129 | 0.335 | 31638 | 0.112 | 0.316 | 1041 | 0.130 | 0.336 | 30597 |
| Hispanic | 0.158 | 0.365 | 31638 | 0.183 | 0.386 | 1041 | 0.157 | 0.364 | 30597 |
| Other | 0.058 | 0.234 | 31638 | 0.053 | 0.224 | 1041 | 0.058 | 0.235 | 30597 |
| Male | 0.334 | 0.472 | 31638 | 0.404 | 0.491 | 1041 | 0.332 | 0.471 | 30597 |
| Female | 0.666 | 0.472 | 31638 | 0.596 | 0.491 | 1041 | 0.668 | 0.471 | 30597 |
| Not Married | 0.473 | 0.499 | 31638 | 0.533 | 0.499 | 1041 | 0.471 | 0.499 | 30597 |
| Married | 0.367 | 0.482 | 31638 | 0.308 | 0.462 | 1041 | 0.369 | 0.483 | 30597 |
| Married: missing | 0.160 | 0.367 | 31638 | 0.159 | 0.365 | 1041 | 0.160 | 0.367 | 30597 |
| High school or less | 0.477 | 0.499 | 31638 | 0.414 | 0.493 | 1041 | 0.479 | 0.500 | 30597 |
| High School above | 0.360 | 0.480 | 31638 | 0.425 | 0.495 | 1041 | 0.358 | 0.479 | 30597 |
| Education missing | 0.163 | 0.370 | 31638 | 0.161 | 0.368 | 1041 | 0.163 | 0.370 | 30597 |
| Self-respondent | 0.920 | 0.271 | 31638 | 0.886 | 0.318 | 1041 | 0.922 | 0.269 | 30597 |
| Proxy respondent | 0.080 | 0.271 | 31638 | 0.114 | 0.318 | 1041 | 0.078 | 0.269 | 30597 |
| Household Size: 1 | 0.335 | 0.472 | 31638 | 0.330 | 0.471 | 1041 | 0.335 | 0.472 | 30597 |
| Household Size: 2 | 0.450 | 0.498 | 31638 | 0.378 | 0.485 | 1041 | 0.453 | 0.498 | 30597 |
| Household Size: 3+ | 0.215 | 0.411 | 31638 | 0.292 | 0.455 | 1041 | 0.212 | 0.409 | 30597 |
| Underweight/healthy (<25) | 0.304 | 0.460 | 31638 | 0.286 | 0.452 | 1041 | 0.304 | 0.460 | 30597 |
| Overweight (25-30) | 0.340 | 0.474 | 31638 | 0.287 | 0.453 | 1041 | 0.341 | 0.474 | 30597 |
| Obese/high-risk obese (>=30) | 0.329 | 0.470 | 31638 | 0.403 | 0.491 | 1041 | 0.327 | 0.469 | 30597 |
| BMI missing | 0.027 | 0.163 | 31638 | 0.023 | 0.150 | 1041 | 0.027 | 0.163 | 30597 |
| IADLs or ADLs: 0 | 0.547 | 0.498 | 31638 | 0.224 | 0.417 | 1041 | 0.558 | 0.497 | 30597 |
| IADLs: 1 | 0.155 | 0.362 | 31638 | 0.137 | 0.344 | 1041 | 0.156 | 0.363 | 30597 |
| ADLs: 1-2 | 0.201 | 0.401 | 31638 | 0.284 | 0.451 | 1041 | 0.198 | 0.399 | 30597 |
| ADLs: 3-4 | 0.066 | 0.248 | 31638 | 0.197 | 0.398 | 1041 | 0.061 | 0.240 | 30597 |
| ADLs: 5-6 | 0.031 | 0.173 | 31638 | 0.158 | 0.364 | 1041 | 0.027 | 0.161 | 30597 |
| Chronic Conditions (except ADRD): 0 | 0.052 | 0.222 | 31638 | 0.000 | 0.000 | 1041 | 0.054 | 0.226 | 30597 |
| Chronic Conditions (except ADRD): 1 | 0.108 | 0.310 | 31638 | 0.037 | 0.188 | 1041 | 0.110 | 0.313 | 30597 |

| | | | | | | | | | |
|---|---|---|---|---|---|---|---|---|---|
| Chronic Conditions (except ADRD): 2 | 0.167 | 0.373 | 31638 | 0.086 | 0.281 | 1041 | 0.170 | 0.375 | 30597 |
| Chronic Conditions (except ADRD): 3-4 | 0.370 | 0.483 | 31638 | 0.271 | 0.445 | 1041 | 0.373 | 0.484 | 30597 |
| Chronic Conditions (except ADRD): 5-6 | 0.207 | 0.405 | 31638 | 0.327 | 0.469 | 1041 | 0.202 | 0.402 | 30597 |
| Chronic Conditions (except ADRD): 7-10 | 0.092 | 0.290 | 31638 | 0.250 | 0.433 | 1041 | 0.087 | 0.282 | 30597 |
| Chronic Conditions (except ADRD): 11+ | 0.005 | 0.068 | 31638 | 0.030 | 0.170 | 1041 | 0.004 | 0.061 | 30597 |

Notes: The working sample consists of MA beneficiaries from 2015-2022 MCBS. Beneficiaries with only partial enrollment in an MA plan during the past year at the time of the survey and veterans are excluded. We also exclude beneficiaries with ADRD.

© 2026 Fu W et al. *JAMA Netw. Open*.

**eTable 6. Summary Statistics: Negative Control Group: Hardening of arteries (non-ADRD), Comparison Group: non Hardening of arteries (non ADRD)**

| | (1) | | | (2) | | | (3) | | |
|---|---|---|---|---|---|---|---|---|---|
| | Negative Control + Comparison | | | Negative Control | | | Comparison | | |
| | Mean | SD | N | Mean | SD | N | Mean | SD | N |
| Any Troubles Getting Needed Care | 0.077 | 0.267 | 31465 | 0.085 | 0.279 | 2089 | 0.076 | 0.266 | 29376 |
| Any Medical Financial Burden | 0.183 | 0.387 | 25216 | 0.225 | 0.418 | 1645 | 0.180 | 0.385 | 23571 |
| Satisfaction with Access to Specialist | 0.935 | 0.247 | 29288 | 0.931 | 0.253 | 2019 | 0.935 | 0.246 | 27269 |
| Satisfaction with Quality of Care | 0.952 | 0.214 | 30884 | 0.937 | 0.243 | 2076 | 0.953 | 0.212 | 28808 |
| Comparison | 0.933 | 0.249 | 31514 | 0.000 | 0.000 | 2096 | 1.000 | 0.000 | 29418 |
| Negative Control | 0.067 | 0.249 | 31514 | 1.000 | 0.000 | 2096 | 0.000 | 0.000 | 29418 |
| Age Group <65 | 0.168 | 0.374 | 31514 | 0.106 | 0.308 | 2096 | 0.173 | 0.378 | 29418 |
| Age Group 65 to 75 | 0.352 | 0.478 | 31514 | 0.312 | 0.464 | 2096 | 0.355 | 0.479 | 29418 |
| Age Group 75+ | 0.479 | 0.500 | 31514 | 0.582 | 0.493 | 2096 | 0.472 | 0.499 | 29418 |
| Non-Hispanic white | 0.656 | 0.475 | 31514 | 0.669 | 0.471 | 2096 | 0.655 | 0.475 | 29418 |
| Non-Hispanic black | 0.129 | 0.335 | 31514 | 0.081 | 0.272 | 2096 | 0.132 | 0.339 | 29418 |
| Hispanic | 0.158 | 0.364 | 31514 | 0.183 | 0.387 | 2096 | 0.156 | 0.363 | 29418 |
| Other | 0.058 | 0.233 | 31514 | 0.067 | 0.251 | 2096 | 0.057 | 0.232 | 29418 |
| Male | 0.334 | 0.472 | 31514 | 0.415 | 0.493 | 2096 | 0.328 | 0.470 | 29418 |
| Female | 0.666 | 0.472 | 31514 | 0.585 | 0.493 | 2096 | 0.672 | 0.470 | 29418 |
| Not Married | 0.473 | 0.499 | 31514 | 0.469 | 0.499 | 2096 | 0.473 | 0.499 | 29418 |
| Married | 0.367 | 0.482 | 31514 | 0.386 | 0.487 | 2096 | 0.366 | 0.482 | 29418 |
| Married: missing | 0.160 | 0.367 | 31514 | 0.145 | 0.352 | 2096 | 0.161 | 0.368 | 29418 |
| High school or less | 0.477 | 0.499 | 31514 | 0.439 | 0.496 | 2096 | 0.479 | 0.500 | 29418 |
| High School above | 0.360 | 0.480 | 31514 | 0.415 | 0.493 | 2096 | 0.356 | 0.479 | 29418 |
| Education missing | 0.163 | 0.369 | 31514 | 0.146 | 0.354 | 2096 | 0.164 | 0.370 | 29418 |
| Self-respondent | 0.921 | 0.270 | 31514 | 0.912 | 0.283 | 2096 | 0.921 | 0.269 | 29418 |
| Proxy respondent | 0.079 | 0.270 | 31514 | 0.088 | 0.283 | 2096 | 0.079 | 0.269 | 29418 |
| Household Size: 1 | 0.335 | 0.472 | 31514 | 0.317 | 0.466 | 2096 | 0.336 | 0.472 | 29418 |
| Household Size: 2 | 0.450 | 0.498 | 31514 | 0.462 | 0.499 | 2096 | 0.450 | 0.497 | 29418 |
| Household Size: 3+ | 0.215 | 0.411 | 31514 | 0.221 | 0.415 | 2096 | 0.214 | 0.410 | 29418 |
| Underweight/healthy (<25) | 0.304 | 0.460 | 31514 | 0.306 | 0.461 | 2096 | 0.304 | 0.460 | 29418 |
| Overweight (25-30) | 0.340 | 0.474 | 31514 | 0.375 | 0.484 | 2096 | 0.337 | 0.473 | 29418 |
| Obese/high-risk obese (>=30) | 0.330 | 0.470 | 31514 | 0.301 | 0.459 | 2096 | 0.332 | 0.471 | 29418 |
| BMI missing | 0.027 | 0.162 | 31514 | 0.018 | 0.133 | 2096 | 0.028 | 0.164 | 29418 |
| IADLs or ADLs: 0 | 0.548 | 0.498 | 31514 | 0.433 | 0.496 | 2096 | 0.556 | 0.497 | 29418 |
| IADLs: 1 | 0.155 | 0.362 | 31514 | 0.162 | 0.368 | 2096 | 0.155 | 0.362 | 29418 |
| ADLs: 1-2 | 0.201 | 0.400 | 31514 | 0.260 | 0.438 | 2096 | 0.196 | 0.397 | 29418 |
| ADLs: 3-4 | 0.065 | 0.247 | 31514 | 0.098 | 0.298 | 2096 | 0.063 | 0.243 | 29418 |
| ADLs: 5-6 | 0.031 | 0.173 | 31514 | 0.048 | 0.213 | 2096 | 0.030 | 0.169 | 29418 |
| Chronic Conditions (except ADRD): 0 | 0.052 | 0.222 | 31514 | 0.000 | 0.000 | 2096 | 0.056 | 0.230 | 29418 |
| Chronic Conditions (except ADRD): 1 | 0.108 | 0.310 | 31514 | 0.004 | 0.062 | 2096 | 0.115 | 0.320 | 29418 |

© 2026 Fu W et al. *JAMA Netw. Open*.

| | | | | | | | | | |
|---|---|---|---|---|---|---|---|---|---|
| Chronic Conditions (except ADRD): 2 | 0.167 | 0.373 | 31514 | 0.017 | 0.130 | 2096 | 0.178 | 0.382 | 29418 |
| Chronic Conditions (except ADRD): 3-4 | 0.370 | 0.483 | 31514 | 0.145 | 0.352 | 2096 | 0.386 | 0.487 | 29418 |
| Chronic Conditions (except ADRD): 5-6 | 0.206 | 0.405 | 31514 | 0.331 | 0.471 | 2096 | 0.197 | 0.398 | 29418 |
| Chronic Conditions (except ADRD): 7-10 | 0.092 | 0.289 | 31514 | 0.452 | 0.498 | 2096 | 0.067 | 0.249 | 29418 |
| Chronic Conditions (except ADRD): 11+ | 0.005 | 0.067 | 31514 | 0.051 | 0.219 | 2096 | 0.001 | 0.035 | 29418 |

Notes: The working sample consists of MA beneficiaries from 2015-2022 MCBS. Beneficiaries with only partial enrollment in an MA plan during the past year at the time of the survey and veterans are excluded. We also exclude beneficiaries with ADRD.

© 2026 Fu W et al. *JAMA Netw. Open*.

**eTable 7. Summary Statistics: Negative Control Group: Hypertension/high BP (non-ADRD), Comparison Group: non Hypertension/high BP (non ADRD)**

| | (1) | | | (2) | | | (3) | | |
|---|---|---|---|---|---|---|---|---|---|
| | Negative Control + Comparison | | | Negative Control | | | Comparison | | |
| | Mean | SD | N | Mean | SD | N | Mean | SD | N |
| Any Troubles Getting Needed Care | 0.077 | 0.267 | 31422 | 0.074 | 0.263 | 20751 | 0.082 | 0.274 | 10671 |
| Any Medical Financial Burden | 0.183 | 0.387 | 25184 | 0.191 | 0.393 | 16576 | 0.169 | 0.374 | 8608 |
| Satisfaction with Access to Specialist | 0.935 | 0.247 | 29250 | 0.938 | 0.242 | 19567 | 0.929 | 0.256 | 9683 |
| Satisfaction with Quality of Care | 0.952 | 0.214 | 30843 | 0.954 | 0.209 | 20548 | 0.948 | 0.223 | 10295 |
| Comparison | 0.340 | 0.474 | 31471 | 0.000 | 0.000 | 20786 | 1.000 | 0.000 | 10685 |
| Negative Control | 0.660 | 0.474 | 31471 | 1.000 | 0.000 | 20786 | 0.000 | 0.000 | 10685 |
| Age Group <65 | 0.168 | 0.374 | 31471 | 0.139 | 0.346 | 20786 | 0.225 | 0.417 | 10685 |
| Age Group 65 to 75 | 0.353 | 0.478 | 31471 | 0.338 | 0.473 | 20786 | 0.380 | 0.485 | 10685 |
| Age Group 75+ | 0.479 | 0.500 | 31471 | 0.522 | 0.500 | 20786 | 0.395 | 0.489 | 10685 |
| Non-Hispanic white | 0.656 | 0.475 | 31471 | 0.620 | 0.486 | 20786 | 0.726 | 0.446 | 10685 |
| Non-Hispanic black | 0.129 | 0.335 | 31471 | 0.154 | 0.361 | 20786 | 0.079 | 0.269 | 10685 |
| Hispanic | 0.158 | 0.364 | 31471 | 0.167 | 0.373 | 20786 | 0.140 | 0.347 | 10685 |
| Other | 0.058 | 0.233 | 31471 | 0.059 | 0.236 | 20786 | 0.055 | 0.229 | 10685 |
| Male | 0.334 | 0.472 | 31471 | 0.328 | 0.469 | 20786 | 0.346 | 0.476 | 10685 |
| Female | 0.666 | 0.472 | 31471 | 0.672 | 0.469 | 20786 | 0.654 | 0.476 | 10685 |
| Not Married | 0.473 | 0.499 | 31471 | 0.485 | 0.500 | 20786 | 0.449 | 0.497 | 10685 |
| Married | 0.367 | 0.482 | 31471 | 0.357 | 0.479 | 20786 | 0.386 | 0.487 | 10685 |
| Married: missing | 0.160 | 0.367 | 31471 | 0.158 | 0.364 | 20786 | 0.165 | 0.371 | 10685 |
| High school or less | 0.476 | 0.499 | 31471 | 0.504 | 0.500 | 20786 | 0.423 | 0.494 | 10685 |
| High School above | 0.361 | 0.480 | 31471 | 0.336 | 0.472 | 20786 | 0.409 | 0.492 | 10685 |
| Education missing | 0.163 | 0.369 | 31471 | 0.161 | 0.367 | 20786 | 0.168 | 0.374 | 10685 |
| Self-respondent | 0.921 | 0.270 | 31471 | 0.923 | 0.266 | 20786 | 0.915 | 0.279 | 10685 |
| Proxy respondent | 0.079 | 0.270 | 31471 | 0.077 | 0.266 | 20786 | 0.085 | 0.279 | 10685 |
| Household Size: 1 | 0.335 | 0.472 | 31471 | 0.347 | 0.476 | 20786 | 0.310 | 0.463 | 10685 |
| Household Size: 2 | 0.450 | 0.498 | 31471 | 0.438 | 0.496 | 20786 | 0.475 | 0.499 | 10685 |
| Household Size: 3+ | 0.215 | 0.411 | 31471 | 0.215 | 0.411 | 20786 | 0.214 | 0.410 | 10685 |
| Underweight/healthy (<25) | 0.304 | 0.460 | 31471 | 0.255 | 0.436 | 20786 | 0.400 | 0.490 | 10685 |
| Overweight (25-30) | 0.340 | 0.474 | 31471 | 0.341 | 0.474 | 20786 | 0.337 | 0.473 | 10685 |
| Obese/high-risk obese (>=30) | 0.330 | 0.470 | 31471 | 0.378 | 0.485 | 20786 | 0.235 | 0.424 | 10685 |
| BMI missing | 0.027 | 0.162 | 31471 | 0.027 | 0.161 | 20786 | 0.028 | 0.164 | 10685 |
| IADLs or ADLs: 0 | 0.548 | 0.498 | 31471 | 0.514 | 0.500 | 20786 | 0.616 | 0.486 | 10685 |
| IADLs: 1 | 0.155 | 0.362 | 31471 | 0.155 | 0.362 | 20786 | 0.156 | 0.363 | 10685 |
| ADLs: 1-2 | 0.201 | 0.400 | 31471 | 0.224 | 0.417 | 20786 | 0.154 | 0.361 | 10685 |
| ADLs: 3-4 | 0.065 | 0.247 | 31471 | 0.074 | 0.261 | 20786 | 0.049 | 0.215 | 10685 |
| ADLs: 5-6 | 0.031 | 0.173 | 31471 | 0.034 | 0.180 | 20786 | 0.025 | 0.156 | 10685 |
| Chronic Conditions (except ADRD): 0 | 0.052 | 0.222 | 31471 | 0.000 | 0.000 | 20786 | 0.154 | 0.361 | 10685 |
| Chronic Conditions (except ADRD): 1 | 0.108 | 0.310 | 31471 | 0.041 | 0.198 | 20786 | 0.238 | 0.426 | 10685 |

| | | | | | | | | | |
|---|---|---|---|---|---|---|---|---|---|
| Chronic Conditions (except ADRD): 2 | 0.167 | 0.373 | 31471 | 0.133 | 0.340 | 20786 | 0.232 | 0.422 | 10685 |
| Chronic Conditions (except ADRD): 3-4 | 0.370 | 0.483 | 31471 | 0.419 | 0.493 | 20786 | 0.274 | 0.446 | 10685 |
| Chronic Conditions (except ADRD): 5-6 | 0.206 | 0.405 | 31471 | 0.269 | 0.444 | 20786 | 0.084 | 0.277 | 10685 |
| Chronic Conditions (except ADRD): 7-10 | 0.092 | 0.290 | 31471 | 0.131 | 0.337 | 20786 | 0.018 | 0.131 | 10685 |
| Chronic Conditions (except ADRD): 11+ | 0.005 | 0.067 | 31471 | 0.007 | 0.082 | 20786 | 0.000 | 0.017 | 10685 |

Notes: The working sample consists of MA beneficiaries from 2015-2022 MCBS. Beneficiaries with only partial enrollment in an MA plan during the past year at the time of the survey and veterans are excluded. We also exclude beneficiaries with ADRD.

© 2026 Fu W et al. *JAMA Netw. Open*.

**eTable 8. Summary Statistics: Negative Control Group: MI/heart attack (non-ADRD), Comparison Group: non MI/heart attack (non ADRD)**

| | (1) | | | (2) | | | (3) | | |
|---|---|---|---|---|---|---|---|---|---|
| | Negative Control + Comparison | | | Negative Control | | | Comparison | | |
| | Mean | SD | N | Mean | SD | N | Mean | SD | N |
| Any Troubles Getting Needed Care | 0.077 | 0.266 | 31387 | 0.080 | 0.272 | 2989 | 0.077 | 0.266 | 28398 |
| Any Medical Financial Burden | 0.183 | 0.387 | 25156 | 0.260 | 0.439 | 2350 | 0.175 | 0.380 | 22806 |
| Satisfaction with Access to Specialist | 0.935 | 0.247 | 29216 | 0.941 | 0.235 | 2887 | 0.934 | 0.248 | 26329 |
| Satisfaction with Quality of Care | 0.952 | 0.214 | 30810 | 0.941 | 0.235 | 2966 | 0.953 | 0.211 | 27844 |
| Comparison | 0.905 | 0.294 | 31436 | 0.000 | 0.000 | 2998 | 1.000 | 0.000 | 28438 |
| Negative Control | 0.095 | 0.294 | 31436 | 1.000 | 0.000 | 2998 | 0.000 | 0.000 | 28438 |
| Age Group <65 | 0.168 | 0.374 | 31436 | 0.142 | 0.349 | 2998 | 0.171 | 0.377 | 28438 |
| Age Group 65 to 75 | 0.353 | 0.478 | 31436 | 0.296 | 0.457 | 2998 | 0.358 | 0.480 | 28438 |
| Age Group 75+ | 0.479 | 0.500 | 31436 | 0.562 | 0.496 | 2998 | 0.470 | 0.499 | 28438 |
| Non-Hispanic white | 0.656 | 0.475 | 31436 | 0.653 | 0.476 | 2998 | 0.656 | 0.475 | 28438 |
| Non-Hispanic black | 0.129 | 0.335 | 31436 | 0.121 | 0.326 | 2998 | 0.130 | 0.336 | 28438 |
| Hispanic | 0.158 | 0.364 | 31436 | 0.174 | 0.379 | 2998 | 0.156 | 0.363 | 28438 |
| Other | 0.058 | 0.233 | 31436 | 0.052 | 0.223 | 2998 | 0.058 | 0.234 | 28438 |
| Male | 0.334 | 0.472 | 31436 | 0.469 | 0.499 | 2998 | 0.320 | 0.466 | 28438 |
| Female | 0.666 | 0.472 | 31436 | 0.531 | 0.499 | 2998 | 0.680 | 0.466 | 28438 |
| Not Married | 0.473 | 0.499 | 31436 | 0.473 | 0.499 | 2998 | 0.473 | 0.499 | 28438 |
| Married | 0.367 | 0.482 | 31436 | 0.385 | 0.487 | 2998 | 0.365 | 0.482 | 28438 |
| Married: missing | 0.160 | 0.367 | 31436 | 0.142 | 0.350 | 2998 | 0.162 | 0.368 | 28438 |
| High school or less | 0.476 | 0.499 | 31436 | 0.548 | 0.498 | 2998 | 0.469 | 0.499 | 28438 |
| High School above | 0.361 | 0.480 | 31436 | 0.309 | 0.462 | 2998 | 0.366 | 0.482 | 28438 |
| Education missing | 0.163 | 0.369 | 31436 | 0.144 | 0.351 | 2998 | 0.165 | 0.371 | 28438 |
| Self-respondent | 0.921 | 0.270 | 31436 | 0.900 | 0.300 | 2998 | 0.923 | 0.267 | 28438 |
| Proxy respondent | 0.079 | 0.270 | 31436 | 0.100 | 0.300 | 2998 | 0.077 | 0.267 | 28438 |
| Household Size: 1 | 0.335 | 0.472 | 31436 | 0.321 | 0.467 | 2998 | 0.336 | 0.472 | 28438 |
| Household Size: 2 | 0.450 | 0.498 | 31436 | 0.434 | 0.496 | 2998 | 0.452 | 0.498 | 28438 |
| Household Size: 3+ | 0.215 | 0.411 | 31436 | 0.245 | 0.430 | 2998 | 0.212 | 0.408 | 28438 |
| Underweight/healthy (<25) | 0.304 | 0.460 | 31436 | 0.279 | 0.448 | 2998 | 0.307 | 0.461 | 28438 |
| Overweight (25-30) | 0.340 | 0.474 | 31436 | 0.355 | 0.479 | 2998 | 0.338 | 0.473 | 28438 |
| Obese/high-risk obese (>=30) | 0.329 | 0.470 | 31436 | 0.341 | 0.474 | 2998 | 0.328 | 0.470 | 28438 |
| BMI missing | 0.027 | 0.162 | 31436 | 0.026 | 0.159 | 2998 | 0.027 | 0.162 | 28438 |
| IADLs or ADLs: 0 | 0.549 | 0.498 | 31436 | 0.441 | 0.497 | 2998 | 0.560 | 0.496 | 28438 |
| IADLs: 1 | 0.155 | 0.362 | 31436 | 0.152 | 0.359 | 2998 | 0.155 | 0.362 | 28438 |
| ADLs: 1-2 | 0.200 | 0.400 | 31436 | 0.257 | 0.437 | 2998 | 0.194 | 0.396 | 28438 |
| ADLs: 3-4 | 0.065 | 0.247 | 31436 | 0.100 | 0.301 | 2998 | 0.062 | 0.240 | 28438 |
| ADLs: 5-6 | 0.031 | 0.172 | 31436 | 0.049 | 0.217 | 2998 | 0.029 | 0.167 | 28438 |
| Chronic Conditions (except ADRD): 0 | 0.052 | 0.222 | 31436 | 0.000 | 0.000 | 2998 | 0.058 | 0.233 | 28438 |
| Chronic Conditions (except ADRD): 1 | 0.108 | 0.310 | 31436 | 0.009 | 0.093 | 2998 | 0.118 | 0.323 | 28438 |

© 2026 Fu W et al. *JAMA Netw. Open*.

| | | | | | | | | | |
|---|---|---|---|---|---|---|---|---|---|
| Chronic Conditions (except ADRD): 2 | 0.167 | 0.373 | 31436 | 0.029 | 0.167 | 2998 | 0.182 | 0.385 | 28438 |
| Chronic Conditions (except ADRD): 3-4 | 0.370 | 0.483 | 31436 | 0.201 | 0.401 | 2998 | 0.388 | 0.487 | 28438 |
| Chronic Conditions (except ADRD): 5-6 | 0.206 | 0.405 | 31436 | 0.346 | 0.476 | 2998 | 0.191 | 0.393 | 28438 |
| Chronic Conditions (except ADRD): 7-10 | 0.092 | 0.289 | 31436 | 0.383 | 0.486 | 2998 | 0.062 | 0.241 | 28438 |
| Chronic Conditions (except ADRD): 11+ | 0.005 | 0.067 | 31436 | 0.033 | 0.179 | 2998 | 0.002 | 0.039 | 28438 |

Notes: The working sample consists of MA beneficiaries from 2015-2022 MCBS. Beneficiaries with only partial enrollment in an MA plan during the past year at the time of the survey and veterans are excluded. We also exclude beneficiaries with ADRD.

© 2026 Fu W et al. *JAMA Netw. Open*.

**eTable 9. Summary Statistics: Negative Control Group: Angina pectoris/CHD (non-ADRD), Comparison Group: non Angina pectoris/CHD (non ADRD)**

| | (1) Negative Control + Comparison | | | (2) Negative Control | | | (3) Comparison | | |
|---|---|---|---|---|---|---|---|---|---|
| | Mean | SD | N | Mean | SD | N | Mean | SD | N |
| Any Troubles Getting Needed Care | 0.077 | 0.266 | 31254 | 0.083 | 0.276 | 2406 | 0.076 | 0.266 | 28848 |
| Any Medical Financial Burden | 0.183 | 0.386 | 25047 | 0.277 | 0.447 | 1894 | 0.175 | 0.380 | 23153 |
| Satisfaction with Access to Specialist | 0.935 | 0.247 | 29088 | 0.930 | 0.256 | 2351 | 0.935 | 0.246 | 26737 |
| Satisfaction with Quality of Care | 0.952 | 0.214 | 30678 | 0.940 | 0.237 | 2394 | 0.953 | 0.212 | 28284 |
| Comparison | 0.923 | 0.267 | 31302 | 0.000 | 0.000 | 2411 | 1.000 | 0.000 | 28891 |
| Negative Control | 0.077 | 0.267 | 31302 | 1.000 | 0.000 | 2411 | 0.000 | 0.000 | 28891 |
| Age Group <65 | 0.168 | 0.374 | 31302 | 0.133 | 0.340 | 2411 | 0.171 | 0.377 | 28891 |
| Age Group 65 to 75 | 0.353 | 0.478 | 31302 | 0.325 | 0.468 | 2411 | 0.355 | 0.479 | 28891 |
| Age Group 75+ | 0.478 | 0.500 | 31302 | 0.542 | 0.498 | 2411 | 0.473 | 0.499 | 28891 |
| Non-Hispanic white | 0.656 | 0.475 | 31302 | 0.689 | 0.463 | 2411 | 0.653 | 0.476 | 28891 |
| Non-Hispanic black | 0.129 | 0.335 | 31302 | 0.105 | 0.307 | 2411 | 0.131 | 0.337 | 28891 |
| Hispanic | 0.158 | 0.365 | 31302 | 0.152 | 0.359 | 2411 | 0.158 | 0.365 | 28891 |
| Other | 0.058 | 0.233 | 31302 | 0.053 | 0.224 | 2411 | 0.058 | 0.234 | 28891 |
| Male | 0.334 | 0.472 | 31302 | 0.450 | 0.498 | 2411 | 0.324 | 0.468 | 28891 |
| Female | 0.666 | 0.472 | 31302 | 0.550 | 0.498 | 2411 | 0.676 | 0.468 | 28891 |
| Not Married | 0.473 | 0.499 | 31302 | 0.470 | 0.499 | 2411 | 0.473 | 0.499 | 28891 |
| Married | 0.367 | 0.482 | 31302 | 0.389 | 0.488 | 2411 | 0.365 | 0.482 | 28891 |
| Married: missing | 0.160 | 0.366 | 31302 | 0.141 | 0.348 | 2411 | 0.161 | 0.368 | 28891 |
| High school or less | 0.476 | 0.499 | 31302 | 0.491 | 0.500 | 2411 | 0.475 | 0.499 | 28891 |
| High School above | 0.361 | 0.480 | 31302 | 0.367 | 0.482 | 2411 | 0.361 | 0.480 | 28891 |
| Education missing | 0.163 | 0.369 | 31302 | 0.142 | 0.349 | 2411 | 0.165 | 0.371 | 28891 |
| Self-respondent | 0.921 | 0.270 | 31302 | 0.916 | 0.277 | 2411 | 0.921 | 0.270 | 28891 |
| Proxy respondent | 0.079 | 0.270 | 31302 | 0.084 | 0.277 | 2411 | 0.079 | 0.270 | 28891 |
| Household Size: 1 | 0.335 | 0.472 | 31302 | 0.312 | 0.464 | 2411 | 0.337 | 0.473 | 28891 |
| Household Size: 2 | 0.450 | 0.498 | 31302 | 0.474 | 0.499 | 2411 | 0.448 | 0.497 | 28891 |
| Household Size: 3+ | 0.215 | 0.411 | 31302 | 0.214 | 0.410 | 2411 | 0.215 | 0.411 | 28891 |
| Underweight/healthy (<25) | 0.304 | 0.460 | 31302 | 0.233 | 0.423 | 2411 | 0.310 | 0.463 | 28891 |
| Overweight (25-30) | 0.340 | 0.474 | 31302 | 0.379 | 0.485 | 2411 | 0.336 | 0.472 | 28891 |
| Obese/high-risk obese (>=30) | 0.329 | 0.470 | 31302 | 0.369 | 0.483 | 2411 | 0.326 | 0.469 | 28891 |
| BMI missing | 0.027 | 0.162 | 31302 | 0.019 | 0.137 | 2411 | 0.028 | 0.164 | 28891 |
| IADLs or ADLs: 0 | 0.550 | 0.498 | 31302 | 0.415 | 0.493 | 2411 | 0.561 | 0.496 | 28891 |
| IADLs: 1 | 0.155 | 0.362 | 31302 | 0.173 | 0.379 | 2411 | 0.153 | 0.360 | 28891 |
| ADLs: 1-2 | 0.200 | 0.400 | 31302 | 0.259 | 0.438 | 2411 | 0.195 | 0.396 | 28891 |
| ADLs: 3-4 | 0.065 | 0.246 | 31302 | 0.100 | 0.301 | 2411 | 0.062 | 0.241 | 28891 |
| ADLs: 5-6 | 0.031 | 0.172 | 31302 | 0.052 | 0.223 | 2411 | 0.029 | 0.167 | 28891 |
| Chronic Conditions (except ADRD): 0 | 0.052 | 0.223 | 31302 | 0.000 | 0.000 | 2411 | 0.057 | 0.231 | 28891 |
| Chronic Conditions (except ADRD): 1 | 0.108 | 0.311 | 31302 | 0.001 | 0.029 | 2411 | 0.117 | 0.322 | 28891 |

| | | | | | | | | | |
|---|---|---|---|---|---|---|---|---|---|
| Chronic Conditions (except ADRD): 2 | 0.167 | 0.373 | 31302 | 0.011 | 0.103 | 2411 | 0.180 | 0.384 | 28891 |
| Chronic Conditions (except ADRD): 3-4 | 0.370 | 0.483 | 31302 | 0.139 | 0.346 | 2411 | 0.389 | 0.488 | 28891 |
| Chronic Conditions (except ADRD): 5-6 | 0.205 | 0.404 | 31302 | 0.339 | 0.473 | 2411 | 0.194 | 0.396 | 28891 |
| Chronic Conditions (except ADRD): 7-10 | 0.092 | 0.289 | 31302 | 0.460 | 0.499 | 2411 | 0.061 | 0.240 | 28891 |
| Chronic Conditions (except ADRD): 11+ | 0.005 | 0.067 | 31302 | 0.050 | 0.218 | 2411 | 0.001 | 0.027 | 28891 |

Notes: The working sample consists of MA beneficiaries from 2015-2022 MCBS. Beneficiaries with only partial enrollment in an MA plan during the past year at the time of the survey and veterans are excluded. We also exclude beneficiaries with ADRD.

© 2026 Fu W et al. *JAMA Netw. Open*.

**eTable 10. Summary Statistics: Negative Control Group: Congestive Heart Failure (non-ADRD), Comparison Group: non Congestive heart failure (non ADRD)**

| | (1) Negative Control + Comparison | | | (2) Negative Control | | | (3) Comparison | | |
|---|---|---|---|---|---|---|---|---|---|
| | Mean | SD | N | Mean | SD | N | Mean | SD | N |
| Any Troubles Getting Needed Care | 0.077 | 0.266 | 31205 | 0.094 | 0.292 | 1934 | 0.076 | 0.265 | 29271 |
| Any Medical Financial Burden | 0.182 | 0.386 | 25014 | 0.305 | 0.460 | 1550 | 0.174 | 0.379 | 23464 |
| Satisfaction with Access to Specialist | 0.935 | 0.247 | 29039 | 0.928 | 0.258 | 1886 | 0.935 | 0.246 | 27153 |
| Satisfaction with Quality of Care | 0.952 | 0.214 | 30629 | 0.926 | 0.262 | 1923 | 0.954 | 0.210 | 28706 |
| Comparison | 0.938 | 0.241 | 31253 | 0.000 | 0.000 | 1941 | 1.000 | 0.000 | 29312 |
| Negative Control | 0.062 | 0.241 | 31253 | 1.000 | 0.000 | 1941 | 0.000 | 0.000 | 29312 |
| Age Group <65 | 0.169 | 0.375 | 31253 | 0.190 | 0.392 | 1941 | 0.167 | 0.373 | 29312 |
| Age Group 65 to 75 | 0.353 | 0.478 | 31253 | 0.251 | 0.434 | 1941 | 0.360 | 0.480 | 29312 |
| Age Group 75+ | 0.478 | 0.500 | 31253 | 0.559 | 0.497 | 1941 | 0.473 | 0.499 | 29312 |
| Non-Hispanic white | 0.656 | 0.475 | 31253 | 0.639 | 0.480 | 1941 | 0.657 | 0.475 | 29312 |
| Non-Hispanic black | 0.129 | 0.335 | 31253 | 0.185 | 0.389 | 1941 | 0.125 | 0.331 | 29312 |
| Hispanic | 0.158 | 0.364 | 31253 | 0.123 | 0.329 | 1941 | 0.160 | 0.367 | 29312 |
| Other | 0.058 | 0.233 | 31253 | 0.052 | 0.222 | 1941 | 0.058 | 0.234 | 29312 |
| Male | 0.334 | 0.472 | 31253 | 0.351 | 0.478 | 1941 | 0.333 | 0.471 | 29312 |
| Female | 0.666 | 0.472 | 31253 | 0.649 | 0.478 | 1941 | 0.667 | 0.471 | 29312 |
| Not Married | 0.473 | 0.499 | 31253 | 0.545 | 0.498 | 1941 | 0.468 | 0.499 | 29312 |
| Married | 0.367 | 0.482 | 31253 | 0.302 | 0.459 | 1941 | 0.372 | 0.483 | 29312 |
| Married: missing | 0.160 | 0.367 | 31253 | 0.153 | 0.360 | 1941 | 0.160 | 0.367 | 29312 |
| High school or less | 0.476 | 0.499 | 31253 | 0.546 | 0.498 | 1941 | 0.471 | 0.499 | 29312 |
| High School above | 0.361 | 0.480 | 31253 | 0.300 | 0.458 | 1941 | 0.365 | 0.482 | 29312 |
| Education missing | 0.163 | 0.369 | 31253 | 0.155 | 0.362 | 1941 | 0.164 | 0.370 | 29312 |
| Self-respondent | 0.921 | 0.270 | 31253 | 0.887 | 0.316 | 1941 | 0.923 | 0.267 | 29312 |
| Proxy respondent | 0.079 | 0.270 | 31253 | 0.113 | 0.316 | 1941 | 0.077 | 0.267 | 29312 |
| Household Size: 1 | 0.334 | 0.472 | 31253 | 0.388 | 0.487 | 1941 | 0.331 | 0.471 | 29312 |
| Household Size: 2 | 0.450 | 0.498 | 31253 | 0.380 | 0.485 | 1941 | 0.455 | 0.498 | 29312 |
| Household Size: 3+ | 0.215 | 0.411 | 31253 | 0.232 | 0.422 | 1941 | 0.214 | 0.410 | 29312 |
| Underweight/healthy (<25) | 0.304 | 0.460 | 31253 | 0.230 | 0.421 | 1941 | 0.309 | 0.462 | 29312 |
| Overweight (25-30) | 0.340 | 0.474 | 31253 | 0.308 | 0.462 | 1941 | 0.342 | 0.474 | 29312 |
| Obese/high-risk obese (>=30) | 0.329 | 0.470 | 31253 | 0.439 | 0.496 | 1941 | 0.322 | 0.467 | 29312 |
| BMI missing | 0.027 | 0.162 | 31253 | 0.022 | 0.147 | 1941 | 0.027 | 0.163 | 29312 |
| IADLs or ADLs: 0 | 0.550 | 0.497 | 31253 | 0.303 | 0.460 | 1941 | 0.567 | 0.496 | 29312 |
| IADLs: 1 | 0.155 | 0.362 | 31253 | 0.174 | 0.379 | 1941 | 0.154 | 0.361 | 29312 |
| ADLs: 1-2 | 0.200 | 0.400 | 31253 | 0.332 | 0.471 | 1941 | 0.191 | 0.393 | 29312 |
| ADLs: 3-4 | 0.065 | 0.246 | 31253 | 0.122 | 0.327 | 1941 | 0.061 | 0.239 | 29312 |
| ADLs: 5-6 | 0.030 | 0.172 | 31253 | 0.068 | 0.252 | 1941 | 0.028 | 0.165 | 29312 |
| Chronic Conditions (except ADRD): 0 | 0.052 | 0.223 | 31253 | 0.000 | 0.000 | 1941 | 0.056 | 0.230 | 29312 |
| Chronic Conditions (except ADRD): 1 | 0.108 | 0.311 | 31253 | 0.001 | 0.032 | 1941 | 0.116 | 0.320 | 29312 |

© 2026 Fu W et al. *JAMA Netw. Open*.

| | | | | | | | | | |
|---|---|---|---|---|---|---|---|---|---|
| Chronic Conditions (except ADRD): 2 | 0.167 | 0.373 | 31253 | 0.025 | 0.155 | 1941 | 0.177 | 0.382 | 29312 |
| Chronic Conditions (except ADRD): 3-4 | 0.370 | 0.483 | 31253 | 0.159 | 0.366 | 1941 | 0.384 | 0.486 | 29312 |
| Chronic Conditions (except ADRD): 5-6 | 0.205 | 0.404 | 31253 | 0.301 | 0.459 | 1941 | 0.199 | 0.399 | 29312 |
| Chronic Conditions (except ADRD): 7-10 | 0.092 | 0.289 | 31253 | 0.457 | 0.498 | 1941 | 0.067 | 0.251 | 29312 |
| Chronic Conditions (except ADRD): 11+ | 0.005 | 0.067 | 31253 | 0.057 | 0.231 | 1941 | 0.001 | 0.033 | 29312 |

Notes: The working sample consists of MA beneficiaries from 2015-2022 MCBS. Beneficiaries with only partial enrollment in an MA plan during the past year at the time of the survey and veterans are excluded. We also exclude beneficiaries with ADRD.

© 2026 Fu W et al. *JAMA Netw. Open*.

**eTable 11. Summary Statistics: Negative Control Group: Other heart cond, eg valve/rhythm (non-ADRD), Comparison Group: non Other heart cond, eg valve/rhythm (non ADRD)**

| | (1) | | | (2) | | | (3) | | |
|---|---|---|---|---|---|---|---|---|---|
| | Negative Control + Comparison | | | Negative Control | | | Comparison | | |
| | Mean | SD | N | Mean | SD | N | Mean | SD | N |
| Any Troubles Getting Needed Care | 0.078 | 0.268 | 29258 | 0.083 | 0.276 | 6106 | 0.076 | 0.266 | 23152 |
| Any Medical Financial Burden | 0.182 | 0.386 | 24981 | 0.230 | 0.421 | 5175 | 0.170 | 0.376 | 19806 |
| Satisfaction with Access to Specialist | 0.934 | 0.248 | 27325 | 0.927 | 0.260 | 5902 | 0.936 | 0.245 | 21423 |
| Satisfaction with Quality of Care | 0.951 | 0.215 | 28790 | 0.947 | 0.224 | 6051 | 0.953 | 0.213 | 22739 |
| Comparison | 0.791 | 0.406 | 29306 | 0.000 | 0.000 | 6115 | 1.000 | 0.000 | 23191 |
| Negative Control | 0.209 | 0.406 | 29306 | 1.000 | 0.000 | 6115 | 0.000 | 0.000 | 23191 |
| Age Group <65 | 0.170 | 0.375 | 29306 | 0.141 | 0.348 | 6115 | 0.177 | 0.382 | 23191 |
| Age Group 65 to 75 | 0.351 | 0.477 | 29306 | 0.292 | 0.455 | 6115 | 0.367 | 0.482 | 23191 |
| Age Group 75+ | 0.479 | 0.500 | 29306 | 0.567 | 0.496 | 6115 | 0.456 | 0.498 | 23191 |
| Non-Hispanic white | 0.656 | 0.475 | 29306 | 0.738 | 0.440 | 6115 | 0.634 | 0.482 | 23191 |
| Non-Hispanic black | 0.130 | 0.336 | 29306 | 0.099 | 0.298 | 6115 | 0.138 | 0.345 | 23191 |
| Hispanic | 0.157 | 0.363 | 29306 | 0.105 | 0.306 | 6115 | 0.170 | 0.376 | 23191 |
| Other | 0.058 | 0.233 | 29306 | 0.059 | 0.235 | 6115 | 0.058 | 0.233 | 23191 |
| Male | 0.336 | 0.472 | 29306 | 0.331 | 0.471 | 6115 | 0.337 | 0.473 | 23191 |
| Female | 0.664 | 0.472 | 29306 | 0.669 | 0.471 | 6115 | 0.663 | 0.473 | 23191 |
| Not Married | 0.468 | 0.499 | 29306 | 0.463 | 0.499 | 6115 | 0.469 | 0.499 | 23191 |
| Married | 0.362 | 0.481 | 29306 | 0.372 | 0.483 | 6115 | 0.359 | 0.480 | 23191 |
| Married: missing | 0.170 | 0.376 | 29306 | 0.165 | 0.372 | 6115 | 0.172 | 0.377 | 23191 |
| High school or less | 0.466 | 0.499 | 29306 | 0.449 | 0.497 | 6115 | 0.471 | 0.499 | 23191 |
| High School above | 0.360 | 0.480 | 29306 | 0.384 | 0.486 | 6115 | 0.354 | 0.478 | 23191 |
| Education missing | 0.173 | 0.378 | 29306 | 0.167 | 0.373 | 6115 | 0.175 | 0.380 | 23191 |
| Self-respondent | 0.920 | 0.271 | 29306 | 0.929 | 0.257 | 6115 | 0.918 | 0.274 | 23191 |
| Proxy respondent | 0.080 | 0.271 | 29306 | 0.071 | 0.257 | 6115 | 0.082 | 0.274 | 23191 |
| Household Size: 1 | 0.334 | 0.472 | 29306 | 0.339 | 0.473 | 6115 | 0.332 | 0.471 | 23191 |
| Household Size: 2 | 0.450 | 0.498 | 29306 | 0.459 | 0.498 | 6115 | 0.448 | 0.497 | 23191 |
| Household Size: 3+ | 0.216 | 0.412 | 29306 | 0.203 | 0.402 | 6115 | 0.220 | 0.414 | 23191 |
| Underweight/healthy (<25) | 0.304 | 0.460 | 29306 | 0.280 | 0.449 | 6115 | 0.310 | 0.463 | 23191 |
| Overweight (25-30) | 0.339 | 0.473 | 29306 | 0.348 | 0.476 | 6115 | 0.336 | 0.472 | 23191 |
| Obese/high-risk obese (>=30) | 0.330 | 0.470 | 29306 | 0.353 | 0.478 | 6115 | 0.324 | 0.468 | 23191 |
| BMI missing | 0.027 | 0.163 | 29306 | 0.019 | 0.138 | 6115 | 0.030 | 0.169 | 23191 |
| IADLs or ADLs: 0 | 0.553 | 0.497 | 29306 | 0.464 | 0.499 | 6115 | 0.576 | 0.494 | 23191 |
| IADLs: 1 | 0.156 | 0.363 | 29306 | 0.168 | 0.374 | 6115 | 0.153 | 0.360 | 23191 |
| ADLs: 1-2 | 0.196 | 0.397 | 29306 | 0.248 | 0.432 | 6115 | 0.182 | 0.386 | 23191 |
| ADLs: 3-4 | 0.065 | 0.246 | 29306 | 0.084 | 0.278 | 6115 | 0.060 | 0.237 | 23191 |
| ADLs: 5-6 | 0.031 | 0.172 | 29306 | 0.036 | 0.185 | 6115 | 0.029 | 0.168 | 23191 |
| Chronic Conditions (except ADRD): 0 | 0.053 | 0.223 | 29306 | 0.000 | 0.000 | 6115 | 0.067 | 0.249 | 23191 |

© 2026 Fu W et al. *JAMA Netw. Open*.

| | | | | | | | | | |
|---|---|---|---|---|---|---|---|---|---|
| Chronic Conditions (except ADRD): 1 | 0.107 | 0.309 | 29306 | 0.022 | 0.146 | 6115 | 0.130 | 0.336 | 23191 |
| Chronic Conditions (except ADRD): 2 | 0.167 | 0.373 | 29306 | 0.061 | 0.239 | 6115 | 0.194 | 0.396 | 23191 |
| Chronic Conditions (except ADRD): 3-4 | 0.367 | 0.482 | 29306 | 0.313 | 0.464 | 6115 | 0.382 | 0.486 | 23191 |
| Chronic Conditions (except ADRD): 5-6 | 0.207 | 0.405 | 29306 | 0.338 | 0.473 | 6115 | 0.173 | 0.378 | 23191 |
| Chronic Conditions (except ADRD): 7-10 | 0.094 | 0.292 | 29306 | 0.247 | 0.431 | 6115 | 0.054 | 0.226 | 23191 |
| Chronic Conditions (except ADRD): 11+ | 0.005 | 0.069 | 29306 | 0.019 | 0.138 | 6115 | 0.001 | 0.031 | 23191 |

Notes: The working sample consists of MA beneficiaries from 2015-2022 MCBS. Beneficiaries with only partial enrollment in an MA plan during the past year at the time of the survey and veterans are excluded. We also exclude beneficiaries with ADRD.

© 2026 Fu W et al. *JAMA Netw. Open*.

**eTable 12. Summary Statistics: Negative Control Group: High cholesterol (non-ADRD), Comparison Group: non High cholesterol (non ADRD)**

| | (1) | | | (2) | | | (3) | | |
|---|---|---|---|---|---|---|---|---|---|
| | Negative Control + Comparison | | | Negative Control | | | Comparison | | |
| | Mean | SD | N | Mean | SD | N | Mean | SD | N |
| Any Troubles Getting Needed Care | 0.078 | 0.268 | 29172 | 0.077 | 0.266 | 18768 | 0.079 | 0.270 | 10404 |
| Any Medical Financial Burden | 0.182 | 0.386 | 24912 | 0.188 | 0.391 | 16059 | 0.171 | 0.376 | 8853 |
| Satisfaction with Access to Specialist | 0.934 | 0.248 | 27254 | 0.936 | 0.244 | 17776 | 0.930 | 0.255 | 9478 |
| Satisfaction with Quality of Care | 0.952 | 0.214 | 28710 | 0.952 | 0.215 | 18616 | 0.952 | 0.214 | 10094 |
| Comparison | 0.357 | 0.479 | 29219 | 0.000 | 0.000 | 18798 | 1.000 | 0.000 | 10421 |
| Negative Control | 0.643 | 0.479 | 29219 | 1.000 | 0.000 | 18798 | 0.000 | 0.000 | 10421 |
| Age Group <65 | 0.170 | 0.375 | 29219 | 0.140 | 0.347 | 18798 | 0.223 | 0.417 | 10421 |
| Age Group 65 to 75 | 0.352 | 0.477 | 29219 | 0.359 | 0.480 | 18798 | 0.339 | 0.473 | 10421 |
| Age Group 75+ | 0.479 | 0.500 | 29219 | 0.501 | 0.500 | 18798 | 0.438 | 0.496 | 10421 |
| Non-Hispanic white | 0.656 | 0.475 | 29219 | 0.644 | 0.479 | 18798 | 0.676 | 0.468 | 10421 |
| Non-Hispanic black | 0.130 | 0.336 | 29219 | 0.130 | 0.336 | 18798 | 0.129 | 0.335 | 10421 |
| Hispanic | 0.157 | 0.364 | 29219 | 0.170 | 0.375 | 18798 | 0.134 | 0.341 | 10421 |
| Other | 0.058 | 0.233 | 29219 | 0.056 | 0.230 | 18798 | 0.061 | 0.239 | 10421 |
| Male | 0.336 | 0.472 | 29219 | 0.329 | 0.470 | 18798 | 0.349 | 0.477 | 10421 |
| Female | 0.664 | 0.472 | 29219 | 0.671 | 0.470 | 18798 | 0.651 | 0.477 | 10421 |
| Not Married | 0.468 | 0.499 | 29219 | 0.461 | 0.498 | 18798 | 0.479 | 0.500 | 10421 |
| Married | 0.362 | 0.481 | 29219 | 0.371 | 0.483 | 18798 | 0.346 | 0.476 | 10421 |
| Married: missing | 0.170 | 0.376 | 29219 | 0.168 | 0.374 | 18798 | 0.174 | 0.380 | 10421 |
| High school or less | 0.466 | 0.499 | 29219 | 0.484 | 0.500 | 18798 | 0.434 | 0.496 | 10421 |
| High School above | 0.361 | 0.480 | 29219 | 0.345 | 0.475 | 18798 | 0.388 | 0.487 | 10421 |
| Education missing | 0.173 | 0.378 | 29219 | 0.170 | 0.376 | 18798 | 0.178 | 0.382 | 10421 |
| Self-respondent | 0.921 | 0.270 | 29219 | 0.926 | 0.262 | 18798 | 0.911 | 0.285 | 10421 |
| Proxy respondent | 0.079 | 0.270 | 29219 | 0.074 | 0.262 | 18798 | 0.089 | 0.285 | 10421 |
| Household Size: 1 | 0.333 | 0.471 | 29219 | 0.331 | 0.471 | 18798 | 0.336 | 0.473 | 10421 |
| Household Size: 2 | 0.450 | 0.498 | 29219 | 0.455 | 0.498 | 18798 | 0.442 | 0.497 | 10421 |
| Household Size: 3+ | 0.216 | 0.412 | 29219 | 0.214 | 0.410 | 18798 | 0.221 | 0.415 | 10421 |
| Underweight/healthy (<25) | 0.304 | 0.460 | 29219 | 0.270 | 0.444 | 18798 | 0.366 | 0.482 | 10421 |
| Overweight (25-30) | 0.339 | 0.473 | 29219 | 0.356 | 0.479 | 18798 | 0.308 | 0.462 | 10421 |
| Obese/high-risk obese (>=30) | 0.330 | 0.470 | 29219 | 0.351 | 0.477 | 18798 | 0.294 | 0.455 | 10421 |
| BMI missing | 0.027 | 0.163 | 29219 | 0.024 | 0.153 | 18798 | 0.033 | 0.179 | 10421 |
| IADLs or ADLs: 0 | 0.554 | 0.497 | 29219 | 0.542 | 0.498 | 18798 | 0.574 | 0.495 | 10421 |
| IADLs: 1 | 0.156 | 0.363 | 29219 | 0.156 | 0.363 | 18798 | 0.156 | 0.363 | 10421 |
| ADLs: 1-2 | 0.195 | 0.396 | 29219 | 0.204 | 0.403 | 18798 | 0.180 | 0.384 | 10421 |
| ADLs: 3-4 | 0.065 | 0.246 | 29219 | 0.069 | 0.254 | 18798 | 0.056 | 0.231 | 10421 |
| ADLs: 5-6 | 0.030 | 0.172 | 29219 | 0.029 | 0.167 | 18798 | 0.034 | 0.180 | 10421 |
| Chronic Conditions (except ADRD): 0 | 0.052 | 0.223 | 29219 | 0.000 | 0.000 | 18798 | 0.147 | 0.354 | 10421 |
| Chronic Conditions (except ADRD): 1 | 0.107 | 0.309 | 29219 | 0.040 | 0.195 | 18798 | 0.228 | 0.420 | 10421 |

| | | | | | | | | | |
|---|---|---|---|---|---|---|---|---|---|
| Chronic Conditions (except ADRD): 2 | 0.166 | 0.373 | 29219 | 0.127 | 0.333 | 18798 | 0.237 | 0.425 | 10421 |
| Chronic Conditions (except ADRD): 3-4 | 0.368 | 0.482 | 29219 | 0.416 | 0.493 | 18798 | 0.281 | 0.449 | 10421 |
| Chronic Conditions (except ADRD): 5-6 | 0.207 | 0.405 | 29219 | 0.275 | 0.446 | 18798 | 0.086 | 0.280 | 10421 |
| Chronic Conditions (except ADRD): 7-10 | 0.094 | 0.292 | 29219 | 0.135 | 0.342 | 18798 | 0.020 | 0.142 | 10421 |
| Chronic Conditions (except ADRD): 11+ | 0.005 | 0.069 | 29219 | 0.007 | 0.084 | 18798 | 0.001 | 0.024 | 10421 |

Notes: The working sample consists of MA beneficiaries from 2015-2022 MCBS. Beneficiaries with only partial enrollment in an MA plan during the past year at the time of the survey and veterans are excluded. We also exclude beneficiaries with ADRD.

© 2026 Fu W et al. *JAMA Netw. Open*.

**eTable 13. Summary Statistics: Negative Control Group: Cancer (non-ADRD), Comparison Group: non Cancer (non ADRD)**

| | (1) Negative Control + Comparison | | | (2) Negative Control | | | (3) Comparison | | |
|---|---|---|---|---|---|---|---|---|---|
| | Mean | SD | N | Mean | SD | N | Mean | SD | N |
| Any Troubles Getting Needed Care | 0.078 | 0.268 | 29172 | 0.074 | 0.262 | 9066 | 0.079 | 0.270 | 20106 |
| Any Medical Financial Burden | 0.182 | 0.386 | 24912 | 0.178 | 0.382 | 7948 | 0.184 | 0.387 | 16964 |
| Satisfaction with Access to Specialist | 0.934 | 0.248 | 27254 | 0.937 | 0.243 | 8669 | 0.933 | 0.250 | 18585 |
| Satisfaction with Quality of Care | 0.952 | 0.214 | 28710 | 0.954 | 0.210 | 8991 | 0.951 | 0.217 | 19719 |
| Comparison | 0.689 | 0.463 | 29219 | 0.000 | 0.000 | 9074 | 1.000 | 0.000 | 20145 |
| Negative Control | 0.311 | 0.463 | 29219 | 1.000 | 0.000 | 9074 | 0.000 | 0.000 | 20145 |
| Age Group <65 | 0.170 | 0.375 | 29219 | 0.081 | 0.273 | 9074 | 0.210 | 0.407 | 20145 |
| Age Group 65 to 75 | 0.352 | 0.477 | 29219 | 0.328 | 0.469 | 9074 | 0.362 | 0.481 | 20145 |
| Age Group 75+ | 0.479 | 0.500 | 29219 | 0.591 | 0.492 | 9074 | 0.428 | 0.495 | 20145 |
| Non-Hispanic white | 0.656 | 0.475 | 29219 | 0.810 | 0.392 | 9074 | 0.586 | 0.492 | 20145 |
| Non-Hispanic black | 0.130 | 0.336 | 29219 | 0.057 | 0.232 | 9074 | 0.162 | 0.369 | 20145 |
| Hispanic | 0.157 | 0.364 | 29219 | 0.087 | 0.282 | 9074 | 0.188 | 0.391 | 20145 |
| Other | 0.058 | 0.233 | 29219 | 0.046 | 0.209 | 9074 | 0.063 | 0.243 | 20145 |
| Male | 0.336 | 0.472 | 29219 | 0.331 | 0.471 | 9074 | 0.338 | 0.473 | 20145 |
| Female | 0.664 | 0.472 | 29219 | 0.669 | 0.471 | 9074 | 0.662 | 0.473 | 20145 |
| Not Married | 0.468 | 0.499 | 29219 | 0.423 | 0.494 | 9074 | 0.487 | 0.500 | 20145 |
| Married | 0.362 | 0.481 | 29219 | 0.406 | 0.491 | 9074 | 0.343 | 0.475 | 20145 |
| Married: missing | 0.170 | 0.376 | 29219 | 0.171 | 0.376 | 9074 | 0.170 | 0.375 | 20145 |
| High school or less | 0.466 | 0.499 | 29219 | 0.401 | 0.490 | 9074 | 0.495 | 0.500 | 20145 |
| High School above | 0.361 | 0.480 | 29219 | 0.425 | 0.494 | 9074 | 0.332 | 0.471 | 20145 |
| Education missing | 0.173 | 0.378 | 29219 | 0.173 | 0.378 | 9074 | 0.173 | 0.378 | 20145 |
| Self-respondent | 0.921 | 0.270 | 29219 | 0.941 | 0.235 | 9074 | 0.911 | 0.284 | 20145 |
| Proxy respondent | 0.079 | 0.270 | 29219 | 0.059 | 0.235 | 9074 | 0.089 | 0.284 | 20145 |
| Household Size: 1 | 0.333 | 0.471 | 29219 | 0.340 | 0.474 | 9074 | 0.330 | 0.470 | 20145 |
| Household Size: 2 | 0.450 | 0.498 | 29219 | 0.495 | 0.500 | 9074 | 0.430 | 0.495 | 20145 |
| Household Size: 3+ | 0.216 | 0.412 | 29219 | 0.165 | 0.371 | 9074 | 0.240 | 0.427 | 20145 |
| Underweight/healthy (<25) | 0.304 | 0.460 | 29219 | 0.333 | 0.471 | 9074 | 0.291 | 0.454 | 20145 |
| Overweight (25-30) | 0.339 | 0.473 | 29219 | 0.350 | 0.477 | 9074 | 0.334 | 0.472 | 20145 |
| Obese/high-risk obese (>=30) | 0.330 | 0.470 | 29219 | 0.298 | 0.457 | 9074 | 0.345 | 0.475 | 20145 |
| BMI missing | 0.027 | 0.163 | 29219 | 0.019 | 0.137 | 9074 | 0.031 | 0.173 | 20145 |
| IADLs or ADLs: 0 | 0.554 | 0.497 | 29219 | 0.573 | 0.495 | 9074 | 0.545 | 0.498 | 20145 |
| IADLs: 1 | 0.156 | 0.363 | 29219 | 0.145 | 0.352 | 9074 | 0.161 | 0.368 | 20145 |
| ADLs: 1-2 | 0.195 | 0.396 | 29219 | 0.198 | 0.399 | 9074 | 0.194 | 0.395 | 20145 |
| ADLs: 3-4 | 0.065 | 0.246 | 29219 | 0.060 | 0.237 | 9074 | 0.067 | 0.250 | 20145 |
| ADLs: 5-6 | 0.030 | 0.172 | 29219 | 0.025 | 0.157 | 9074 | 0.033 | 0.178 | 20145 |
| Chronic Conditions (except ADRD): 0 | 0.052 | 0.223 | 29219 | 0.000 | 0.000 | 9074 | 0.076 | 0.265 | 20145 |
| Chronic Conditions (except ADRD): 1 | 0.107 | 0.309 | 29219 | 0.041 | 0.199 | 9074 | 0.136 | 0.343 | 20145 |

© 2026 Fu W et al. *JAMA Netw. Open*.

| | | | | | | | | | |
|---|---|---|---|---|---|---|---|---|---|
| Chronic Conditions (except ADRD): 2 | 0.166 | 0.373 | 29219 | 0.105 | 0.307 | 9074 | 0.194 | 0.396 | 20145 |
| Chronic Conditions (except ADRD): 3-4 | 0.368 | 0.482 | 29219 | 0.379 | 0.485 | 9074 | 0.363 | 0.481 | 20145 |
| Chronic Conditions (except ADRD): 5-6 | 0.207 | 0.405 | 29219 | 0.306 | 0.461 | 9074 | 0.163 | 0.369 | 20145 |
| Chronic Conditions (except ADRD): 7-10 | 0.094 | 0.292 | 29219 | 0.160 | 0.367 | 9074 | 0.065 | 0.246 | 20145 |
| Chronic Conditions (except ADRD): 11+ | 0.005 | 0.069 | 29219 | 0.008 | 0.089 | 9074 | 0.003 | 0.058 | 20145 |

Notes: The working sample consists of MA beneficiaries from 2015-2022 MCBS. Beneficiaries with only partial enrollment in an MA plan during the past year at the time of the survey and veterans are excluded. We also exclude beneficiaries with ADRD.

© 2026 Fu W et al. *JAMA Netw. Open*.

**eTable 14. Summary Statistics: Negative Control Group: Osteoarthritis/Arthritis (non-ADRD), Comparison Group: non Osteoarthritis/Arthritis (non ADRD)**

| | (1) Negative Control + Comparison | | | (2) Negative Control | | | (3) Comparison | | |
|---|---|---|---|---|---|---|---|---|---|
| | Mean | SD | N | Mean | SD | N | Mean | SD | N |
| Any Troubles Getting Needed Care | 0.078 | 0.268 | 29172 | 0.091 | 0.288 | 12501 | 0.067 | 0.251 | 16671 |
| Any Medical Financial Burden | 0.182 | 0.386 | 24912 | 0.218 | 0.413 | 10490 | 0.156 | 0.363 | 14422 |
| Satisfaction with Access to Specialist | 0.934 | 0.248 | 27254 | 0.922 | 0.269 | 11917 | 0.944 | 0.230 | 15337 |
| Satisfaction with Quality of Care | 0.952 | 0.214 | 28710 | 0.943 | 0.232 | 12410 | 0.958 | 0.200 | 16300 |
| Comparison | 0.571 | 0.495 | 29219 | 0.000 | 0.000 | 12525 | 1.000 | 0.000 | 16694 |
| Negative Control | 0.429 | 0.495 | 29219 | 1.000 | 0.000 | 12525 | 0.000 | 0.000 | 16694 |
| Age Group <65 | 0.170 | 0.375 | 29219 | 0.159 | 0.366 | 12525 | 0.178 | 0.382 | 16694 |
| Age Group 65 to 75 | 0.352 | 0.477 | 29219 | 0.330 | 0.470 | 12525 | 0.368 | 0.482 | 16694 |
| Age Group 75+ | 0.479 | 0.500 | 29219 | 0.511 | 0.500 | 12525 | 0.454 | 0.498 | 16694 |
| Non-Hispanic white | 0.656 | 0.475 | 29219 | 0.642 | 0.480 | 12525 | 0.666 | 0.472 | 16694 |
| Non-Hispanic black | 0.130 | 0.336 | 29219 | 0.139 | 0.346 | 12525 | 0.122 | 0.327 | 16694 |
| Hispanic | 0.157 | 0.364 | 29219 | 0.163 | 0.369 | 12525 | 0.153 | 0.360 | 16694 |
| Other | 0.058 | 0.233 | 29219 | 0.056 | 0.230 | 12525 | 0.059 | 0.235 | 16694 |
| Male | 0.336 | 0.472 | 29219 | 0.260 | 0.439 | 12525 | 0.393 | 0.488 | 16694 |
| Female | 0.664 | 0.472 | 29219 | 0.740 | 0.439 | 12525 | 0.607 | 0.488 | 16694 |
| Not Married | 0.468 | 0.499 | 29219 | 0.456 | 0.498 | 12525 | 0.476 | 0.499 | 16694 |
| Married | 0.362 | 0.481 | 29219 | 0.325 | 0.469 | 12525 | 0.390 | 0.488 | 16694 |
| Married: missing | 0.170 | 0.376 | 29219 | 0.219 | 0.414 | 12525 | 0.133 | 0.340 | 16694 |
| High school or less | 0.466 | 0.499 | 29219 | 0.468 | 0.499 | 12525 | 0.465 | 0.499 | 16694 |
| High School above | 0.361 | 0.480 | 29219 | 0.311 | 0.463 | 12525 | 0.398 | 0.490 | 16694 |
| Education missing | 0.173 | 0.378 | 29219 | 0.221 | 0.415 | 12525 | 0.137 | 0.344 | 16694 |
| Self-respondent | 0.921 | 0.270 | 29219 | 0.932 | 0.251 | 12525 | 0.912 | 0.283 | 16694 |
| Proxy respondent | 0.079 | 0.270 | 29219 | 0.068 | 0.251 | 12525 | 0.088 | 0.283 | 16694 |
| Household Size: 1 | 0.333 | 0.471 | 29219 | 0.355 | 0.478 | 12525 | 0.317 | 0.465 | 16694 |
| Household Size: 2 | 0.450 | 0.498 | 29219 | 0.438 | 0.496 | 12525 | 0.460 | 0.498 | 16694 |
| Household Size: 3+ | 0.216 | 0.412 | 29219 | 0.208 | 0.406 | 12525 | 0.223 | 0.416 | 16694 |
| Underweight/healthy (<25) | 0.304 | 0.460 | 29219 | 0.262 | 0.440 | 12525 | 0.335 | 0.472 | 16694 |
| Overweight (25-30) | 0.339 | 0.473 | 29219 | 0.329 | 0.470 | 12525 | 0.346 | 0.476 | 16694 |
| Obese/high-risk obese (>=30) | 0.330 | 0.470 | 29219 | 0.383 | 0.486 | 12525 | 0.291 | 0.454 | 16694 |
| BMI missing | 0.027 | 0.163 | 29219 | 0.026 | 0.159 | 12525 | 0.028 | 0.166 | 16694 |
| IADLs or ADLs: 0 | 0.554 | 0.497 | 29219 | 0.447 | 0.497 | 12525 | 0.634 | 0.482 | 16694 |
| IADLs: 1 | 0.156 | 0.363 | 29219 | 0.166 | 0.372 | 12525 | 0.149 | 0.356 | 16694 |
| ADLs: 1-2 | 0.195 | 0.396 | 29219 | 0.256 | 0.437 | 12525 | 0.149 | 0.356 | 16694 |
| ADLs: 3-4 | 0.065 | 0.246 | 29219 | 0.092 | 0.289 | 12525 | 0.045 | 0.206 | 16694 |
| ADLs: 5-6 | 0.030 | 0.172 | 29219 | 0.040 | 0.196 | 12525 | 0.023 | 0.151 | 16694 |
| Chronic Conditions (except ADRD): 0 | 0.052 | 0.223 | 29219 | 0.000 | 0.000 | 12525 | 0.092 | 0.289 | 16694 |
| Chronic Conditions (except ADRD): 1 | 0.107 | 0.309 | 29219 | 0.030 | 0.171 | 12525 | 0.164 | 0.371 | 16694 |

© 2026 Fu W et al. *JAMA Netw. Open*.

| | | | | | | | | | |
|---|---|---|---|---|---|---|---|---|---|
| Chronic Conditions (except ADRD): 2 | 0.166 | 0.373 | 29219 | 0.090 | 0.286 | 12525 | 0.224 | 0.417 | 16694 |
| Chronic Conditions (except ADRD): 3-4 | 0.368 | 0.482 | 29219 | 0.371 | 0.483 | 12525 | 0.365 | 0.482 | 16694 |
| Chronic Conditions (except ADRD): 5-6 | 0.207 | 0.405 | 29219 | 0.322 | 0.467 | 12525 | 0.121 | 0.326 | 16694 |
| Chronic Conditions (except ADRD): 7-10 | 0.094 | 0.292 | 29219 | 0.175 | 0.380 | 12525 | 0.033 | 0.180 | 16694 |
| Chronic Conditions (except ADRD): 11+ | 0.005 | 0.069 | 29219 | 0.011 | 0.105 | 12525 | 0.000 | 0.008 | 16694 |

Notes: The working sample consists of MA beneficiaries from 2015-2022 MCBS. Beneficiaries with only partial enrollment in an MA plan during the past year at the time of the survey and veterans are excluded. We also exclude beneficiaries with ADRD.

© 2026 Fu W et al. *JAMA Netw. Open*.

**eTable 15. Summary Statistics: Negative Control Group: Osteoporosis/soft bones (non-ADRD), Comparison Group: non Osteoporosis/soft bones (non ADRD)**

| | (1) | | | (2) | | | (3) | | |
|---|---|---|---|---|---|---|---|---|---|
| | Negative Control + Comparison | | | Negative Control | | | Comparison | | |
| | Mean | SD | N | Mean | SD | N | Mean | SD | N |
| Any Troubles Getting Needed Care | 0.077 | 0.267 | 29079 | 0.081 | 0.273 | 5974 | 0.077 | 0.266 | 23105 |
| Any Medical Financial Burden | 0.182 | 0.385 | 24830 | 0.187 | 0.390 | 5076 | 0.180 | 0.384 | 19754 |
| Satisfaction with Access to Specialist | 0.934 | 0.248 | 27173 | 0.929 | 0.257 | 5665 | 0.936 | 0.245 | 21508 |
| Satisfaction with Quality of Care | 0.952 | 0.214 | 28622 | 0.946 | 0.226 | 5909 | 0.954 | 0.210 | 22713 |
| Comparison | 0.795 | 0.404 | 29126 | 0.000 | 0.000 | 5982 | 1.000 | 0.000 | 23144 |
| Negative Control | 0.205 | 0.404 | 29126 | 1.000 | 0.000 | 5982 | 0.000 | 0.000 | 23144 |
| Age Group <65 | 0.170 | 0.375 | 29126 | 0.102 | 0.302 | 5982 | 0.187 | 0.390 | 23144 |
| Age Group 65 to 75 | 0.352 | 0.478 | 29126 | 0.335 | 0.472 | 5982 | 0.356 | 0.479 | 23144 |
| Age Group 75+ | 0.479 | 0.500 | 29126 | 0.563 | 0.496 | 5982 | 0.457 | 0.498 | 23144 |
| Non-Hispanic white | 0.656 | 0.475 | 29126 | 0.704 | 0.457 | 5982 | 0.643 | 0.479 | 23144 |
| Non-Hispanic black | 0.130 | 0.336 | 29126 | 0.081 | 0.272 | 5982 | 0.142 | 0.349 | 23144 |
| Hispanic | 0.157 | 0.364 | 29126 | 0.165 | 0.372 | 5982 | 0.155 | 0.362 | 23144 |
| Other | 0.058 | 0.233 | 29126 | 0.050 | 0.218 | 5982 | 0.060 | 0.237 | 23144 |
| Male | 0.336 | 0.472 | 29126 | 0.081 | 0.272 | 5982 | 0.402 | 0.490 | 23144 |
| Female | 0.664 | 0.472 | 29126 | 0.919 | 0.272 | 5982 | 0.598 | 0.490 | 23144 |
| Not Married | 0.468 | 0.499 | 29126 | 0.511 | 0.500 | 5982 | 0.456 | 0.498 | 23144 |
| Married | 0.363 | 0.481 | 29126 | 0.318 | 0.466 | 5982 | 0.374 | 0.484 | 23144 |
| Married: missing | 0.170 | 0.375 | 29126 | 0.171 | 0.377 | 5982 | 0.169 | 0.375 | 23144 |
| High school or less | 0.466 | 0.499 | 29126 | 0.469 | 0.499 | 5982 | 0.466 | 0.499 | 23144 |
| High School above | 0.361 | 0.480 | 29126 | 0.358 | 0.480 | 5982 | 0.362 | 0.480 | 23144 |
| Education missing | 0.173 | 0.378 | 29126 | 0.172 | 0.378 | 5982 | 0.173 | 0.378 | 23144 |
| Self-respondent | 0.921 | 0.270 | 29126 | 0.936 | 0.244 | 5982 | 0.917 | 0.276 | 23144 |
| Proxy respondent | 0.079 | 0.270 | 29126 | 0.064 | 0.244 | 5982 | 0.083 | 0.276 | 23144 |
| Household Size: 1 | 0.333 | 0.471 | 29126 | 0.391 | 0.488 | 5982 | 0.318 | 0.466 | 23144 |
| Household Size: 2 | 0.451 | 0.498 | 29126 | 0.410 | 0.492 | 5982 | 0.461 | 0.499 | 23144 |
| Household Size: 3+ | 0.217 | 0.412 | 29126 | 0.200 | 0.400 | 5982 | 0.221 | 0.415 | 23144 |
| Underweight/healthy (<25) | 0.304 | 0.460 | 29126 | 0.404 | 0.491 | 5982 | 0.278 | 0.448 | 23144 |
| Overweight (25-30) | 0.338 | 0.473 | 29126 | 0.301 | 0.459 | 5982 | 0.348 | 0.476 | 23144 |
| Obese/high-risk obese (>=30) | 0.330 | 0.470 | 29126 | 0.269 | 0.444 | 5982 | 0.346 | 0.476 | 23144 |
| BMI missing | 0.027 | 0.162 | 29126 | 0.025 | 0.156 | 5982 | 0.028 | 0.164 | 23144 |
| IADLs or ADLs: 0 | 0.554 | 0.497 | 29126 | 0.474 | 0.499 | 5982 | 0.575 | 0.494 | 23144 |
| IADLs: 1 | 0.156 | 0.363 | 29126 | 0.172 | 0.377 | 5982 | 0.152 | 0.359 | 23144 |
| ADLs: 1-2 | 0.195 | 0.396 | 29126 | 0.232 | 0.422 | 5982 | 0.185 | 0.389 | 23144 |
| ADLs: 3-4 | 0.065 | 0.246 | 29126 | 0.084 | 0.278 | 5982 | 0.060 | 0.237 | 23144 |
| ADLs: 5-6 | 0.030 | 0.171 | 29126 | 0.038 | 0.191 | 5982 | 0.028 | 0.165 | 23144 |
| Chronic Conditions (except ADRD): 0 | 0.052 | 0.223 | 29126 | 0.000 | 0.000 | 5982 | 0.066 | 0.248 | 23144 |
| Chronic Conditions (except ADRD): 1 | 0.107 | 0.309 | 29126 | 0.030 | 0.169 | 5982 | 0.127 | 0.333 | 23144 |

© 2026 Fu W et al. *JAMA Netw. Open*.

| | | | | | | | | | |
|---|---|---|---|---|---|---|---|---|---|
| Chronic Conditions (except ADRD): 2 | 0.166 | 0.373 | 29126 | 0.093 | 0.290 | 5982 | 0.185 | 0.389 | 23144 |
| Chronic Conditions (except ADRD): 3-4 | 0.368 | 0.482 | 29126 | 0.335 | 0.472 | 5982 | 0.376 | 0.484 | 23144 |
| Chronic Conditions (except ADRD): 5-6 | 0.207 | 0.405 | 29126 | 0.323 | 0.468 | 5982 | 0.177 | 0.382 | 23144 |
| Chronic Conditions (except ADRD): 7-10 | 0.094 | 0.292 | 29126 | 0.202 | 0.402 | 5982 | 0.066 | 0.249 | 23144 |
| Chronic Conditions (except ADRD): 11+ | 0.005 | 0.069 | 29126 | 0.017 | 0.129 | 5982 | 0.002 | 0.040 | 23144 |

Notes: The working sample consists of MA beneficiaries from 2015-2022 MCBS. Beneficiaries with only partial enrollment in an MA plan during the past year at the time of the survey and veterans are excluded. We also exclude beneficiaries with ADRD.

**eTable 16. Summary Statistics: Negative Control Group: Broken hip (non-ADRD), Comparison Group: non Broken hip (non ADRD)**

| | (1) | | | (2) | | | (3) | | |
|---|---|---|---|---|---|---|---|---|---|
| | Negative Control + Comparison | | | Negative Control | | | Comparison | | |
| | Mean | SD | N | Mean | SD | N | Mean | SD | N |
| Any Troubles Getting Needed Care | 0.077 | 0.267 | 29072 | 0.075 | 0.263 | 1094 | 0.078 | 0.267 | 27978 |
| Any Medical Financial Burden | 0.182 | 0.385 | 24824 | 0.224 | 0.417 | 899 | 0.180 | 0.384 | 23925 |
| Satisfaction with Access to Specialist | 0.934 | 0.248 | 27166 | 0.945 | 0.227 | 1025 | 0.934 | 0.248 | 26141 |
| Satisfaction with Quality of Care | 0.952 | 0.214 | 28615 | 0.946 | 0.226 | 1080 | 0.952 | 0.213 | 27535 |
| Comparison | 0.962 | 0.190 | 29119 | 0.000 | 0.000 | 1096 | 1.000 | 0.000 | 28023 |
| Negative Control | 0.038 | 0.190 | 29119 | 1.000 | 0.000 | 1096 | 0.000 | 0.000 | 28023 |
| Age Group <65 | 0.170 | 0.375 | 29119 | 0.140 | 0.347 | 1096 | 0.171 | 0.376 | 28023 |
| Age Group 65 to 75 | 0.352 | 0.478 | 29119 | 0.206 | 0.405 | 1096 | 0.358 | 0.479 | 28023 |
| Age Group 75+ | 0.479 | 0.500 | 29119 | 0.654 | 0.476 | 1096 | 0.472 | 0.499 | 28023 |
| Non-Hispanic white | 0.656 | 0.475 | 29119 | 0.685 | 0.465 | 1096 | 0.655 | 0.476 | 28023 |
| Non-Hispanic black | 0.130 | 0.336 | 29119 | 0.112 | 0.316 | 1096 | 0.130 | 0.337 | 28023 |
| Hispanic | 0.157 | 0.364 | 29119 | 0.153 | 0.360 | 1096 | 0.157 | 0.364 | 28023 |
| Other | 0.058 | 0.233 | 29119 | 0.049 | 0.217 | 1096 | 0.058 | 0.234 | 28023 |
| Male | 0.336 | 0.472 | 29119 | 0.297 | 0.457 | 1096 | 0.338 | 0.473 | 28023 |
| Female | 0.664 | 0.472 | 29119 | 0.703 | 0.457 | 1096 | 0.662 | 0.473 | 28023 |
| Not Married | 0.468 | 0.499 | 29119 | 0.573 | 0.495 | 1096 | 0.463 | 0.499 | 28023 |
| Married | 0.363 | 0.481 | 29119 | 0.279 | 0.449 | 1096 | 0.366 | 0.482 | 28023 |
| Married: missing | 0.170 | 0.375 | 29119 | 0.148 | 0.355 | 1096 | 0.171 | 0.376 | 28023 |
| High school or less | 0.466 | 0.499 | 29119 | 0.523 | 0.500 | 1096 | 0.464 | 0.499 | 28023 |
| High School above | 0.361 | 0.480 | 29119 | 0.329 | 0.470 | 1096 | 0.362 | 0.481 | 28023 |
| Education missing | 0.173 | 0.378 | 29119 | 0.148 | 0.355 | 1096 | 0.174 | 0.379 | 28023 |
| Self-respondent | 0.921 | 0.270 | 29119 | 0.896 | 0.305 | 1096 | 0.922 | 0.269 | 28023 |
| Proxy respondent | 0.079 | 0.270 | 29119 | 0.104 | 0.305 | 1096 | 0.078 | 0.269 | 28023 |
| Household Size: 1 | 0.333 | 0.471 | 29119 | 0.400 | 0.490 | 1096 | 0.330 | 0.470 | 28023 |
| Household Size: 2 | 0.451 | 0.498 | 29119 | 0.404 | 0.491 | 1096 | 0.453 | 0.498 | 28023 |
| Household Size: 3+ | 0.217 | 0.412 | 29119 | 0.196 | 0.397 | 1096 | 0.217 | 0.412 | 28023 |
| Underweight/healthy (<25) | 0.304 | 0.460 | 29119 | 0.446 | 0.497 | 1096 | 0.298 | 0.458 | 28023 |
| Overweight (25-30) | 0.339 | 0.473 | 29119 | 0.302 | 0.459 | 1096 | 0.340 | 0.474 | 28023 |
| Obese/high-risk obese (>=30) | 0.330 | 0.470 | 29119 | 0.227 | 0.419 | 1096 | 0.335 | 0.472 | 28023 |
| BMI missing | 0.027 | 0.162 | 29119 | 0.025 | 0.155 | 1096 | 0.027 | 0.163 | 28023 |
| IADLs or ADLs: 0 | 0.554 | 0.497 | 29119 | 0.347 | 0.476 | 1096 | 0.563 | 0.496 | 28023 |
| IADLs: 1 | 0.156 | 0.363 | 29119 | 0.121 | 0.327 | 1096 | 0.157 | 0.364 | 28023 |
| ADLs: 1-2 | 0.195 | 0.396 | 29119 | 0.306 | 0.461 | 1096 | 0.191 | 0.393 | 28023 |
| ADLs: 3-4 | 0.065 | 0.246 | 29119 | 0.150 | 0.357 | 1096 | 0.061 | 0.240 | 28023 |
| ADLs: 5-6 | 0.030 | 0.171 | 29119 | 0.077 | 0.266 | 1096 | 0.028 | 0.166 | 28023 |
| Chronic Conditions (except ADRD): 0 | 0.052 | 0.223 | 29119 | 0.000 | 0.000 | 1096 | 0.055 | 0.227 | 28023 |
| Chronic Conditions (except ADRD): 1 | 0.107 | 0.309 | 29119 | 0.038 | 0.192 | 1096 | 0.110 | 0.312 | 28023 |

© 2026 Fu W et al. *JAMA Netw. Open*.

| | | | | | | | | | |
|---|---|---|---|---|---|---|---|---|---|
| Chronic Conditions (except ADRD): 2 | 0.166 | 0.372 | 29119 | 0.091 | 0.288 | 1096 | 0.169 | 0.375 | 28023 |
| Chronic Conditions (except ADRD): 3-4 | 0.368 | 0.482 | 29119 | 0.284 | 0.451 | 1096 | 0.371 | 0.483 | 28023 |
| Chronic Conditions (except ADRD): 5-6 | 0.207 | 0.405 | 29119 | 0.314 | 0.464 | 1096 | 0.203 | 0.402 | 28023 |
| Chronic Conditions (except ADRD): 7-10 | 0.094 | 0.292 | 29119 | 0.256 | 0.437 | 1096 | 0.088 | 0.283 | 28023 |
| Chronic Conditions (except ADRD): 11+ | 0.005 | 0.069 | 29119 | 0.016 | 0.127 | 1096 | 0.004 | 0.066 | 28023 |

Notes: The working sample consists of MA beneficiaries from 2015-2022 MCBS. Beneficiaries with only partial enrollment in an MA plan during the past year at the time of the survey and veterans are excluded. We also exclude beneficiaries with ADRD.

© 2026 Fu W et al. *JAMA Netw. Open*.

**eTable 17. Summary Statistics: Negative Control Group: Emphysema/asthma/COPD (non-ADRD), Comparison Group: non Emphysema/asthma/COPD (non ADRD)**

| | (1) Negative Control + Comparison | | | (2) Negative Control | | | (3) Comparison | | |
|---|---|---|---|---|---|---|---|---|---|
| | Mean | SD | N | Mean | SD | N | Mean | SD | N |
| Any Troubles Getting Needed Care | 0.077 | 0.267 | 29052 | 0.108 | 0.310 | 5799 | 0.070 | 0.255 | 23253 |
| Any Medical Financial Burden | 0.181 | 0.385 | 24809 | 0.267 | 0.442 | 4963 | 0.160 | 0.367 | 19846 |
| Satisfaction with Access to Specialist | 0.934 | 0.248 | 27148 | 0.911 | 0.284 | 5558 | 0.940 | 0.237 | 21590 |
| Satisfaction with Quality of Care | 0.952 | 0.214 | 28595 | 0.929 | 0.257 | 5741 | 0.958 | 0.201 | 22854 |
| Comparison | 0.800 | 0.400 | 29099 | 0.000 | 0.000 | 5810 | 1.000 | 0.000 | 23289 |
| Negative Control | 0.200 | 0.400 | 29099 | 1.000 | 0.000 | 5810 | 0.000 | 0.000 | 23289 |
| Age Group <65 | 0.170 | 0.375 | 29099 | 0.254 | 0.435 | 5810 | 0.149 | 0.356 | 23289 |
| Age Group 65 to 75 | 0.352 | 0.478 | 29099 | 0.325 | 0.468 | 5810 | 0.359 | 0.480 | 23289 |
| Age Group 75+ | 0.478 | 0.500 | 29099 | 0.421 | 0.494 | 5810 | 0.493 | 0.500 | 23289 |
| Non-Hispanic white | 0.656 | 0.475 | 29099 | 0.671 | 0.470 | 5810 | 0.652 | 0.476 | 23289 |
| Non-Hispanic black | 0.130 | 0.336 | 29099 | 0.135 | 0.342 | 5810 | 0.128 | 0.335 | 23289 |
| Hispanic | 0.157 | 0.364 | 29099 | 0.130 | 0.336 | 5810 | 0.164 | 0.370 | 23289 |
| Other | 0.058 | 0.233 | 29099 | 0.064 | 0.245 | 5810 | 0.056 | 0.230 | 23289 |
| Male | 0.336 | 0.472 | 29099 | 0.298 | 0.457 | 5810 | 0.346 | 0.476 | 23289 |
| Female | 0.664 | 0.472 | 29099 | 0.702 | 0.457 | 5810 | 0.654 | 0.476 | 23289 |
| Not Married | 0.468 | 0.499 | 29099 | 0.521 | 0.500 | 5810 | 0.454 | 0.498 | 23289 |
| Married | 0.363 | 0.481 | 29099 | 0.307 | 0.461 | 5810 | 0.376 | 0.485 | 23289 |
| Married: missing | 0.170 | 0.375 | 29099 | 0.172 | 0.377 | 5810 | 0.169 | 0.375 | 23289 |
| High school or less | 0.466 | 0.499 | 29099 | 0.506 | 0.500 | 5810 | 0.456 | 0.498 | 23289 |
| High School above | 0.361 | 0.480 | 29099 | 0.320 | 0.467 | 5810 | 0.371 | 0.483 | 23289 |
| Education missing | 0.173 | 0.378 | 29099 | 0.174 | 0.379 | 5810 | 0.172 | 0.378 | 23289 |
| Self-respondent | 0.921 | 0.270 | 29099 | 0.929 | 0.257 | 5810 | 0.919 | 0.273 | 23289 |
| Proxy respondent | 0.079 | 0.270 | 29099 | 0.071 | 0.257 | 5810 | 0.081 | 0.273 | 23289 |
| Household Size: 1 | 0.333 | 0.471 | 29099 | 0.348 | 0.476 | 5810 | 0.329 | 0.470 | 23289 |
| Household Size: 2 | 0.451 | 0.498 | 29099 | 0.413 | 0.492 | 5810 | 0.460 | 0.498 | 23289 |
| Household Size: 3+ | 0.217 | 0.412 | 29099 | 0.239 | 0.426 | 5810 | 0.211 | 0.408 | 23289 |
| Underweight/healthy (<25) | 0.304 | 0.460 | 29099 | 0.278 | 0.448 | 5810 | 0.310 | 0.463 | 23289 |
| Overweight (25-30) | 0.339 | 0.473 | 29099 | 0.285 | 0.451 | 5810 | 0.352 | 0.478 | 23289 |
| Obese/high-risk obese (>=30) | 0.330 | 0.470 | 29099 | 0.412 | 0.492 | 5810 | 0.310 | 0.463 | 23289 |
| BMI missing | 0.027 | 0.162 | 29099 | 0.025 | 0.155 | 5810 | 0.028 | 0.164 | 23289 |
| IADLs or ADLs: 0 | 0.554 | 0.497 | 29099 | 0.388 | 0.487 | 5810 | 0.596 | 0.491 | 23289 |
| IADLs: 1 | 0.156 | 0.363 | 29099 | 0.184 | 0.388 | 5810 | 0.149 | 0.356 | 23289 |
| ADLs: 1-2 | 0.195 | 0.396 | 29099 | 0.278 | 0.448 | 5810 | 0.174 | 0.379 | 23289 |
| ADLs: 3-4 | 0.065 | 0.246 | 29099 | 0.105 | 0.306 | 5810 | 0.055 | 0.227 | 23289 |
| ADLs: 5-6 | 0.030 | 0.171 | 29099 | 0.045 | 0.207 | 5810 | 0.026 | 0.161 | 23289 |
| Chronic Conditions (except ADRD): 0 | 0.052 | 0.223 | 29099 | 0.000 | 0.000 | 5810 | 0.066 | 0.248 | 23289 |
| Chronic Conditions (except ADRD): 1 | 0.107 | 0.309 | 29099 | 0.035 | 0.185 | 5810 | 0.125 | 0.330 | 23289 |

© 2026 Fu W et al. *JAMA Netw. Open*.

| | | | | | | | | | |
|---|---|---|---|---|---|---|---|---|---|
| Chronic Conditions (except ADRD): 2 | 0.166 | 0.372 | 29099 | 0.080 | 0.271 | 5810 | 0.188 | 0.391 | 23289 |
| Chronic Conditions (except ADRD): 3-4 | 0.368 | 0.482 | 29099 | 0.317 | 0.465 | 5810 | 0.380 | 0.486 | 23289 |
| Chronic Conditions (except ADRD): 5-6 | 0.207 | 0.405 | 29099 | 0.323 | 0.467 | 5810 | 0.179 | 0.383 | 23289 |
| Chronic Conditions (except ADRD): 7-10 | 0.094 | 0.292 | 29099 | 0.227 | 0.419 | 5810 | 0.061 | 0.240 | 23289 |
| Chronic Conditions (except ADRD): 11+ | 0.005 | 0.069 | 29099 | 0.018 | 0.132 | 5810 | 0.002 | 0.040 | 23289 |

Notes: The working sample consists of MA beneficiaries from 2015-2022 MCBS. Beneficiaries with only partial enrollment in an MA plan during the past year at the time of the survey and veterans are excluded. We also exclude beneficiaries with ADRD.

© 2026 Fu W et al. *JAMA Netw. Open*.

**eTable 18. Summary Statistics: Negative Control Group: Diabetes/high blood sugar (non-ADRD), Comparison Group: non Diabetes/high blood sugar (non ADRD)**

| | (1) Negative Control + Comparison | | | (2) Negative Control | | | (3) Comparison | | |
|---|---|---|---|---|---|---|---|---|---|
| | Mean | SD | N | Mean | SD | N | Mean | SD | N |
| Any Troubles Getting Needed Care | 0.077 | 0.267 | 29007 | 0.089 | 0.285 | 9899 | 0.072 | 0.258 | 19108 |
| Any Medical Financial Burden | 0.181 | 0.385 | 24770 | 0.226 | 0.418 | 8454 | 0.159 | 0.365 | 16316 |
| Satisfaction with Access to Specialist | 0.934 | 0.247 | 27108 | 0.931 | 0.253 | 9440 | 0.936 | 0.244 | 17668 |
| Satisfaction with Quality of Care | 0.952 | 0.214 | 28552 | 0.948 | 0.222 | 9835 | 0.954 | 0.209 | 18717 |
| Comparison | 0.659 | 0.474 | 29053 | 0.000 | 0.000 | 9913 | 1.000 | 0.000 | 19140 |
| Negative Control | 0.341 | 0.474 | 29053 | 1.000 | 0.000 | 9913 | 0.000 | 0.000 | 19140 |
| Age Group <65 | 0.170 | 0.375 | 29053 | 0.184 | 0.388 | 9913 | 0.162 | 0.369 | 19140 |
| Age Group 65 to 75 | 0.352 | 0.477 | 29053 | 0.369 | 0.483 | 9913 | 0.343 | 0.475 | 19140 |
| Age Group 75+ | 0.479 | 0.500 | 29053 | 0.447 | 0.497 | 9913 | 0.495 | 0.500 | 19140 |
| Non-Hispanic white | 0.656 | 0.475 | 29053 | 0.544 | 0.498 | 9913 | 0.714 | 0.452 | 19140 |
| Non-Hispanic black | 0.130 | 0.336 | 29053 | 0.168 | 0.374 | 9913 | 0.110 | 0.312 | 19140 |
| Hispanic | 0.157 | 0.364 | 29053 | 0.214 | 0.410 | 9913 | 0.127 | 0.333 | 19140 |
| Other | 0.058 | 0.233 | 29053 | 0.074 | 0.261 | 9913 | 0.049 | 0.217 | 19140 |
| Male | 0.336 | 0.472 | 29053 | 0.359 | 0.480 | 9913 | 0.325 | 0.468 | 19140 |
| Female | 0.664 | 0.472 | 29053 | 0.641 | 0.480 | 9913 | 0.675 | 0.468 | 19140 |
| Not Married | 0.467 | 0.499 | 29053 | 0.470 | 0.499 | 9913 | 0.466 | 0.499 | 19140 |
| Married | 0.363 | 0.481 | 29053 | 0.359 | 0.480 | 9913 | 0.365 | 0.481 | 19140 |
| Married: missing | 0.170 | 0.375 | 29053 | 0.171 | 0.377 | 9913 | 0.169 | 0.375 | 19140 |
| High school or less | 0.466 | 0.499 | 29053 | 0.498 | 0.500 | 9913 | 0.450 | 0.497 | 19140 |
| High School above | 0.361 | 0.480 | 29053 | 0.328 | 0.469 | 9913 | 0.378 | 0.485 | 19140 |
| Education missing | 0.173 | 0.378 | 29053 | 0.174 | 0.379 | 9913 | 0.172 | 0.377 | 19140 |
| Self-respondent | 0.921 | 0.270 | 29053 | 0.921 | 0.270 | 9913 | 0.921 | 0.270 | 19140 |
| Proxy respondent | 0.079 | 0.270 | 29053 | 0.079 | 0.270 | 9913 | 0.079 | 0.270 | 19140 |
| Household Size: 1 | 0.332 | 0.471 | 29053 | 0.319 | 0.466 | 9913 | 0.339 | 0.474 | 19140 |
| Household Size: 2 | 0.451 | 0.498 | 29053 | 0.431 | 0.495 | 9913 | 0.461 | 0.498 | 19140 |
| Household Size: 3+ | 0.217 | 0.412 | 29053 | 0.250 | 0.433 | 9913 | 0.200 | 0.400 | 19140 |
| Underweight/healthy (<25) | 0.304 | 0.460 | 29053 | 0.191 | 0.393 | 9913 | 0.363 | 0.481 | 19140 |
| Overweight (25-30) | 0.338 | 0.473 | 29053 | 0.331 | 0.470 | 9913 | 0.342 | 0.474 | 19140 |
| Obese/high-risk obese (>=30) | 0.331 | 0.470 | 29053 | 0.451 | 0.498 | 9913 | 0.269 | 0.443 | 19140 |
| BMI missing | 0.027 | 0.162 | 29053 | 0.028 | 0.165 | 9913 | 0.026 | 0.161 | 19140 |
| IADLs or ADLs: 0 | 0.555 | 0.497 | 29053 | 0.471 | 0.499 | 9913 | 0.598 | 0.490 | 19140 |
| IADLs: 1 | 0.156 | 0.363 | 29053 | 0.165 | 0.371 | 9913 | 0.151 | 0.358 | 19140 |
| ADLs: 1-2 | 0.195 | 0.396 | 29053 | 0.238 | 0.426 | 9913 | 0.173 | 0.378 | 19140 |
| ADLs: 3-4 | 0.065 | 0.246 | 29053 | 0.088 | 0.283 | 9913 | 0.053 | 0.223 | 19140 |
| ADLs: 5-6 | 0.030 | 0.171 | 29053 | 0.039 | 0.194 | 9913 | 0.026 | 0.158 | 19140 |
| Chronic Conditions (except ADRD): 0 | 0.053 | 0.223 | 29053 | 0.000 | 0.000 | 9913 | 0.080 | 0.271 | 19140 |
| Chronic Conditions (except ADRD): 1 | 0.107 | 0.309 | 29053 | 0.018 | 0.134 | 9913 | 0.152 | 0.359 | 19140 |

| | | | | | | | | | |
|---|---|---|---|---|---|---|---|---|---|
| Chronic Conditions (except ADRD): 2 | 0.166 | 0.372 | 29053 | 0.076 | 0.264 | 9913 | 0.213 | 0.409 | 19140 |
| Chronic Conditions (except ADRD): 3-4 | 0.368 | 0.482 | 29053 | 0.391 | 0.488 | 9913 | 0.356 | 0.479 | 19140 |
| Chronic Conditions (except ADRD): 5-6 | 0.207 | 0.405 | 29053 | 0.320 | 0.466 | 9913 | 0.149 | 0.356 | 19140 |
| Chronic Conditions (except ADRD): 7-10 | 0.094 | 0.292 | 29053 | 0.184 | 0.388 | 9913 | 0.048 | 0.213 | 19140 |
| Chronic Conditions (except ADRD): 11+ | 0.005 | 0.069 | 29053 | 0.011 | 0.104 | 9913 | 0.002 | 0.040 | 19140 |

Notes: The working sample consists of MA beneficiaries from 2015-2022 MCBS. Beneficiaries with only partial enrollment in an MA plan during the past year at the time of the survey and veterans are excluded. We also exclude beneficiaries with ADRD.

© 2026 Fu W et al. *JAMA Netw. Open*.

**eTable 19. Summary Statistics: Using FFS Beneficiaries**

| | (1) | | | (2) | | | (3) | | |
|---|---|---|---|---|---|---|---|---|---|
| | All | | | ADRD | | | Stroke/Paralysis/ Parkinson's Disease | | |
| | Mean | SD | N | Mean | SD | N | Mean | SD | N |
| Any Troubles Getting Needed Care | 0.102 | 0.303 | 7177 | 0.085 | 0.279 | 1921 | 0.109 | 0.311 | 5256 |
| Any Medical Financial Burden | 0.235 | 0.424 | 5118 | 0.184 | 0.388 | 1350 | 0.253 | 0.435 | 3768 |
| Satisfaction with Access to Specialist | 0.908 | 0.289 | 6746 | 0.918 | 0.275 | 1785 | 0.904 | 0.294 | 4961 |
| Satisfaction with Quality of Care | 0.935 | 0.246 | 7062 | 0.940 | 0.237 | 1894 | 0.933 | 0.250 | 5168 |
| No ADRD | 0.732 | 0.443 | 7206 | 0.000 | 0.000 | 1931 | 1.000 | 0.000 | 5275 |
| ADRD | 0.268 | 0.443 | 7206 | 1.000 | 0.000 | 1931 | 0.000 | 0.000 | 5275 |
| Age Group <65 | 0.233 | 0.423 | 7206 | 0.094 | 0.292 | 1931 | 0.284 | 0.451 | 5275 |
| Age Group 65 to 75 | 0.222 | 0.416 | 7206 | 0.135 | 0.342 | 1931 | 0.254 | 0.435 | 5275 |
| Age Group 75+ | 0.545 | 0.498 | 7206 | 0.771 | 0.421 | 1931 | 0.462 | 0.499 | 5275 |
| Non-Hispanic white | 0.742 | 0.438 | 7206 | 0.692 | 0.462 | 1931 | 0.760 | 0.427 | 5275 |
| Non-Hispanic black | 0.099 | 0.299 | 7206 | 0.097 | 0.296 | 1931 | 0.100 | 0.300 | 5275 |
| Hispanic | 0.093 | 0.290 | 7206 | 0.133 | 0.340 | 1931 | 0.078 | 0.268 | 5275 |
| Other | 0.066 | 0.248 | 7206 | 0.078 | 0.268 | 1931 | 0.062 | 0.241 | 5275 |
| Male | 0.338 | 0.473 | 7206 | 0.242 | 0.428 | 1931 | 0.373 | 0.484 | 5275 |
| Female | 0.662 | 0.473 | 7206 | 0.758 | 0.428 | 1931 | 0.627 | 0.484 | 5275 |
| Not Married | 0.553 | 0.497 | 7206 | 0.579 | 0.494 | 1931 | 0.543 | 0.498 | 5275 |
| Married | 0.353 | 0.478 | 7206 | 0.335 | 0.472 | 1931 | 0.359 | 0.480 | 5275 |
| Married: missing | 0.094 | 0.292 | 7206 | 0.086 | 0.280 | 1931 | 0.097 | 0.297 | 5275 |
| High school or less | 0.524 | 0.499 | 7206 | 0.597 | 0.491 | 1931 | 0.498 | 0.500 | 5275 |
| High School above | 0.375 | 0.484 | 7206 | 0.303 | 0.460 | 1931 | 0.401 | 0.490 | 5275 |
| Education missing | 0.101 | 0.301 | 7206 | 0.099 | 0.299 | 1931 | 0.101 | 0.301 | 5275 |
| Self-respondent | 0.721 | 0.449 | 7206 | 0.379 | 0.485 | 1931 | 0.846 | 0.361 | 5275 |
| Proxy respondent | 0.279 | 0.449 | 7206 | 0.621 | 0.485 | 1931 | 0.154 | 0.361 | 5275 |
| Household Size: 1 | 0.309 | 0.462 | 7206 | 0.245 | 0.430 | 1931 | 0.333 | 0.471 | 5275 |
| Household Size: 2 | 0.438 | 0.496 | 7206 | 0.455 | 0.498 | 1931 | 0.432 | 0.495 | 5275 |
| Household Size: 3+ | 0.252 | 0.434 | 7206 | 0.299 | 0.458 | 1931 | 0.235 | 0.424 | 5275 |
| Underweight/healthy (<25) | 0.367 | 0.482 | 7206 | 0.455 | 0.498 | 1931 | 0.335 | 0.472 | 5275 |
| Overweight (25-30) | 0.304 | 0.460 | 7206 | 0.277 | 0.448 | 1931 | 0.314 | 0.464 | 5275 |
| Obese/high-risk obese (>=30) | 0.291 | 0.454 | 7206 | 0.215 | 0.411 | 1931 | 0.318 | 0.466 | 5275 |
| BMI missing | 0.038 | 0.192 | 7206 | 0.053 | 0.224 | 1931 | 0.033 | 0.179 | 5275 |
| IADLs or ADLs: 0 | 0.255 | 0.436 | 7206 | 0.144 | 0.352 | 1931 | 0.295 | 0.456 | 5275 |
| IADLs: 1 | 0.167 | 0.373 | 7206 | 0.177 | 0.381 | 1931 | 0.164 | 0.370 | 5275 |
| ADLs: 1-2 | 0.283 | 0.450 | 7206 | 0.251 | 0.434 | 1931 | 0.294 | 0.456 | 5275 |
| ADLs: 3-4 | 0.141 | 0.348 | 7206 | 0.169 | 0.375 | 1931 | 0.131 | 0.337 | 5275 |
| ADLs: 5-6 | 0.155 | 0.362 | 7206 | 0.259 | 0.438 | 1931 | 0.116 | 0.321 | 5275 |
| Chronic Conditions (except ADRD): 0 | 0.009 | 0.093 | 7206 | 0.033 | 0.178 | 1931 | 0.000 | 0.000 | 5275 |
| Chronic Conditions (except ADRD): 1 | 0.055 | 0.228 | 7206 | 0.071 | 0.257 | 1931 | 0.049 | 0.216 | 5275 |
| Chronic Conditions (except ADRD): 2 | 0.092 | 0.289 | 7206 | 0.123 | 0.328 | 1931 | 0.081 | 0.272 | 5275 |

| | | | | | | | | | |
|---|---|---|---|---|---|---|---|---|---|
| Chronic Conditions (except ADRD): 3-4 | 0.296 | 0.456 | 7206 | 0.317 | 0.466 | 1931 | 0.288 | 0.453 | 5275 |
| Chronic Conditions (except ADRD): 5-6 | 0.287 | 0.452 | 7206 | 0.256 | 0.437 | 1931 | 0.298 | 0.458 | 5275 |
| Chronic Conditions (except ADRD): 7-10 | 0.239 | 0.426 | 7206 | 0.182 | 0.386 | 1931 | 0.259 | 0.438 | 5275 |
| Chronic Conditions (except ADRD): 11+ | 0.023 | 0.150 | 7206 | 0.018 | 0.132 | 1931 | 0.025 | 0.156 | 5275 |

Notes: The working sample consists of MA beneficiaries from 2015-2022 MCBS. The sample includes beneficiaries who enroll in a FFS plan.

© 2026 Fu W et al. *JAMA Netw. Open*.

**eTable 20. Dynamic Association between the Inclusion of ADRD HCCs in Payment Model and Care Experiences (Event Study)**

| variable | Coef. | Standard Error | P value | 95% CI: lower | 95% CI: upper |
|---|---|---|---|---|---|
| **Outcome: Any Trouble Getting Needed Care (N = 5,339)** | | | | | |
| ADRD × 2015 | -0.0446 | 0.0448 | 0.3199 | -0.1325 | -0.0433 |
| ADRD × 2016 | -0.0156 | 0.0421 | 0.7100 | -0.0981 | 0.0668 |
| ADRD × 2017 | 0.0029 | 0.0460 | 0.9492 | -0.0872 | 0.0930 |
| ADRD × 2018 | -0.0221 | 0.0438 | 0.6148 | -0.1080 | 0.0639 |
| ADRD × 2020 | -0.0481 | 0.0411 | 0.2422 | -0.1288 | 0.0325 |
| ADRD × 2021 | -0.0989 | 0.0421 | 0.0188 | -0.1814 | -0.0164 |
| ADRD × 2022 | -0.0909 | 0.0471 | 0.0536 | -0.1833 | 0.0014 |
| **Outcome: Any Medical Financial Burden (N = 4,172)** | | | | | |
| ADRD × 2017 | -0.0639 | 0.0636 | 0.3148 | -0.1886 | 0.0608 |
| ADRD × 2018 | 0.0193 | 0.0624 | 0.7573 | -0.1031 | 0.1417 |
| ADRD × 2020 | -0.0974 | 0.0584 | 0.0953 | -0.2118 | 0.0171 |
| ADRD × 2021 | -0.1034 | 0.0565 | 0.0672 | -0.2141 | 0.0073 |
| ADRD × 2022 | -0.1174 | 0.0609 | 0.0542 | -0.2368 | 0.0021 |
| **Outcome: Satisfaction with Specialists Access (N = 5,049)** | | | | | |
| ADRD × 2015 | -0.0301 | 0.0482 | 0.5319 | -0.1246 | 0.0644 |
| ADRD × 2016 | -0.0012 | 0.0446 | 0.9784 | -0.0886 | 0.0862 |
| ADRD × 2017 | 0.0094 | 0.0446 | 0.8323 | -0.0780 | 0.0969 |
| ADRD × 2018 | 0.0261 | 0.0411 | 0.5260 | -0.0545 | 0.1067 |
| ADRD × 2020 | 0.0119 | 0.0410 | 0.7711 | -0.0684 | 0.0923 |
| ADRD × 2021 | 0.0637 | 0.0419 | 0.1283 | -0.0184 | 0.1459 |
| ADRD × 2022 | 0.0526 | 0.0462 | 0.2548 | -0.0380 | 0.1432 |
| **Outcome: Satisfaction with Quality of Care (N = 5,280)** | | | | | |
| ADRD × 2015 | -0.0346 | 0.0374 | 0.3554 | -0.1079 | 0.0388 |
| ADRD × 2016 | -0.0039 | 0.0354 | 0.9123 | -0.0734 | 0.0656 |
| ADRD × 2017 | -0.0281 | 0.0394 | 0.4766 | -0.1053 | 0.0492 |
| ADRD × 2018 | -0.0014 | 0.0344 | 0.9678 | -0.0687 | 0.0660 |
| ADRD × 2020 | -0.0351 | 0.0382 | 0.3587 | -0.1100 | 0.0398 |
| ADRD × 2021 | -0.0295 | 0.0380 | 0.4368 | -0.1040 | 0.0449 |
| ADRD × 2022 | -0.0103 | 0.0430 | 0.8102 | -0.0946 | 0.0740 |

*Notes:* The working sample consists of MA beneficiaries from 2015-2022 MCBS. Beneficiaries with only partial enrollment in an MA plan during the past year at the time of the survey and veteran are excluded. In each estimation, the treatment group consists of MA beneficiaries with ADRD, and the control group consists of MA beneficiaries without ADRD but with stroke/brain hemorrhage, complete/partial paralysis, or Parkinson's diseases. The event study is specified in Appendix B.3. The year of 2019 is selected as the reference year. Robust standard errors are applied.

© 2026 Fu W et al. *JAMA Netw. Open*.

**eFigure 1. Association between the Inclusion of ADRD HCCs in Payment Model and Care Experiences – Exclude MA ADRD Beneficiaries with Self-Response**

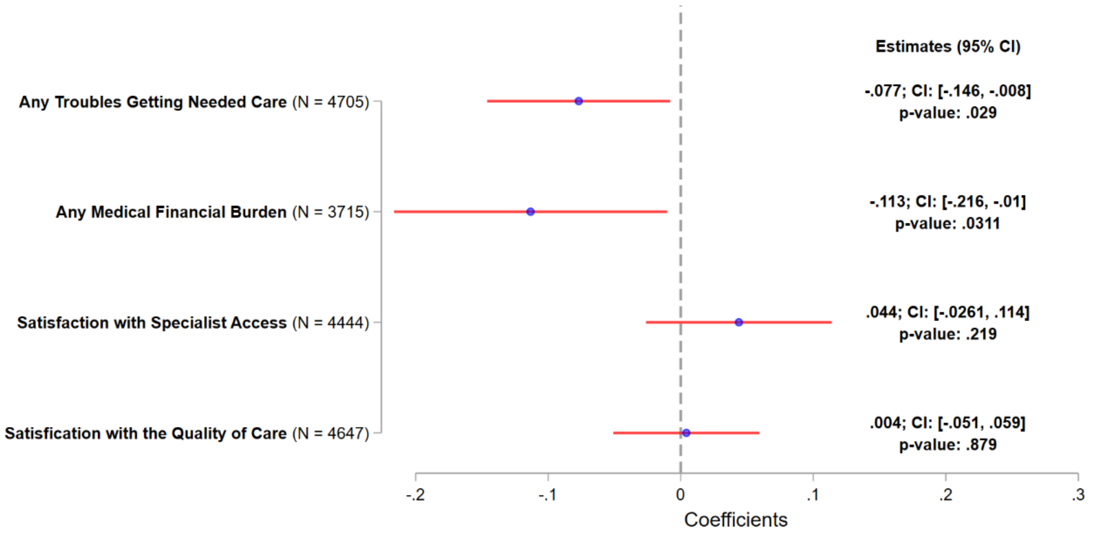


*Notes:* The working sample consists of MA beneficiaries from 2015-2022 MCBS. Beneficiaries with only partial enrollment in an MA plan during the past year at the time of the survey and veteran are excluded. MA ADRD beneficiaries with self-response in the survey are also removed. The plotted estimates represent the coefficients for interaction term between the treatment indicator and the post indicator from a DID estimation, with the dependent variable indicated on the y-axis. In each DID estimation, the treatment group consists of MA beneficiaries with ADRD, and the control group consists of MA beneficiaries without ADRD but with stroke/brain hemorrhage, complete/partial paralysis, or Parkinson's diseases. The post indicator takes the value of one if 2020 or afterwards, and zero otherwise. The associated 95% CIs are plotted. Robust standard errors are applied.

© 2026 Fu W et al. *JAMA Netw. Open*.

**eFigure 2. Association between the Inclusion of ADRD HCCs in Payment Model and Care Experiences – Exclude MA Beneficiaries Under 65**

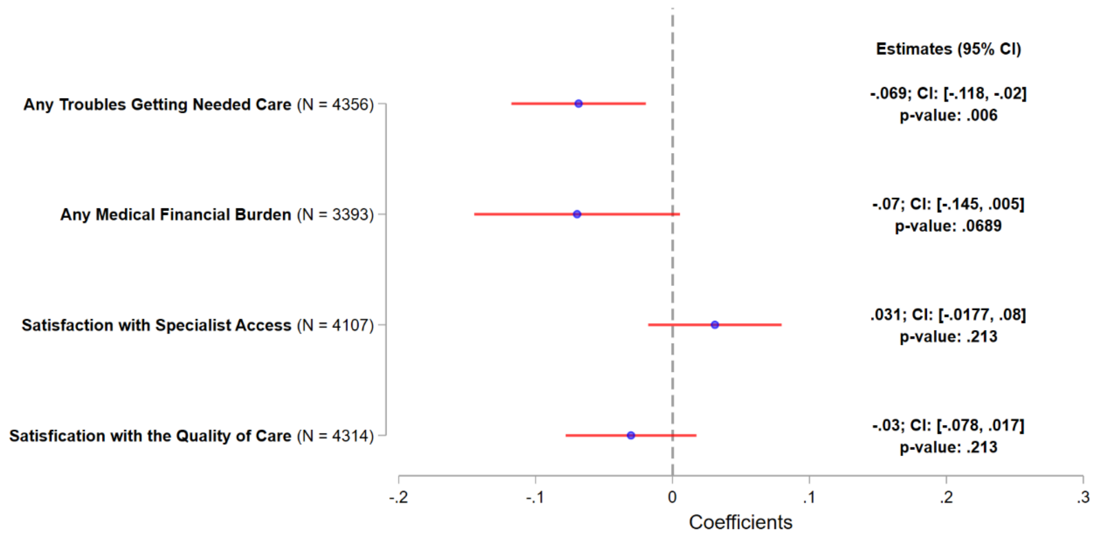


*Notes:* The working sample consists of MA beneficiaries from 2015-2022 MCBS. Beneficiaries with only partial enrollment in an MA plan during the past year at the time of the survey and veteran are excluded. MA beneficiaries less than 65 years old at the time of the survey are also removed. The plotted estimates represent the coefficients for the interaction term between the treatment indicator and the post indicator from a DID estimation, with the dependent variable indicated on the y-axis. In each DID estimation, the treated group consists of MA beneficiaries with ADRD, and the control group consists of MA beneficiaries without ADRD but with stroke/brain hemorrhage, complete/partial paralysis, or Parkinson's diseases. The post indicator takes the value of one if 2020 or afterwards, and zero otherwise. The associated 95% CIs are plotted. Robust standard errors are applied.

© 2026 Fu W et al. *JAMA Netw. Open*.

**eFigure 3. Association between the Inclusion of ADRD HCCs in Payment Model and Beneficiaries' Characteristics – Adjust for Dual-Eligible Status**

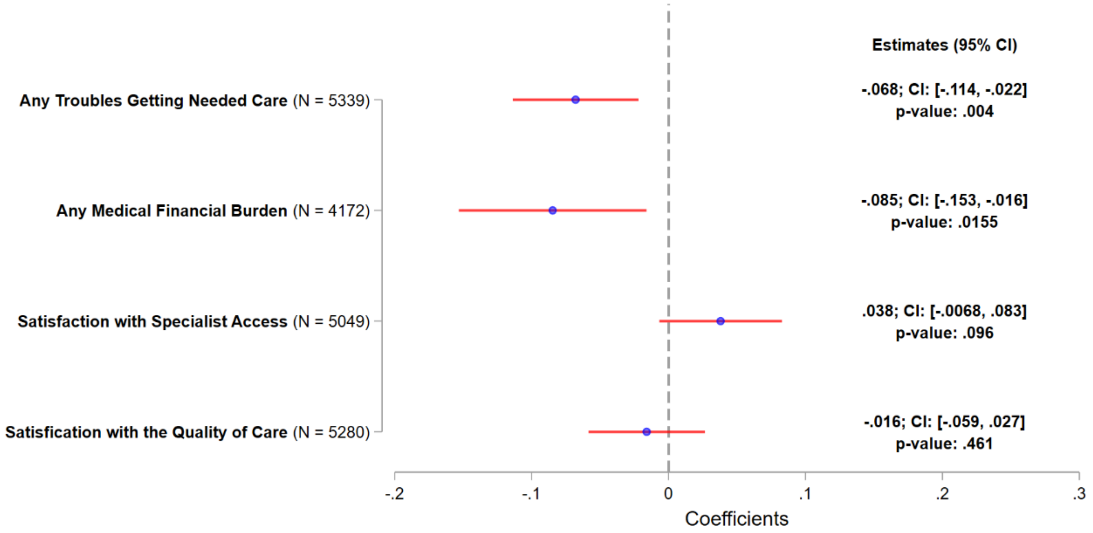


*Notes:* The working sample consists of MA beneficiaries from 2015-2022 MCBS. Beneficiaries with only partial enrollment in an MA plan during the past year at the time of the survey and veteran are excluded. The plotted estimates represent the coefficients for the interaction term between the treatment indicator and the post indicator from a DID estimation, with the dependent variable indicated on the y-axis. In each DID estimation, the treatment group consists of MA beneficiaries with ADRD, and the control group consists of MA beneficiaries without ADRD but with stroke/brain hemorrhage, complete/partial paralysis, or Parkinson's diseases. The post indicator takes the value of one if 2020 or afterwards, and zero otherwise. The associated 95% CIs are plotted. Robust standard errors are applied.

© 2026 Fu W et al. *JAMA Netw. Open*.

**eFigure 4. Association between the Inclusion of ADRD HCCs in Payment Model and Care Experiences using Balanced Repeated Replications Method**

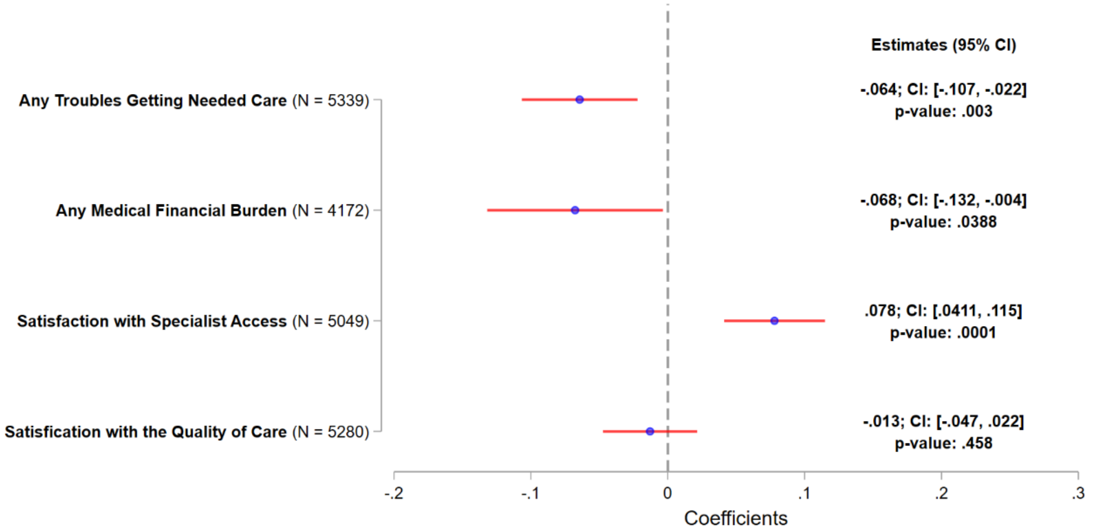


*Notes:* The working sample consists of MA beneficiaries from 2015-2022 MCBS. Beneficiaries with only partial enrollment in an MA plan during the past year at the time of the survey and veteran are excluded. The plotted estimates represent the coefficients for the interaction term between the treatment indicator and the post indicator from a DID estimation, with the dependent variable indicated on the y-axis. In each DID estimation, the treatment group consists of MA beneficiaries with ADRD, and the control group consists of MA beneficiaries without ADRD but with stroke/brain hemorrhage, complete/partial paralysis, or Parkinson's diseases. The post indicator takes the value of one if 2020 or afterwards, and zero otherwise. The associated 95% CIs are plotted. Balanced repeated replications (BRR) for standard errors are applied.

© 2026 Fu W et al. *JAMA Netw. Open*.

**eFigure 5. Association between the Inclusion of ADRD HCCs in Payment Model and Care Experiences – Exclude Beneficiaries in Current MA Plan for Less than 3 Years**

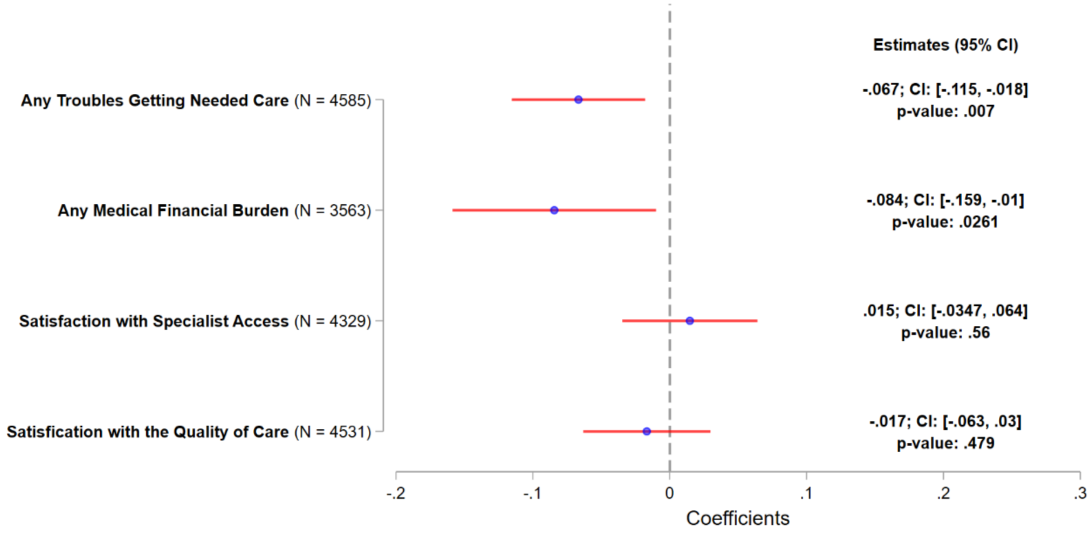


*Notes:* The working sample consists of MA beneficiaries from 2015-2022 MCBS. Beneficiaries with only partial enrollment in an MA plan during the past year at the time of the survey and veteran are excluded. MA beneficiaries who have been in current MA plans for less than 3 years at the time of the survey are also removed. The plotted estimates represent the coefficients for the interaction term between the treatment indicator and the post indicator from a DID estimation, with the dependent variable indicated on the y-axis. In each DID estimation, the treatment group consists of MA beneficiaries with ADRD, and the control group consists of MA beneficiaries without ADRD but with stroke/brain hemorrhage, complete/partial paralysis, or Parkinson’s diseases. The post indicator takes the value of one if 2020 or afterwards, and zero otherwise. The associated 95% CIs are plotted. Robust standard errors are applied.

© 2026 Fu W et al. *JAMA Netw. Open*.

**eFigure 6. Association between the Inclusion of ADRD HCCs in Payment Model and Beneficiaries' Characteristics – Including Partially Covered MA Beneficiaries**

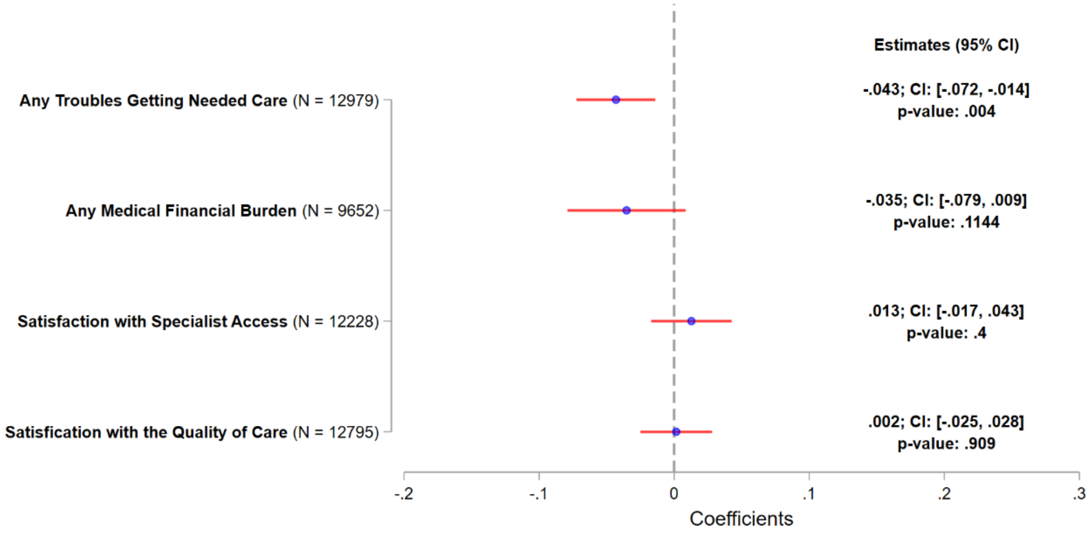


*Notes:* The working sample consists of MA beneficiaries from 2015-2022 MCBS. Beneficiaries with full-year MA coverage or with MA coverage for less than one year during the past year at the time of the survey are both included. Veterans are excluded. The plotted estimates represent the coefficients for the interaction term between the treatment indicator and the post indicator from a DID estimation, with the dependent variable indicated on the y-axis. In each DID estimation, the treatment group consists of MA beneficiaries with ADRD, and the control group consists of MA beneficiaries without ADRD but with stroke/brain hemorrhage, complete/partial paralysis, or Parkinson's diseases. The post indicator takes the value of one if 2020 or afterwards, and zero otherwise. The associated 95% CIs are plotted. Robust standard errors are applied.

© 2026 Fu W et al. *JAMA Netw. Open*.

**eFigure 7. Association between the Inclusion of ADRD HCCs in Payment Model and Beneficiaries' Characteristics – Sensitivity Test to Alternative Control Group**

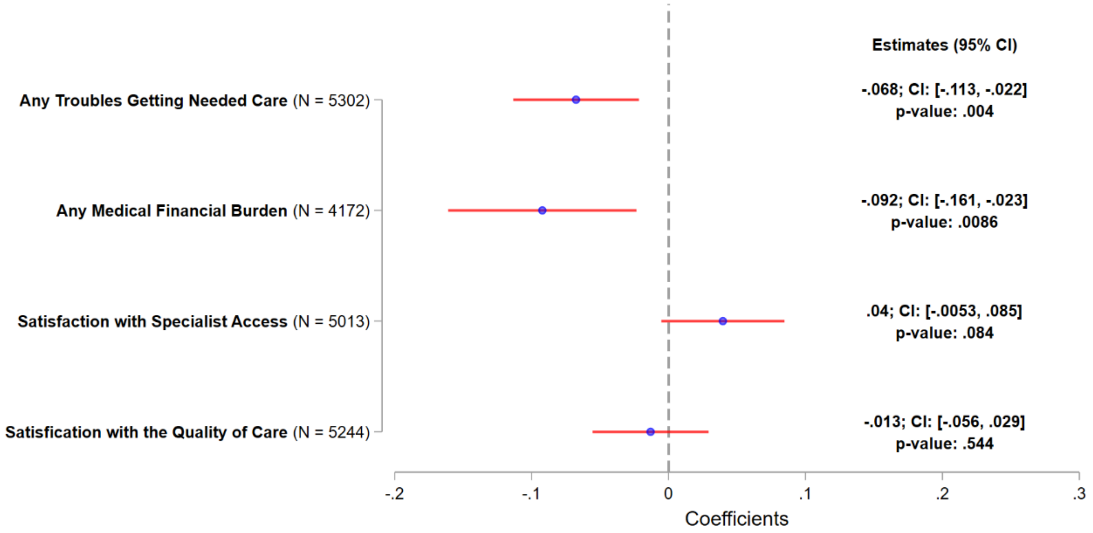


A. Dropping PD from Control Group

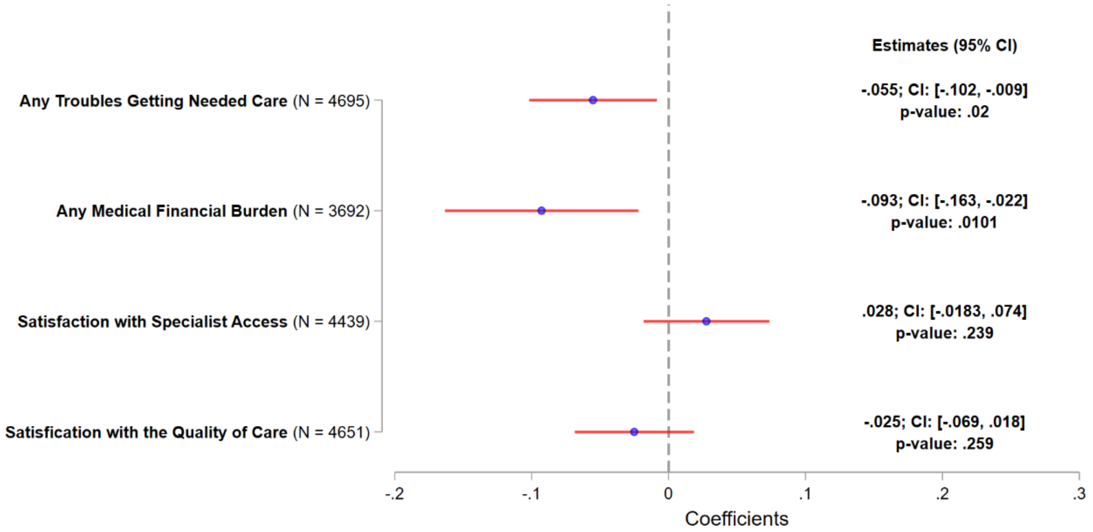


B. Using MA Beneficiaries with Stroke/brain hemorrhage as Control Group

© 2026 Fu W et al. *JAMA Netw. Open*.

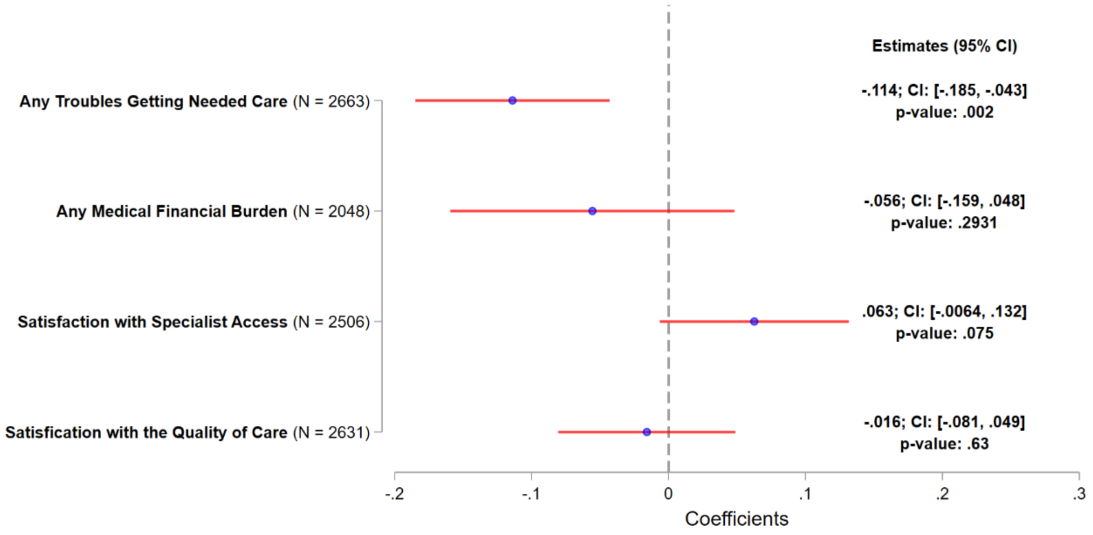


C. Using MA Beneficiaries with Complete/Partial paralysis as Control Group

*Notes:* The working sample consists of MA beneficiaries from 2015-2022 MCBS. Beneficiaries with only partial enrollment in an MA plan during the past year at the time of the survey and veteran are excluded. The plotted estimates represent the coefficients for the interaction term between the treatment indicator and the post indicator from a DID estimation, with the dependent variable indicated on the y-axis. In each DID estimation, the treatment group consists of MA beneficiaries with ADRD. The control group consists of MA beneficiaries without ADRD but with stroke/brain hemorrhage, or complete/partial paralysis in Panel A, MA beneficiaries without ADRD but with stroke/brain hemorrhage in Panel B, and MA beneficiaries without ADRD but with complete/partial paralysis in Panel C. The post indicator takes the value of one if 2020 or afterwards, and zero otherwise. The associated 95% CIs are plotted. Robust standard errors are applied.

**eFigure 8. Association between the Inclusion of ADRD HCCs in Payment Model and Care Experiences – Using MA Beneficiaries with All non-ADRD Conditions as Control Group**

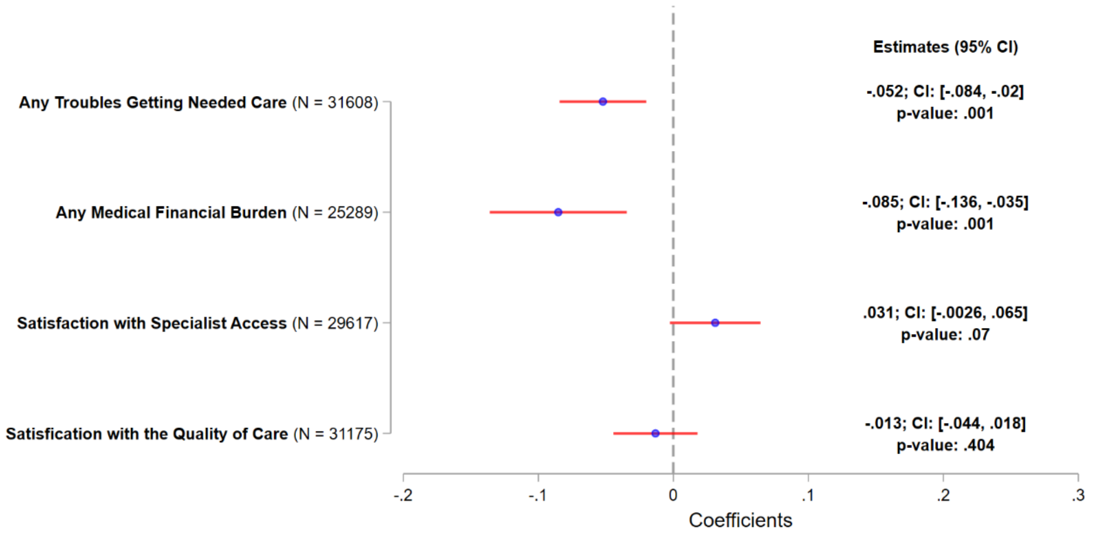


*Notes:* The working sample consists of MA beneficiaries from 2015-2022 MCBS. Beneficiaries with only partial enrollment in an MA plan during the past year at the time of the survey and veteran are excluded. MA ADRD beneficiaries with self-response in the survey are also removed. The plotted estimates represent the coefficients for the interaction term between the treatment indicator and the post indicator from a DID estimation, with the dependent variable indicated on the y-axis. In each DID estimation, the treatment group consists of MA beneficiaries with ADRD, and the control group consists of MA beneficiaries without ADRD but with other conditions, including but not limited to stroke/brain hemorrhage, complete/partial paralysis, or Parkinson's diseases. The post indicator takes the value of one if 2020 or afterwards, and zero otherwise. The associated 95% CIs are plotted. Robust standard errors are applied.

© 2026 Fu W et al. *JAMA Netw. Open*.

## eFigure 9. Placebo Tests Using Other Conditions as Negative Control Group

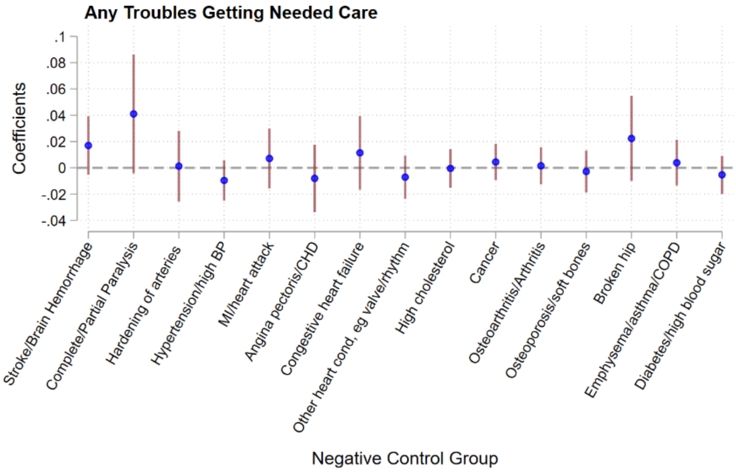


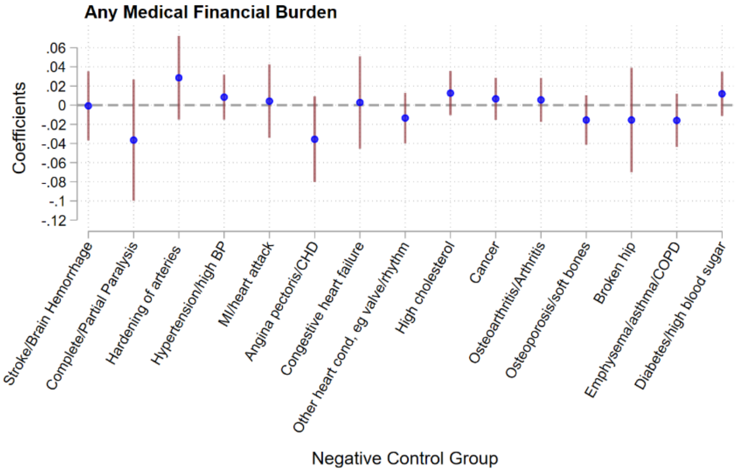


© 2026 Fu W et al. *JAMA Netw. Open*.

**eFigure 9 (Cont.). Placebo Tests Using Other Conditions as Negative Control Group**

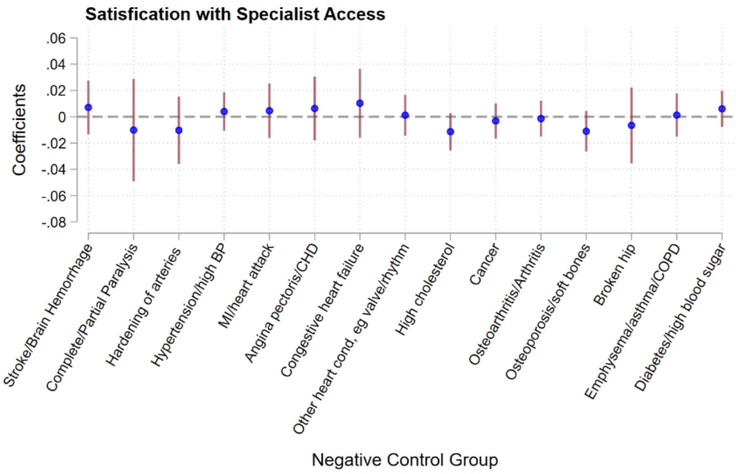


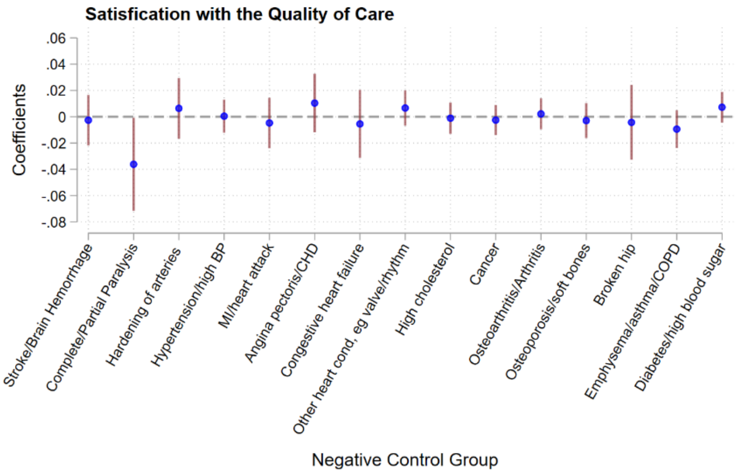


*Notes:* The working sample consists of MA beneficiaries from 2015-2022 MCBS. Beneficiaries with only partial enrollment in an MA plan during the past year at the time of the survey and veteran are excluded. The plotted estimates represent the coefficients for the interaction term between the negative control indicator (listed in the horizontal axis) and the post indicator from a DID estimation, with the dependent variable indicated in each panel title. In each DID estimation, the negative control group consists of non-ADRD MA beneficiaries with the disease specified in the x-axis, and the comparison group consists of non-ADRD MA beneficiaries without the disease specified in the x-axis. The post indicator takes the value of one if 2020 or afterwards, and zero otherwise. The associated 95% CIs are plotted. Robust standard errors are applied.

**eFigure 10. Association between the Inclusion of ADRD HCCs in Payment Model and Care Experiences – Using Fee-For-Service (FFS) Beneficiaries**

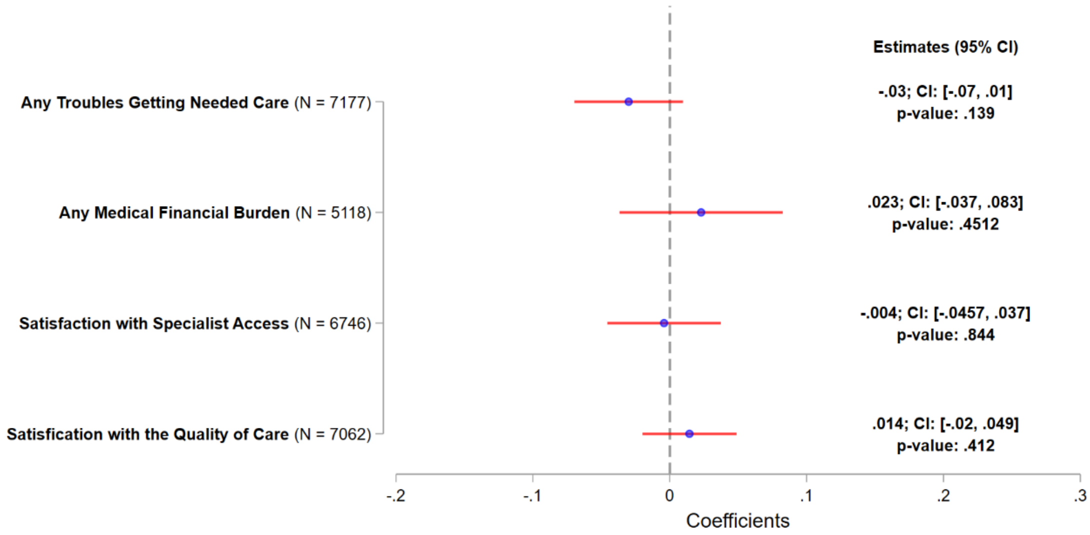


*Notes:* The working sample consists of TM beneficiaries from 2015-2022 MCBS. Veteran beneficiaries are excluded. The plotted estimates represent the coefficients for the interaction term between the treatment indicator and the post indicator from a DID estimation, with the dependent variable indicated on the y-axis. In each DID estimation, the treatment group consists of FFS beneficiaries with ADRD, and the control group consists of FFS beneficiaries without ADRD but with stroke/brain hemorrhage, complete/partial paralysis, or Parkinson's diseases. The post indicator takes the value of one if 2020 or afterwards, and zero otherwise. The associated 95% CIs are plotted. Robust standard errors are applied.

**eFigure 11. Association between the Inclusion of ADRD HCCs in Payment Model and Care Experiences – Stratification Analysis**

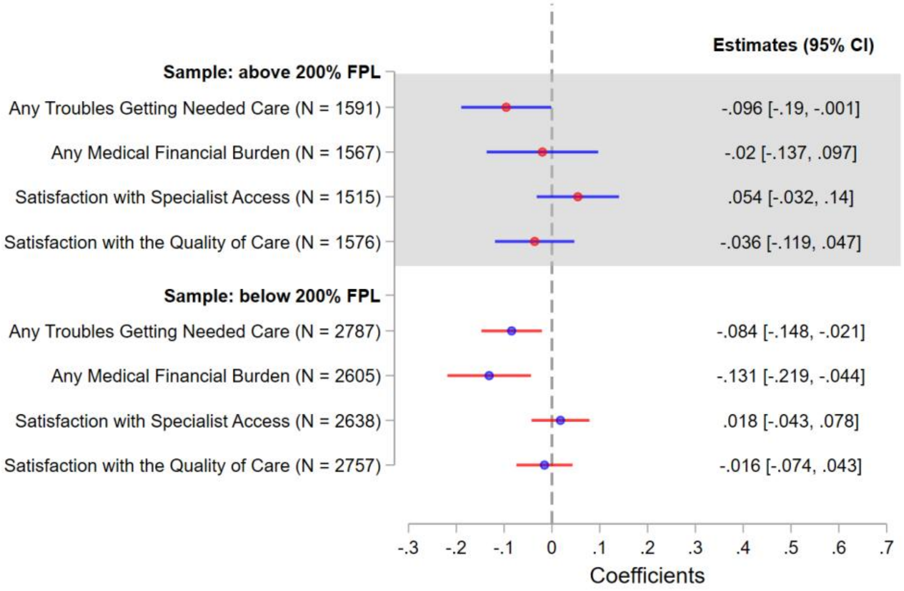


A. By Income Level

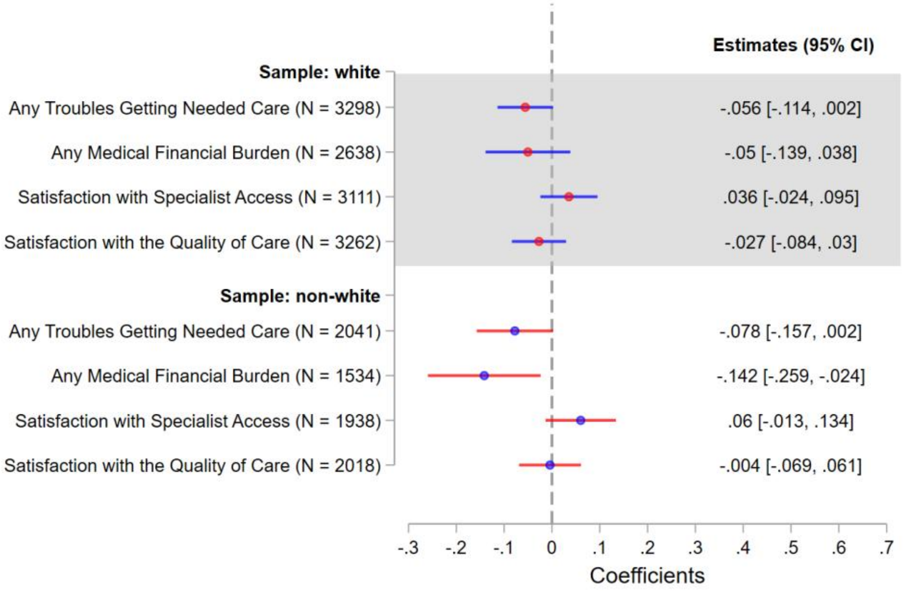


B. By Race/Ethnicity

© 2026 Fu W et al. *JAMA Netw. Open*.

**eFigure 11. (Cont.) Association between the Inclusion of ADRD HCCs in Payment Model and Care Experiences – Stratification Analysis**

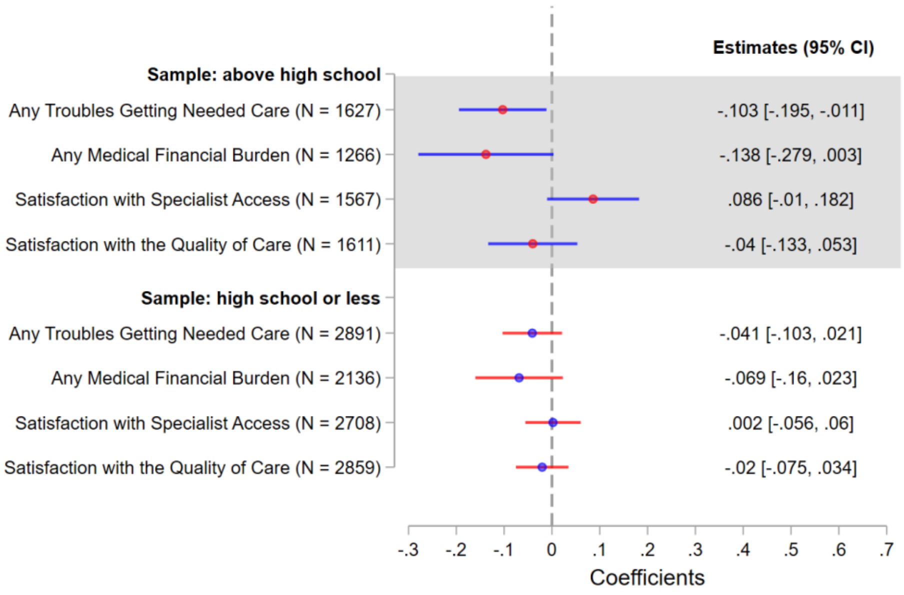


C. By Education Level

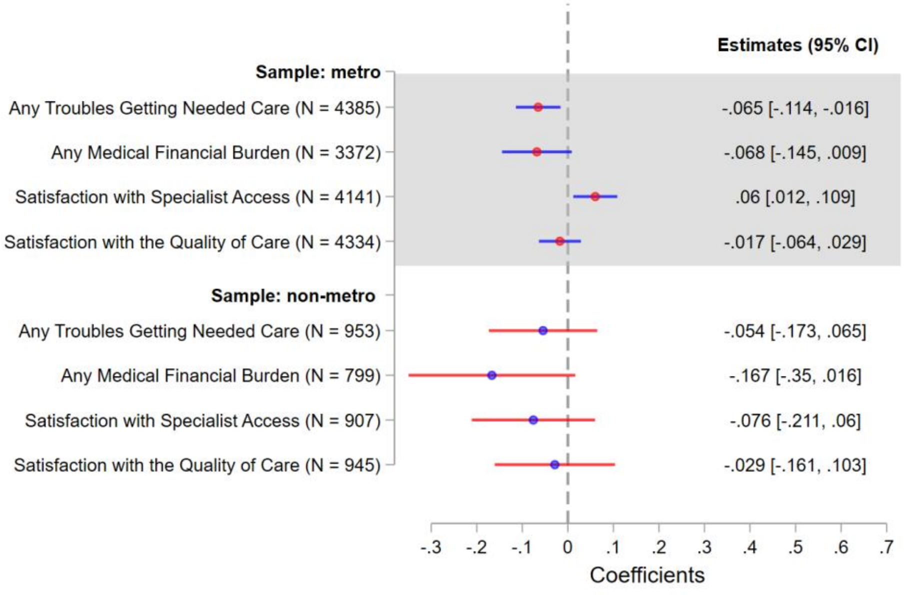


D. By Metropolitan Status

*Notes:* The working sample consists of MA beneficiaries from 2015-2022 MCBS. Beneficiaries with only partial enrollment in an MA plan during the past year at the time of the survey and veteran are excluded. The plotted estimates represent the coefficients for the interaction term between the treatment indicator and the post indicator from a DID estimation, with the dependent variable indicated on the y-axis. In each DID estimation, the treatment group consists of MA beneficiaries with ADRD, and the control group consists of MA beneficiaries without ADRD but with stroke/brain hemorrhage,

© 2026 Fu W et al. *JAMA Netw. Open*.

complete/partial paralysis, or Parkinson's diseases. The post indicator takes the value of one if 2020 or afterwards, and zero otherwise. The associated 95% CIs are plotted. Robust standard errors are applied.

**eFigure 12. Association between the Inclusion of ADRD HCCs in Payment Model and Beneficiaries' Characteristics – Test for Compositional Change**

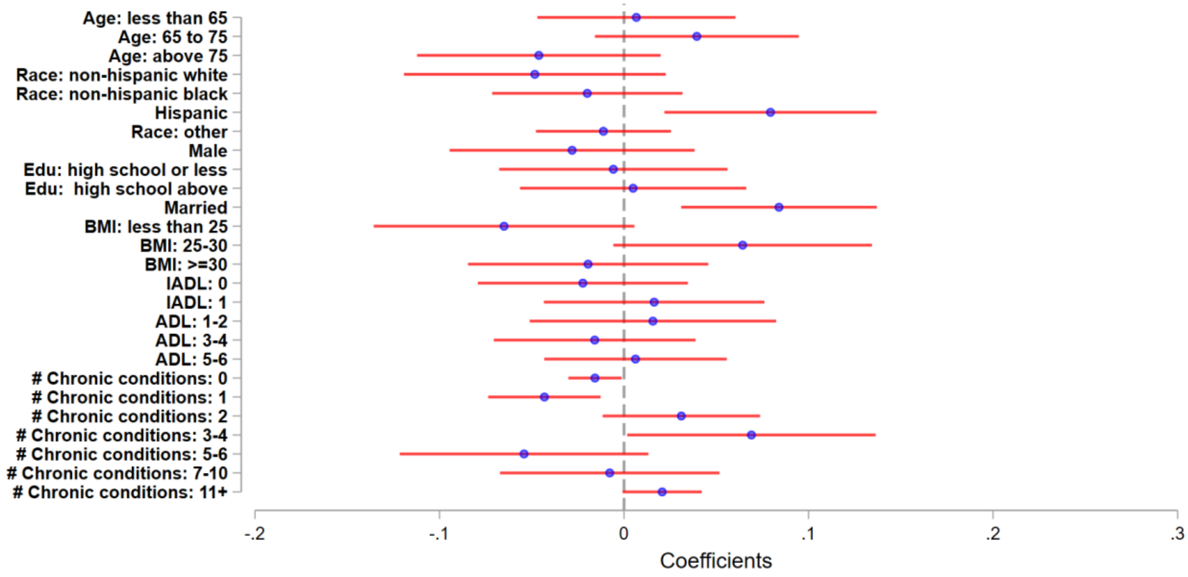


*Notes:* The working sample consists of MA beneficiaries from 2015-2022 MCBS. Beneficiaries with only partial enrollment in an MA plan during the past year at the time of the survey and veteran are excluded. The plotted estimates represent the coefficients for the interaction term between the treatment indicator and the post indicator from a DID estimation, with the dependent variable indicated on the y-axis. In each DID estimation, the treatment group consists of MA beneficiaries with ADRD, and the control group consists of MA beneficiaries without ADRD but with stroke/brain hemorrhage, complete/partial paralysis, or Parkinson's diseases. The post indicator takes the value of one if 2020 or afterwards, and zero otherwise. The number of chronic conditions excludes ADRD. The associated 95% CIs are plotted. Robust standard errors are applied.

**eFigure 13. Dynamic Association between the Inclusion of ADRD HCCs in Payment Model and Beneficiaries' Characteristics – Test for Compositional Change**

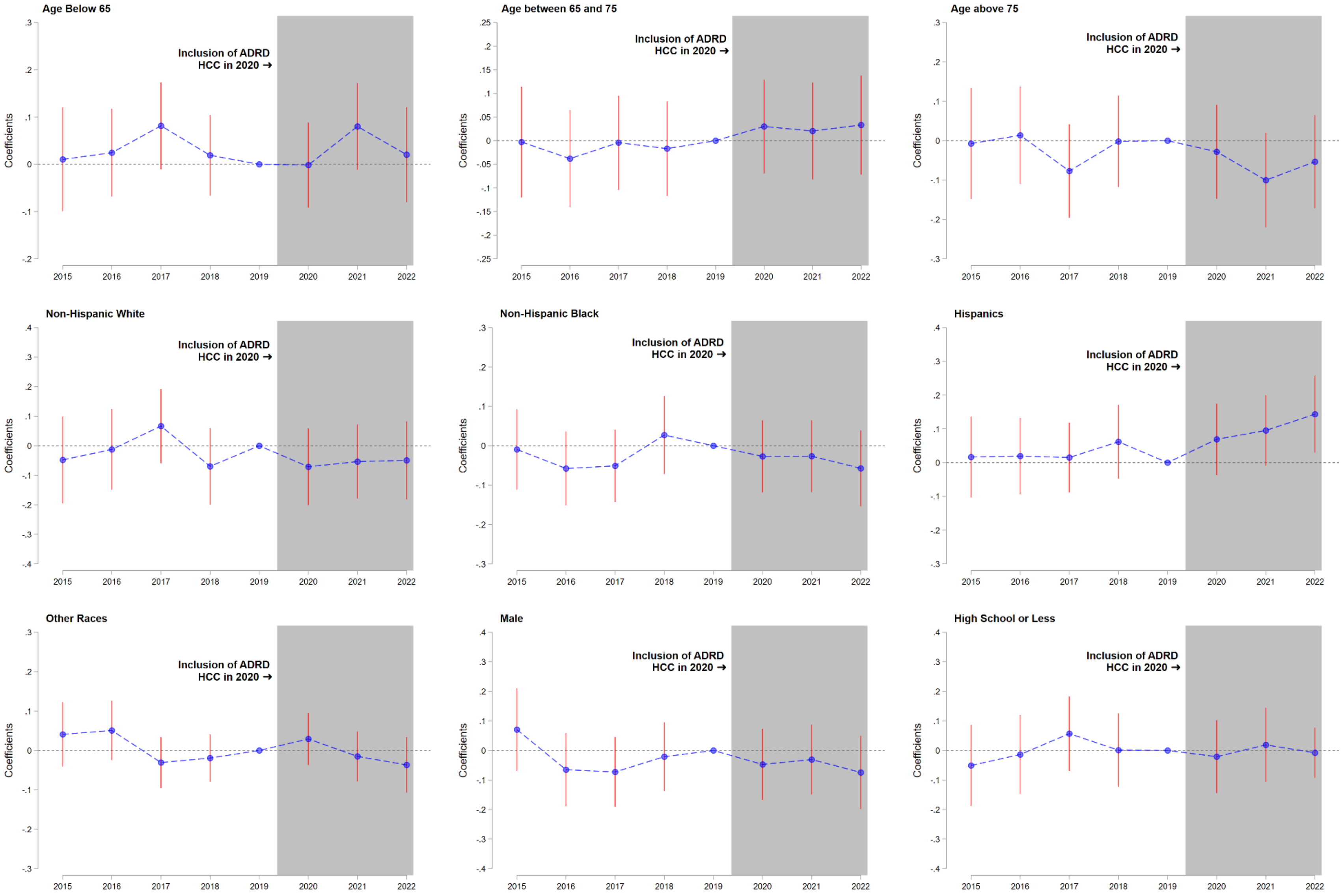


© 2026 Fu W et al. *JAMA Netw. Open*.

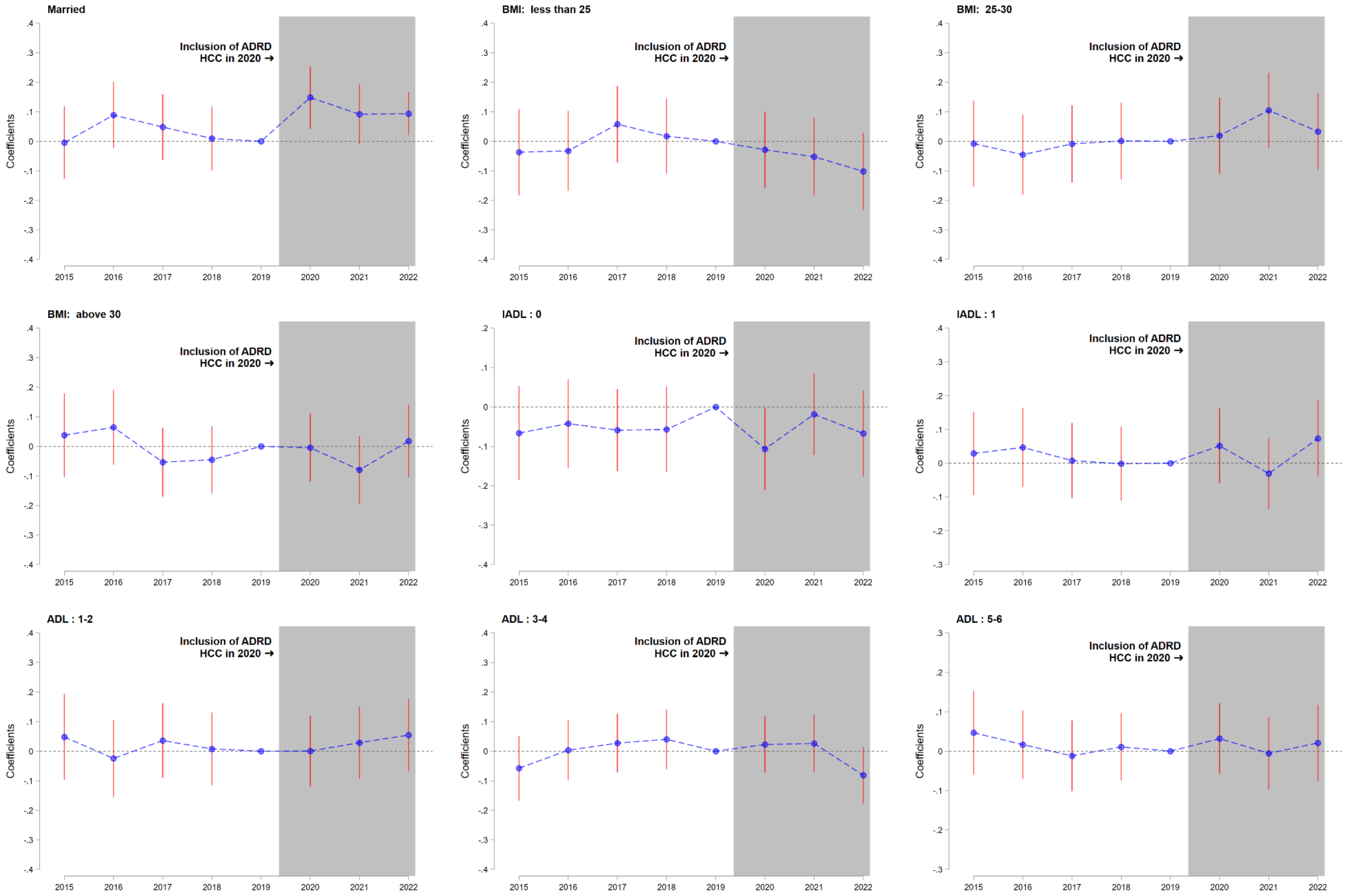


© 2026 Fu W et al. *JAMA Netw. Open*.

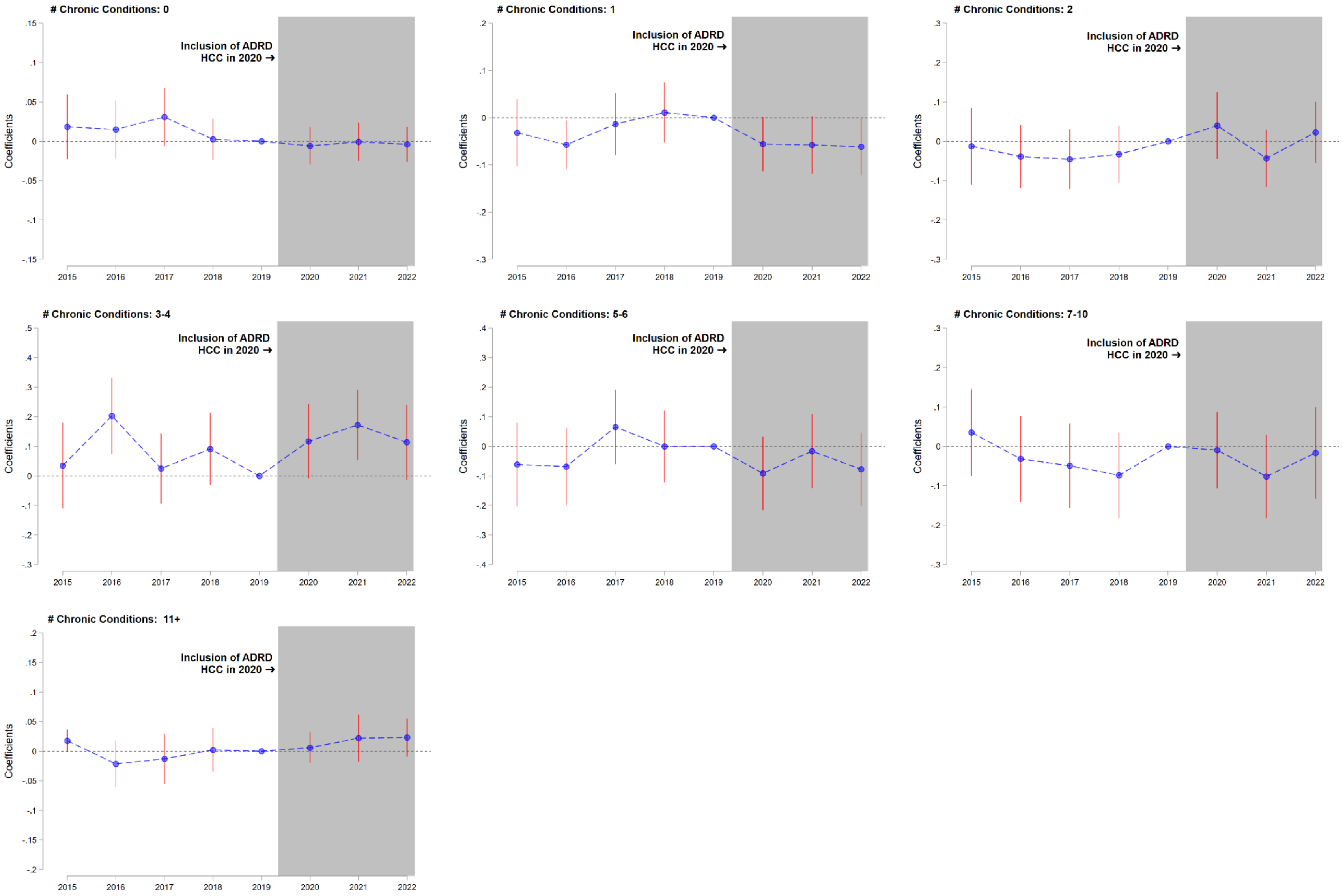


*Notes:* The working sample consists of MA beneficiaries from 2015-2022 MCBS. Beneficiaries with only partial enrollment in an MA plan during the past year at the time of the survey and veteran are excluded. Each figure presents results from an event study model, with the dependent variable specified above. The plotted estimates represent the coefficients for the interaction term between treated indicator and year indicators. In each estimation, the treatment group consists of MA beneficiaries with ADRD, and the control group consists of MA beneficiaries without ADRD

© 2026 Fu W et al. *JAMA Netw. Open*.

but with stroke/brain hemorrhage, complete/partial paralysis, or Parkinson's diseases. The number of chronic conditions excludes ADRD. The associated 95% CIs are also plotted. Robust standard errors are applied.

© 2026 Fu W et al. *JAMA Netw. Open*.
