# Supplementary material for: Risk Adjustment for Alzheimer Disease and Related Dementias in Medicare Advantage and Health Care Experiences
Source: JAMA Netw Open. 2026 Mar 13;9(3):e261796. doi: 10.1001/jamanetworkopen.2026.1796 (PMC12988443; doi:10.1001/jamanetworkopen.2026.1796)
Supplement: Supplement 2. — Data Sharing Statement [file jamanetwopen-e261796-s002.pdf]

Data Sharing Statement

Fu. Risk Adjustment for Alzheimer Disease and Related Dementias in Medicare Advantage and Health Care Experiences. *JAMA Netw Open*. Published March 13, 2026.
doi:10.1001/jamanetworkopen.2026.1796

Data
Data available: Yes
Data types: Data (not involving human participants)
How to access data: xi.chen@yale.edu
When available: With publication

Supporting Documents
Document types: Statistical/analytic code
How to access documents: xi.chen@yale.edu
When available: With publication

Additional Information
Who can access the data: researchers whose proposed use of the data has been approved
Types of analyses: for any purpose
Mechanisms of data availability: with a signed data access agreement
